# Chronic kidney disease induces distinct alterations of macrophage lipid metabolism in a mouse model of atherosclerosis

**DOI:** 10.1016/j.jlr.2026.100975

**Published:** 2026-01-02

**Authors:** Keith Saum, Xinyi Liu, Thekkelnaycke Rajendiran, Lixia Zeng, Pradeep Kayampilly, Jaeman Byun, Farsad Afshinnia, Subramaniam Pennathur

**Affiliations:** 1 Division of Nephrology, Department of Internal Medicine, University of Michigan, Ann Arbor, MI; 2 Department of Computational Medicine and Bioinformatics, University of Michigan, Ann Arbor, MI; 3 Michigan Center for Translational Pathology, University of Michigan, Ann Arbor, MI; 4 Molecular and Integrative Physiology, University of Michigan, Ann Arbor, MI

**Keywords:** glycerophospholipid, inflammation, kidney disease, lipidomics, macrophages

## Abstract

Chronic kidney disease (CKD) is associated with altered lipid metabolism and chronic inflammation, which both contribute to an accelerated risk of atherosclerotic cardiovascular disease. Macrophage polarization towards a pro-inflammatory phenotype plays a key role in atherosclerotic cardiovascular disease development and is mediated by a rewiring of macrophage immunometabolism. While prior studies have investigated associations between the systemic lipidome and CKD-accelerated CVD, the impact of CKD on macrophage lipid metabolism remains unknown. In this study, we profiled the macrophage lipidome in mice with and without CKD induced by 5/6 nephrectomy. After 16 weeks of a high-fat diet, thioglycollate-elicited peritoneal macrophages (PMΦ) were collected and subjected to lipidomics by LC-MS/MS. Quantification of 481 distinct lipids across 19 lipid classes identified an increased abundance of saturated C16-C24 FFAs, phosphatidylglycerols, phosphatidylethanolamines, modified ceramides, and polyunsaturated ether lipids in PMΦ from CKD mice compared to controls. PMΦ from CKD mice also exhibited decreased abundance of unsaturated FFAs, triglycerides and phosphatidylcholines. Long-chain–to–intermediate-chain acylcarnitine ratio, a metric of β-oxidation efficiency, was reduced in CKD PMΦ, without altering macrophage de novo lipogenesis suggesting a shunting of exogenous lipids towards complex lipid synthesis. Pathway enrichment analysis identified long-chain acyl-CoA synthetase 1 (ACSL1) as a potential upstream mediator of these observed changes in macrophage lipid metabolism. Expression of *Acsl1* and inflammatory cytokines was increased in CKD PMΦ or following treatment with palmitate or uremic serum in RAW 264.7 macrophages. These effects were blunted by the knockdown of ACSL1 in RAW264.7 cells. Partitioning of fatty acids towards complex lipid synthesis by ACSL1 may be a mechanism underlying chronic inflammation in advancing CKD.

Chronic kidney disease (CKD) is a complex, multisystem disorder characterized by progressive loss of kidney function, chronic inflammation, and profound disturbances in lipid metabolism. Compared with healthy individuals, the serum lipid profiles of patients with moderate to advanced CKD are typically characterized by increased triglycerides, decreased HDL cholesterol, and structurally or functionally modified LDL, such as oxidized LDL or carbamylated LDL (1). Fatty acid metabolism is also altered in CKD, and experimental studies have revealed that mitochondrial dysfunction and fatty acid overload are closely linked with CKD progression and inflammation (2, 3). These lipid abnormalities play a key role in amplifying systemic inflammation and oxidative stress, which contributes to the heightened risk of atherosclerotic cardiovascular disease (ASCVD) observed in CKD patients. However, despite advances in lipid-lowering therapies, ASCVD remains the leading cause of death in CKD, underscoring the need to better understand how lipid dysregulation in CKD contributes to inflammation and resultant ASCVD (4).

The accumulation of macrophage foam cells in the arterial wall is a key process in atherosclerosis development and progression. The uptake of oxidized LDL results in macrophage activation and polarization towards a proinflammatory (M1) phenotype defined by the production of reactive oxygen species and cytokines such as IL-1, IL-6, and TNF-α (5, 6). To achieve this immune response, macrophages undergo robust reprogramming of intracellular metabolism to redirect nutrients towards cell proliferation and cytokine production (7). Classically activated M1 macrophages exhibit increased fatty acid synthesis to produce complex glycerophospholipids required for cell membrane remodeling and inflammatory signaling. In addition, CKD also affects macrophage lipid metabolism directly and indirectly. Direct effects include the upregulation of FFA and lipoprotein scavenger receptors, with a simultaneous decrease in cholesterol efflux (8, 9). Indirect effects include the accumulation of modified LDL and HDL lipoproteins in CKD, which render these particles more susceptible to macrophage scavenging. These effects promote foam cell formation, leading to more complex atheromatous plaques with increased macrophage burden in CKD.

Despite the current understanding of macrophage immune metabolism in the development of ASCVD, lipidomic phenotyping of macrophages in CKD has been absent to date. Prior studies have examined the impact of CKD on the plasma lipidome, including aberrations in plasma lipoproteins and lipid species (10, 11). Our group has shown progressive differences in the human plasma lipidome from multiple CKD cohorts with and without diabetes, including an increased abundance of short C16–C20 saturated FFA and long polyunsaturated complex lipids in the later stages of CKD, which were inversely associated with impaired β-oxidation as assessed by acylcarnitine ratio (3, 12). However, it remains unknown whether alterations in the macrophage lipidome parallel those in plasma. Further understanding of macrophage lipid metabolism in CKD could lead to novel biomarkers or therapeutic approaches to mitigate inflammation and cardiovascular risk.

In the present study, we investigated changes in the macrophage lipidome in a murine model of CKD-accelerated atherosclerosis. Lipidomics analysis of 911 lipids revealed distinct alterations of multiple lipid classes in elicited peritoneal macrophages (PMΦ) from CKD mice, which were associated with a reduced acylcarnitine ratio. Pathway analysis identified long-chain acyl-CoA synthetases (ACSLs) as potential upstream mediators of these changes in macrophage lipid metabolism. We found that acyl-CoA synthetase 1 (ACSL1) was upregulated in PMΦ of CKD mice, concomitant with the upregulation of well-known inflammatory mediators. Furthermore, targeted deletion of ACSL1 in RAW264.7 cells inhibited the upregulation of proinflammatory genes induced by palmitate or uremic serum. These findings provide novel insights into how CKD alters macrophage lipid metabolism and resultant chronic inflammation seen in CKD.

## Materials and Methods

### Mouse model of CKD-accelerated atherosclerosis

The University of Michigan Institutional Animal Care and Use Committee approved all animal procedures. Male C57BL/6 LDLr^−/−^ mice (The Jackson Laboratory) were maintained with water ad libitum and on a standard rodent diet (5L0D, LabDiet) and housed in a climate-controlled, light-regulated facility with a 12:12 h light/dark cycle. At age 6 weeks, the mice were subjected either to a sham operation (CTL, n = 20) or 5/6 nephrectomy by removing the whole right kidney in a first procedure, followed by two-thirds of the left kidney by renal artery ligation (CKD, n = 20) after 1 week, as previously described by our group (13, 14, 15). At 9 weeks of age, mice were transitioned to a high-fat diet (HFD) containing 19.5% protein, 40.5% fat with 0.5% cholesterol, and 40.0% carbohydrates by weight (Harlan Teklad Laboratory). Mice were maintained on HFD for 16 weeks before tissue collection and evaluated in a blinded manner.

Hematocrit was measured using a StatSpin CritSpin Micro-Hematocrit centrifuge with a digital hematocrit reader (Beckman Coulter). Plasma creatinine levels were measured by highly specific electron spray ionization and tandem LC-MS, as previously described (15). Plasma intact parathyroid hormone was measured by ELISA (ALPICO Diagnostics). Plasma phosphorus was measured by colorimetric assay using the manufacturer’s instructions with a 1:1,000 dilution of plasma. (Sigma Aldrich). BUN was measured directly on an iSTAT-1 point-of-care chemistry analyzer (Abbott Laboratories). Serum was isolated from 10 CKD and 10 sham mice by cardiac puncture and allowed to clot at room temperature. Serum from CKD or sham mice was pooled and heat-inactivated for 30 min at 50°C to generate uremic or control serum, respectively, for tissue culture studies.

### Peritoneal macrophage isolation

Following 16 weeks of HFD, mice were injected intraperitoneally with 2 ml of 3% wt/vol thioglycollate medium prepared in sterile saline. Elicited PMΦ were collected three days after thioglycollate injection by peritoneal lavage with RPMI-1640 medium. PMΦ isolates were filtered with a 100 μm strainer and pelleted by centrifugation prior to incubation with RBC lysis buffer for 5 min to remove contaminating red blood cells. Macrophages were adherence-purified for 1 h, followed by washing with PBS to remove nonadherent cells. Purified PMΦ were then collected by mechanical removal with a cell scraper and washed in ice-cold PBS prior to cell counting. PMΦ cell pellets were flash frozen in liquid nitrogen and stored at −80°C prior to lipid extraction.

Thioglycollate-elicited cells were analyzed by flow cytometry to assess macrophage purity. Isolated cells were incubated with Fc-block (anti-CD16/32, BD Biosciences) for 10 min to prevent nonspecific Fc-mediated binding, and then stained with fluorochrome-conjugated antibodies against CD11b, F4/80, GR1, CD3, B220, and Siglec-F. Dead cells were excluded using a fixable viability dye. Flow cytometry was performed on a Bio-Rad Ze5, and data was analyzed using FlowJo (v10.10). Singlets were gated using FSC-A versus FSC-H, and macrophages were identified as CD11b^+^ F4/80^+^, with GR1^+^ used to distinguish neutrophil and monocytes. B cells, T cells, and eosinophils were distinguished by B220^+^, CD3^+^, or Siglec-F^+^ staining, respectively.

### Lipidomic analysis

Details of sample preparation and lipid identification for the lipidomics platform have been previously published (16). Briefly, we extracted lipids from peritoneal macrophage cell pellets or 50 μl of plasma using a modified Bligh and Dyer method for lipidomics and acylcarnitine measurements. Lipids were extracted with water:methanol:dichloromethane (2:2:2) after spiking each sample with a mixture of 13 internal standards. Extracts were dried under nitrogen and reconstituted in 0.1 ml of 10 mM ammonium acetate:dichloromethane:methanol. LC/MS/MS was performed with a Shimadzu UHPLC and AB Sciex 6500 QTRAP with TurboIonSpray ion source operating in multiple reaction monitoring (MRM) mode with scans performed in positive and negative ion modes.

The targeted lipidomic platform was used to measure the abundance of 911 lipids across 20 lipid classes including cholesterol esters (CE, n = 19), monoacylglycerols (MAG, n = 1), diacylglycerols (DAG, n = 39), triacylglycerols (TAG, n = 445), FFAs, n = 16), phosphotidylcholines (PC, n = 65), phosphotidylethanolamines (PE, n = 47), PE with alkyl ether substitute at sn-1 carbon (PE-O n = 19), PE with alkenyl ether substitute at the sn-1 carbon (PE-P, n = 41), phosphatidylinositol (PI, n = 22), phosphotidylcholine (PC, n = 65), phosphatidylglycerols (PG, n = 24), lysophosphatidylcholine (LPC, n = 11), phosphotidylserine (PS, n = 26), lysophosphotidylserine (LPS, n = 1), lysophosphatidylethanolamine (LPE, n = 10), sphingomyelin (SM, n = 27), hexosylceramide (HCER, n = 9), lactosylceramide (LCER, n = 6), dihydroceramide (DCER, n = 6), (Supplemental Table S1). The quality control included running pooled samples at the beginning of the run and after every 10 runs to assess sample drift and calculate the coefficient of variation of the measurements. Ceramides (CER, n = 10) and acylcarnitines (n = 29) were quantified by LC/MS using an Agilent 6490 Triple Quadrupole tandem mass spectrometer (Agilent, Santa Clara, California) with targeted methods described previously (17, 18).

### In vivo assessment of de novo lipogenesis

To assess relative differences in de novo lipogenesis between sham and CKD mice, we administered mice deuterated water (^2^H_2_O) to isotopically label newly synthesized palmitic acid, the primary product of fatty acid synthase, with ^2^H. Intake of ^2^H_2_O results in universal labeling of biomolecules including lipids, making it ideally suited for assessment of synthetic rates. Mice were given intraperitoneal boluses of ^2^H_2_O (20 μl/gm body weight) 24 and 72 h prior to tissue collection. Following the first ^2^H_2_O bolus, mice were also maintained on 5% ^2^H_2_O in drinking water until the time of tissue collection. Lipids were extracted from plasma, PMΦ, or liver samples as described above. The organic layer containing lipids was collected, hydrolyzed to liberate palmitate, and analyzed by LC/MS. De novo lipogenesis was measured as a function of percentage ^2^H enrichment of palmitate, as previously described, using an Agilent 6546 Q-TOF mass spectrometer, coupled to an Agilent 1290 LC (19). Total palmitate enrichment in ^2^H was determined from mass isotopomers (M+1, M+2, M+3, and M+4). The acquired raw data were processed using Mass Hunter Profinder (Agilent Profinder V10). The percent labeling of palmitate was quantified by measuring the peak area of each mass shift relative to unlabeled palmitate, and the de novo lipogenesis rate was determined from total palmitate ^2^H enrichment = ^2^H_1_ + (^2^H_2_ × 2) + (^2^H_3_ × 3) + (^2^H_4_ × 4). Each sample was measured in triplicate, and the average was used for the value of the sample.

### Cell culture

RAW264.7 and ACSL1 KO RAW264.7 cell lines were obtained from EditGene (Guangzhou, China, Catalog No. EDC07675, Clone EDG20252515) and cultured at 37°C in DMEM containing 10% FBS and 100 U/ml antibiotics cocktail (cat. # 15070063; Thermo Fisher Scientific) in a 5% CO_2_ incubator. Cells were split using a cell scraper after reaching 70–80% confluency and seeded at 1 × 10^6^ cells/cm^2^ 24 h prior to stimulation with either 200 mM palmitic acid (16:0) bound to BSA, BSA alone, 10% uremic mouse serum, or 10% control mouse serum for 24 h.

### Western blotting

Protein lysates were prepared from PMΦ cell pellets, and the protein concentration in each sample was determined using a bicinchoninic acid assay kit. Thirty μg of total protein from each sample was denatured at 95°C for 5 min and resolved by SDS-PAGE prior to transfer to polyvinylidene fluoride membranes for 1 h at 200 mA constant voltage. The membrane was incubated in Everyblot blocking buffer (BioRad) for 1 h at room temperature. Afterward, the membrane was incubated with primary antibody for ASCL1 (cat. # 10585, Cell Signaling Technology) or GAPDH at 1:1,000 dilution overnight at 4°C. Washes were performed with Tris-buffered saline with 0.1% Tween 20. After washing, membranes were probed with HRP-linked anti-rabbit IgG for 1 h at room temperature. The signal was visualized using a chemiluminescent imaging system with a 15-min exposure.

### RT-PCR

Total RNA was extracted from cell pellets using the Purelink RNA Mini Kit (Thermo Fisher Scientific, cat # 12183020) with on-column DNAse treatment. RNA concentration and purity were tested using a NanoDrop 2000 spectrophotometer. Complementary cDNA was synthesized using a High-Capacity cDNA Reverse Transcription Kit (Applied Biosystems, cat # 4368814). Quantitative RT-PCR was performed using Taq-Man Fast Advanced Master Mix and Taqman Primers for mouse *Acsl1, Acsl3, Acsl4, Acsl5, Acsl6,* *Il-1* *beta,* *Tnf-alpha,* *Il-6,* and *Gapdh* on a QuantStudio 3 RT-PCR system. A total of 100 ng of cDNA was used for each RT-PCR reaction, and reactions were performed in quadruplicate. *Gapdh* was used as a housekeeping gene. Relative changes in mRNA expression were calculated using the ΔΔCT method.

### Data analysis

Individual lipid species were quantified by taking the ratio of peak areas for the lipid and corresponding lipid class internal standard, followed by multiplying by the concentration of the internal standard. Quantified lipids were normalized to the total protein content of the macrophage cell pellet, determined by BCA assay from the aqueous phase of the lipid extraction preparation. Plasma lipids were normalized to sample volume (50 μl). K-nearest neighbors imputation was applied for missing lipids below 50%. We combined the abundance of TAG isotopomers into one lipid feature, which reduced total triacylglycerols (TAGs) from 445 to 95. Sum normalization by lipid class was then performed before logit transformation and z-score normalization. To determine the effect of CKD on lipid changes, we applied linear models for each primary lipid class with CKD status treated as a categorical variable. All data processing and statistical analysis were conducted using R software (https://www.r-project.org/).

To compare the relationship between lipid class abundance in CKD and sham macrophages, we first performed pairwise Pearson correlation for all 481 unique lipids, as previously described (20). In this part of the analysis, we used Fisher Z-transformation to calculate correlation *P*-values statistically different from 0. We set α = 0.05 and adjusted all correlation coefficient *P*-values using the Benjamini-Hochberg FDR correction to control the type I error rate. The correlation between lipid pairs is considered statistically significant if the adjusted *P*-value is smaller than the pre-specified threshold. Heatmaps were generated to visualize the correlation between lipids. To further uncover lipids differentially correlated with saturated and unsaturated FFA levels between control and CKD PMΦ, we applied debiased sparse partial correlation (DSPC) analysis using Metaboanalyst 6.0 to independently construct metabolic correlation networks for CKD and sham groups, as previously described (21). A *P*-value of 0.1 was used to define significant edges in each network. To evaluate differences in DSPC networks, we performed Fisher’s exact test on contingency tables of significant and non-significant edges for both saturated and unsaturated FFA. Significant FFA correlations were visualized as correlation-based networks in Cytoscape using Metscape software (https://metscape.ncibi.org/).

We also performed pathway and network analyses to identify potential lipid metabolic pathways and upstream regulators predictive of the observed changes in the macrophage lipidome. The HMDB identifiers for analyzed lipids were downloaded from the HMDB database (https://hmdb.ca/). Pathway and network analysis were performed using Ingenuity Pathway Analysis software (IPA, https://analysis.ingenuity.com/). We took − log (*P* value) > 2 as the threshold for potential upstream regulators and then defined Z score > 2 and Z score < −2 as the threshold of significant activation and inhibition, respectively.

## Results

### Nephrectomized LDLr^−/−^ mice have features of moderate CKD

C57BL/6 LDLr^−/−^ mice were subjected to 5/6 nephrectomy between 6 and 8 weeks of age. After one week of recovery, mice were transitioned to a high-fat/high-cholesterol diet (HFD) for 16 weeks. Mice from both groups gained significant body weight over time (Table 1). CKD mice weighed less than control mice at baseline, but there was no difference in body weight between the two groups at the end of 16 weeks on an HFD. Serum creatinine and BUN levels in the CKD-HFD mice were elevated at 16 weeks of HFD, confirming renal insufficiency. CKD mice also displayed other features of renal insufficiency, such as anemia with significantly lower hematocrit after 16 weeks of HFD. There were no differences in serum phosphorus, total cholesterol, triglycerides, or intact parathyroid hormone levels between the two groups. CKD status also did not affect immune cell recruitment in thioglycollate-elicited peritoneal cell isolates, as determined by flow cytometry. A representative gating strategy for identification of leukocyte fractions in peritoneal isolates is shown in Supplemental Figure S1.

**Table 1:** Biological characteristics of the CKD mouse model

Biological parameters^a^	N (per group)	CTL-HFD (μ ± sd)	CKD-HFD (μ ± sd)	*P*-value^b^
Body weight (grams)				
Baseline	18	23.1 ± 2.8	20.3 ± 2.5	0.0033
16 weeks (HFD)	18	36.6 ± 2.8	33.8 ± 1.8	0.0771
Plasma creatinine (mg/dl)	18	0.13 ± 0.04	0.33 ± 0.06	<0.0001
Plasma BUN (mg/dl)	18	28.4 ± 3.7	54.7 ± 5.4	<0.0001
Hematocrit (%)	18	61.5 ± 6.2	47.9 ± 4.9	<0.0001
Serum phosphorus (mg/dl)	10	10.3 ± 1.5	11.3 ± 1.8	0.1939
Intact parathyroid hormone (pg/ml)	10	14.4 ± 7.6	12.4 ± 6.7	0.5403
Plasma total cholesterol (mg/dl)	10	1,164 ± 90	1,185 ± 51	0.5415
Plasma triglycerides (mg/dl)	10	303 ± 69	353 ± 61	0.2377
Thioglycollate-elicited cells (% of total)				
Macrophages (CD11b^+^, F4/80^+^)	5	74.9 ± 10.5	83.5 ± 7.0	0.1267
Neutrophils/Monocytes (CD11b^+^,GR1^Hi^)	5	3.6 ± 1.3	2.3 ± 1.6	0.1142
Eosinophils (Siglec-F^+^)	5	2.5 ± 0.6	2.0 ± 0.4	0.3079
B cells (B220^+^)	5	0.7 ± 0.5	0.4 ± 0.4	0.3394
T cells (CD3^+^)	5	14.1 ± 7.1	17.8 ± 7.4	0.4767

BUN, blood urea nitrogen; CKD, chronic kidney disease; CTL, control; HFD, high-fat diet.

a All measures obtained after 16 weeks of high-fat diet, unless otherwise indicated.

b T-test between CTL-HFD and CKD-HFD groups.

### CKD alters the macrophage lipidome in a tissue-specific manner

**Fig. 1:**
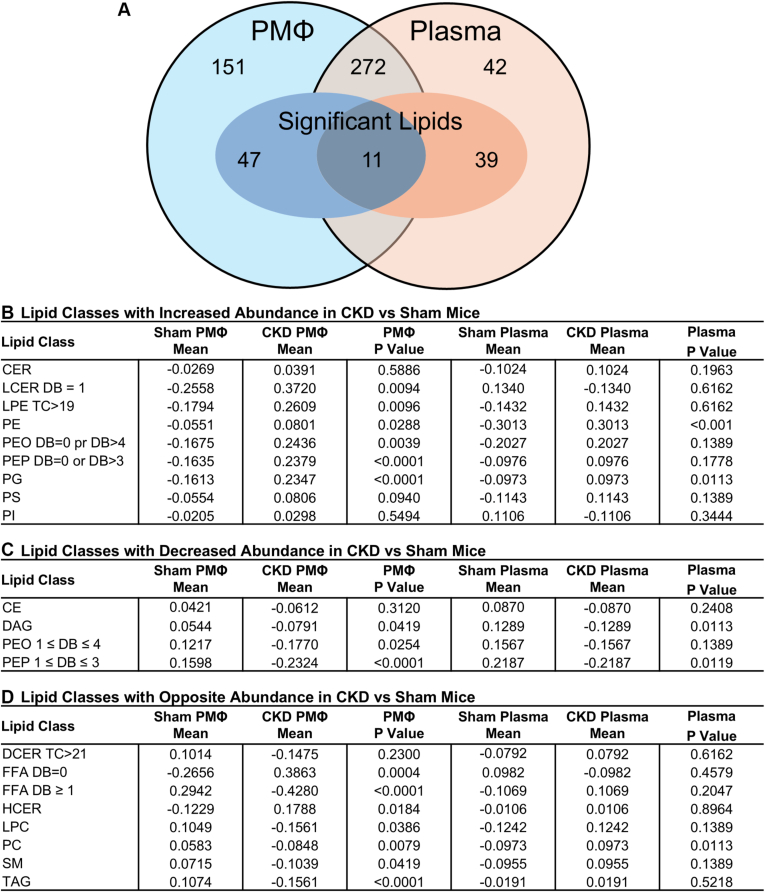
Differential lipid changes in CKD mouse peritoneal macrophages and plasma. Targeted lipidomics identified 364 unique lipids in plasma and 481 lipids in peritoneal macrophages from CKD and sham mice after 16 weeks of high-fat diet (n = 10–12/group). A: Venn diagram of unique and significant lipid features in plasma and PMΦ. Outer circles depict the total number of unique lipids identified in each tissue. Inner ovals represent the number of significantly altered lipid features in CKD compared to sham mice. B–D: Lipid classes with increased (B), decreased (C), and opposing (D) abundances in CKD peritoneal macrophages and plasma compared to sham. Values are z-score standardized mean levels per lipid class. *P* values for the effect of CKD status on lipid class level were calculated from linear mixed models and adjusted for multiple comparisons. CKD, chronic kidney disease; PMΦ, peritoneal macrophages.

Targeted lipidomics was used to determine differences in complex lipid classes present in plasma and peritoneal macrophages from sham and CKD mice after 16 weeks of HFD. Measured lipids are shown in Supplemental Table S1. A total of 481 unique lipid features were detected after sum normalization of TAG species, with 272 lipid features present in both plasma and PMΦ (Fig. 1A). The number of lipids that were significantly altered (*P* < 0.01) between sham and CKD mice were similar in each sample type, with 50 (13.7%) in plasma and 58 (12.0%) in PMΦ. Of the 272 unique lipid features present in both tissues, only 11 were significantly different between sham and CKD mice in both plasma and PMΦ (Supplemental Table S2). Despite these similarities, changes in lipid features by lipid class were distinctly different between plasma and PMΦ, and the direction of change in CKD was often inconsistent. To reduce dimensionality due to the high number of lipids identified relative to the low sample size, we grouped individual lipid features by saturation or acyl-chain length within each class and examined the biologically relevant subclasses between plasma and PMΦ using linear models with CKD status as a categorical variable. Overall, the mean level of saturated FFA and multiple classes of glycerophospholipids, including PG, PE, and saturated PE-O and PE-P, were significantly elevated in PMΦ from CKD mice compared to controls (Fig. 1B). However, only significant increases in PG, PE, and PC were observed in CKD plasma. Conversely, CKD PMΦ had lower mean levels of unsaturated FFA, TAG, DAG, SM, PC, and polyunsaturated PE-O and PE-P than those from control mice (Fig. 1C–D). Interestingly, many changes in PMΦ lipid abundance by class were opposite to those seen in the plasma, including saturated and unsaturated FFA, TAG, and PC. Changes of each lipid molecule within different lipid classes in the CKD and control groups are illustrated in Supplemental Figures S2-S21.

### Lipid class correlation analysis

**Fig. 2:**
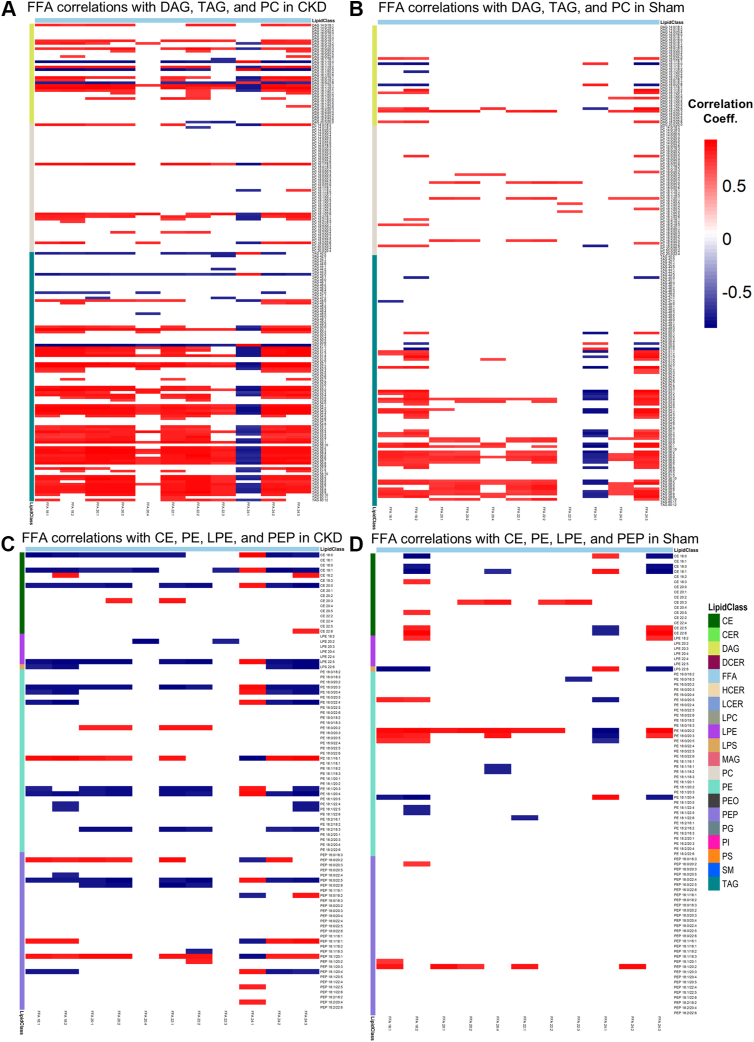
Correlation of unsaturated FFA levels and glycerolipids by class in sham and CKD peritoneal macrophages. Pearson correlation coefficients for unsaturated FFA and each lipid by class are shown for CKD (A, C) and sham (B, D) peritoneal macrophages. Significant correlations, |>0.7|, are shown as positive (red) or negative (blue). *P* < 0.05 using Pearson’s correlation with Fisher’s transformation and Benjamini-Hochberg FDR correction. CKD, chronic kidney disease.

Identifying differential correlations between lipid classes can provide valuable information regarding network connectivity and alterations in lipid metabolic pathways, even when individual lipid values are not significantly different between groups. Based on the distinct differences in saturated and unsaturated FFA levels between CKD and sham mice, we were specifically interested in assessing lipid class correlations between both saturated and unsaturated FFA in control and CKD mice PMΦ. Pearson correlation analysis showed multiple differences between control and CKD PMΦ (Fig. 2). Standardized levels of TAG, DAGs, and polyunsaturated PC were all positively correlated with unsaturated FFA in CKD PMΦ compared to controls. Conversely, unsaturated FFA levels were negatively correlated with cholesterol esters, and polyunsaturated lysophosphatidylethanolamines, lysophosphotidylserines, PE, and PEP glycerophospholipids in CKD PMΦ compared to controls. Correlations of phospholipids with saturated FFA were less pronounced, with increased PE abundance correlated with saturated FFA in CKD macrophages compared to controls (Supplemental Figure S22). DSPC correlation networks for unsaturated and saturated FFA are shown in Supplemental Figure S23. Contingency analysis comparing CKD to sham correlations networks revealed a loss of unsaturated FFA and complex lipid interactions in CKD PMΦ compared to controls. No significant interactions were observed for saturated FFA in sham or CKD PMΦ.

### Alteration of macrophage fatty acid metabolism by CKD status

**Fig. 3:**
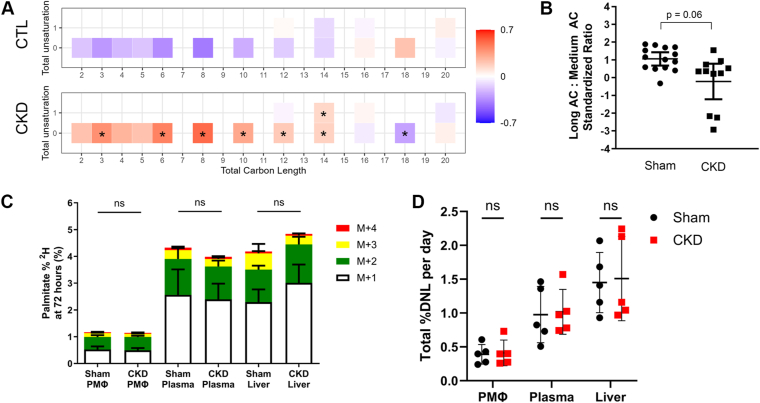
Assessment of macrophage beta oxidation and fatty acid synthesis in sham and CKD mice. Peritoneal macrophage acylcarnitines were quantified by targeted LC/MS analysis (n = 11/group) and used to calculate long-to-intermediate chain acylcarnitine ratio as a metric of fatty acid beta oxidation. A: Mean standardized levels of macrophage acylcarnitines in sham and CKD mice. Values are z-score standardized mean acylcarntine levels. ∗*P* < 0.05 compared to sham AC level by *t* test. B: Calculated long-to-intermediate acylcarnitine ratio in CKD peritoneal macrophages compared to controls. C, D: Tissue de novo lipogenesis was measured following deuterated water administration to isotopically label synthesized palmitate. C: Contributions of ^2^H_1_, ^2^H_2_, ^2^H_3_, and ^2^H_4_ isotopomers to palmitate enrichment after 72-h (n = 5 mice per group). Contributions calculated as 1 × ^2^H_1_, 2 × ^2^H_2_, 3 × ^2^H_3_, and 4 × ^2^H_4_ fractional isotopomer distributions, as described in the Methods section. D: de novo lipogenesis rates were similar in CKD and sham mice between PMΦ, plasma, and liver tissues (n = 5/group). CKD, chronic kidney disease; PMΦ, peritoneal macrophages.

Impaired beta oxidation due to mitochondrial dysfunction is common in CKD and manifests as a reduced ratio of long (C16-C20) to intermediate (C5-C14) AC. To determine whether macrophages exhibit impaired beta oxidation in CKD, we quantified AC in PMΦ from CKD and control mice after 16 weeks of HFD. CKD PMΦ had higher mean standardized levels of intermediate AC compared to controls, with lower mean abundance of long-chain AC, particularly C18:0 AC (Fig. 3A). The long-chain to intermediate-chain AC ratio in CKD PMΦ was diminished compared to controls, although it did not reach statistical significance (*P* = 0.061, Fig. 3B). This suggests that although CKD impacts macrophage β oxidation, mitochondrial dysfunction is not the sole reason for the diversion of fatty acids towards complex lipid synthesis in CKD macrophages.

To further assess whether changes in systemic de novo lipogenesis contribute to the observed changes in tissue FFA, we administered ^2^H_2_O to mice to isotopically label newly synthesized palmitic acid. As liver lipid composition is arguably the most likely to reflect plasma composition, we also included analysis of de novo lipogenesis in liver tissue to look for broader trends and insight into whether similarities are more systemic. Quantification of ^2^H palmitate enrichment up to the M+4 isotopomer was observed in liver, plasma, and PMΦ tissues from sham and CKD mice to an equal extent (Fig. 3C). Total rates of de novo lipogenesis were also similar across liver, plasma, and PMΦ tissues from sham and CKD mice (Fig. 3D).

### Pathway analysis identifies Acyl-CoA synthetases as potential upstream mediators of macrophage lipid alterations in CKD

**Fig. 4:**
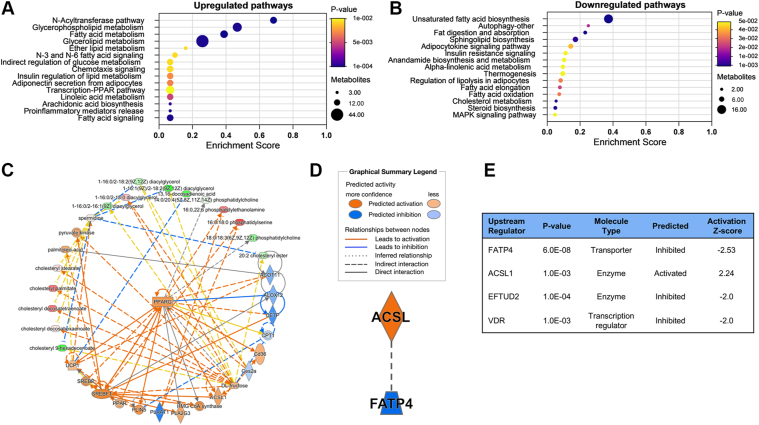
Lipidomic pathway analysis identifies ACSL1 as potential upstream regulator of macrophage lipid metabolism in CKD. A, B: Pathway analysis for lipids with increased (A) and decreased (B) levels in CKD PMΦ. Dot size represents number of lipids with differential expression compared to controls. Dot color indicates FDR-adjusted *P*-value. C: Predicted regulatory network from IPA analysis identified activation of multiple upstream regulators of lipid metabolism. D: Upstream regulator analysis identified 4 significant regulators as potential causal mediators for lipidomic signature in CKD PMΦ, including ACSL1, fatty acid transport protein 4, elongation factor Tu GTP binding domain containing 2, and vitamin D receptor. E: IPA graphical summary predicts from upstream regulatory analysis. ACSL1, long chain acyl-CoA synthetase 1; CKD, chronic kidney disease; PMΦ, peritoneal macrophages.

Pathway enrichment analysis was used to predict metabolic pathways based on lipids with increased and decreased levels in CKD PMΦ. As shown in Figure 4A, the lipids with increased levels in CKD PMΦ were associated with glycerophospholipid metabolism, fatty acid metabolism, and fatty acid signaling. Interestingly, lipids with decreased levels were mainly associated with PUFA biosynthesis, autophagy, fat digestion and absorption, sphingolipid metabolism, insulin resistance, thermogenesis, fatty acid oxidation, and steroid biosynthesis (Fig. 4B). These results further support a reduction of PUFA synthesis in CKD macrophages with a shunting of saturated FFA towards glycerophospholipid metabolism. Moreover, IPA network analysis showed that differentially expressed lipids were closely related to activation of multiple known regulators of intracellular lipid metabolism including peroxisome proliferator-activated receptor gamma, sterol-regulatory element binding proteins, and long-chain ACSL (Fig. 4C). The complete regulatory network is shown in Supplemental Figure S24.

To explore potential drivers for these observed changes in CKD PMΦ lipid expression, we also conducted upstream regulator analysis using IPA. Four statistically significant upstream regulators were identified from the panel of differentially expressed lipids (Fig. 4D–E). The single gene with an activation Z score of >2.0 was long-chain ACSL1. The ACSL1 gene is one member of the long-chain ACSL family of enzymes required for esterification of FFA to their acyl-CoA derivatives and is the most abundant ACSL expressed in macrophages. In addition, upstream regulator analysis also identified fatty acid transport protein 4 (FATP4), elongation factor Tu GTP binding domain containing 2, and the vitamin D receptor as potentially inhibited regulators with Z-scores <−2.0. Notably, FATP4 also has acyl-CoA synthetase function for long-chain fatty acids, and is predominately expressed in the gastrointestinal tract, gonads, brain, and macrophages. However, deficiency of FATP4, is associated with attenuated inflammatory responses and decreased oxidative stress in macrophages (22).

### CKD increases macrophage ACSL1 and inflammatory mediators

**Fig. 5:**
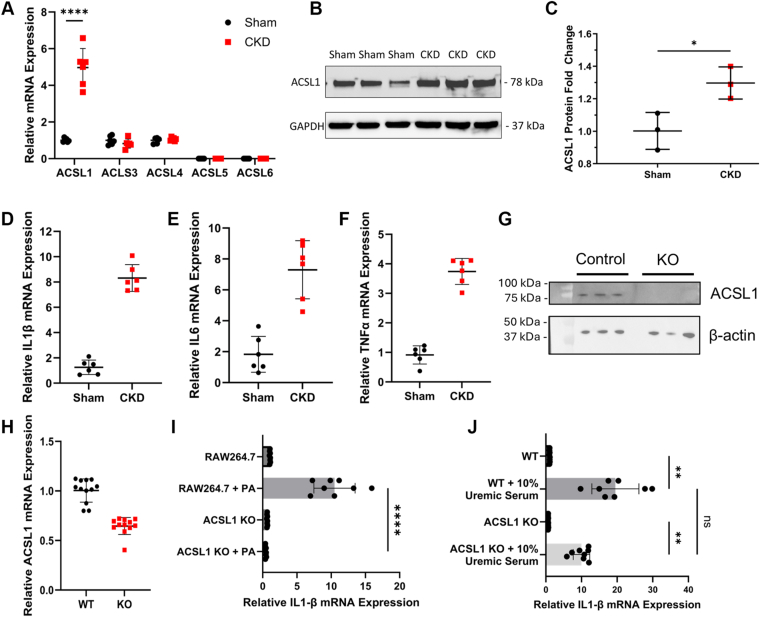
CKD induces macrophage expression of Acsl1 and inflammatory mediators. A: Mouse peritoneal macrophages (PMΦ) were isolated from CKD and control (sham) mice, and *Acsl1*, *Acsl3*, *Acsl4*, *Acsl5*, and *Acsl6* mRNA levels were measured by real-time PCR (n = 6/group). B, C: Immunoblot and relative quantification for Acsl1 and Gapdh protein from sham and CKD PMΦ (n = 5/group). C–F: Relative mRNA expression of inflammatory mediators Il-1β, Il-6, and Tnf-α from sham and CKD PMΦ by real-time PCR (n = 6/group). G–J: Knockdown of Acsl1 was generated in the RAW264.7 cell line by CRISPR/Cas9. G: Immunoblot of Acsl1 protein expression in KO RAW264.7 cells and controls relative to β-actin. H: Acsl1 mRNA expression in wildtype (WT) and KO RAW264.7 cells by real-time PCR (n = 10/group). I, J: WT and Acsl1 KO macrophages were stimulated with (I) 200 mM palmitic acid-BSA or (J) 10% uremic mouse serum and IL1-β mRNA expression measured by real time PCR. Unconjugated BSA or 10% mouse serum was used as controls, respectively. Statistical differences in protein and mRNA expression levels were assessed using a two-tailed unpaired student’s *t* test. Data are shown as mean ± 95% confidence interval. ∗*P* < 0.05, ∗∗*P* < 0.01, and ∗∗∗∗*P* < 0.001. ACSL1, long chain acyl-CoA synthetase 1; IL-1β, interleukin 1 beta; IL-6, interleukin 6; TNF-α, tumor necrosis factor alpha; CKD, chronic kidney disease; PMΦ, peritoneal macrophages.

To further investigate the potential role of ACSLs in CKD macrophage lipid metabolism and inflammation, we assessed the expression of *Acsl1*, *Acsl3*, *Acsl4*, *Acsl5*, *Acsl6*, and multiple inflammatory markers in PMΦ collected from sham and CKD mice. CKD significantly increased PMΦ *Acsl1* expression by 5-fold compared to sham PMΦ without compensatory changes in the expression of other ACSLs (Fig. 5A). This was associated with a modest increase in Acsl1 protein expression compared to control PMΦ (Fig. 5B–C) and increased mRNA expression of inflammatory cytokines interleukin-1 β, interleukin-6, and tumor necrosis factor alpha (Fig. 5D–F).

To further examine the role of ACSL1 in CKD lipid metabolism and inflammation, we generated a RAW264.7 cell line with the specific deletion of Acsl1 (Acsl1KO) using CRISPR/Cas9 genome editing. After confirming a significant decrease in Acsl1 protein and mRNA levels in the Acsl1KO cells (Fig. 5G–H), Acsl1KO cells and control RAW264.7 cells were treated with either palmitate (PA) or 10% pooled uremic mouse serum for 24 h. PA treatment significantly increased IL-1β mRNA expression in RAW264.7 cells, and this response was abolished in Acsl1KO cells (Fig. 5I). Similarly, stimulation with uremic serum significantly induced IL-1β mRNA expression in RAW264.7 cells, while this response was attenuated in Acsl1KO cells (Fig. 5J).

## Discussion

Macrophages reprogram their intracellular lipid metabolism in response to the activation of cytokine and pattern recognition receptors (Toll-like receptors, TLRs) to achieve their ultimate immunological function. Prior research over the past decade has begun to elucidate how distinct pro-inflammatory stimuli reshape the macrophage lipidome in a signal-specific manner (23). However, despite this understanding, macrophages are subject to a plethora of stimuli in vivo that often do not fully fit into the binary M1/M2 framework (24). CKD represents one example of this complex in vivo milieu and is characterized by dyslipidemia and the accumulation of uremic metabolites, which potentiate chronic inflammation (6, 25). While prior research has extensively characterized plasma lipidomic alterations in CKD, understanding how CKD affects the macrophage lipidome remains limited.

This study highlights significant alterations in the macrophage lipidome of mice with moderate CKD. Key changes include a higher abundance of saturated FFA, PE, PG, saturated PE-P lipids, and modified ceramides alongside a lower abundance of unsaturated FFA, TAG, DAG, and PC lipids. Importantly, this profile was distinct from alterations seen in the plasma of CKD mice. Pathway analysis identified ACSL1 as a potential upstream driver of these observed changes in macrophage lipid metabolism. Consistent with this finding, we detected increased *Acsl1* expression in macrophages from CKD mice, accompanied by elevated levels of several inflammatory cytokines. Together, these findings provide novel insights into possible mechanisms by which CKD alters macrophage lipid metabolism and function, potentially contributing to the heightened inflammatory state and increased risk of ASCVD in CKD.

The glycerophospholipid signature we observed in CKD mice complements and extends previous studies examining plasma lipidomic profiles in CKD. We observed increased levels of saturated FFA and modified ceramide lipid species coupled with a lower abundance of PC, lysophosphatidylcholine, and SM species, similar to several prior studies from the plasma of patients with progressive CKD(3,11). Interestingly, while our tissue-specific analysis revealed increased levels of PE and saturated ether-linked PE-O and PE-P species in CKD macrophages and plasma, plasma studies in CKD patients have generally reported decreased circulating levels of PE and PE-P lipid species (10). This discrepancy suggests that metabolic dysfunction in CKD is both systemic and cell-specific, underscoring the need to examine lipid metabolism at both the systemic and cellular levels. A notable observation from our study is the differential expression and correlation of saturated and unsaturated fatty acids in CKD macrophages. We found that CKD promoted the macrophage-specific accumulation of saturated FFA, with reduced levels of unsaturated FFA and glycerolipid species. Notably, this pattern was discordant from that observed in the plasma, and suggests that CKD rewires macrophage lipid metabolism, favoring saturated FFA incorporation into glycerophospholipids, particularly PE species over neutral lipids and PC. One plausible explanation for these shifts is the dysregulation of enzymatic pathways that govern fatty acid desaturation in CKD, such as stearoyl-CoA desaturase. Our partial correlation analysis supports this model by revealing that macrophages in CKD preferentially channel unsaturated FFA into DAGs and TAGs. In contrast, saturated FFA are increasingly incorporated into membrane phospholipids, particularly PE species, which may compromise membrane fluidity and contribute to the pro-inflammatory phenotype typical of CKD macrophages (26).

The macrophage-specific lipidomic alterations we observed must also be considered within the broader context of systemic metabolic dysregulation in CKD. Patients with CKD exhibit multiple metabolic abnormalities, including insulin resistance, dyslipidemia, and oxidative stress, all of which can influence mitochondrial function and macrophage lipid handling (27). Even subtle perturbations in kidney function have been shown to impair macrophage cholesterol efflux after uni-nephrectomy in mice through downregulation of cholesterol efflux transporters ABCA1(8). Additionally, the accumulation of advanced glycation end products in diabetes or CKD can hinder cholesterol efflux through the receptor for advanced glycation end products (28). Finally, CKD and comorbid diabetes are both associated with increased levels of circulating ceramides and ASCVD events (29). While we did not observe significant elevations in macrophage ceramide species, we did note elevated levels of hexosylceramide and monounsaturated lactosylceramide in CKD PMΦ. Prior studies have shown that elevated levels of modified ceramide lipid species may potentiate chronic inflammation in CKD, partially through modulation of NFκB signaling (30). Together, these studies suggest that multiple aspects of lipid metabolism are dysregulated in CKD macrophages, collectively contributing to a pro-inflammatory, pro-atherogenic phenotype.

A particularly notable finding was the increased levels of saturated PE-O (plasmanyl) and PE-P (plasmenyl) phospholipids in CKD macrophages. These ether-linked phospholipids represent specialized membrane components that play crucial roles in cellular signaling, membrane stability, and oxidative stress responses. PE-P species, also known as plasmalogens, contain a vinyl ether bond at the sn-1 position and are particularly enriched in PUFAs at the sn-2 position, making them important reservoirs of arachidonic acid (31, 32). In addition, plasmalogens serve as endogenous antioxidants and markers of oxidative stress, protecting cellular membranes from oxidative damage through their vinyl ether bonds, which act as sacrificial targets for reactive oxygen species (33). In the context of CKD, where oxidative stress is elevated, the accumulation of PE-P species in macrophages may represent a compensatory mechanism to maintain membrane integrity and cellular function. Interestingly, prior studies by our group have noted an association between increasing systemic levels of PE and PE-P phospholipids with new ischemic stroke in CKD (34). However, further studies are required to assess whether the macrophage lipid reservoir contributes to this elevation and increased stroke risk. Given that plasmalogen biosynthesis begins with the esterification of acyl-CoA and dihydroxyacetone phosphate (DHAP) instead of DAG for other glycerophospholipids, it is likely that alterations in upstream regulators of lipid metabolism play an important role in macrophage immunometabolism in CKD.

To investigate alterations in lipid metabolic pathways that could explain the observed differences between the macrophage lipidome and the systemic lipidome in CKD, we performed pathway analysis. Our findings identified ACSL1 as a potential upstream regulator of the altered lipid metabolism in CKD macrophages. ACSL1 belongs to the long-chain acyl-CoA synthetase family and catalyzes the conversion of long-chain fatty acids to acyl-CoA, serving as a critical regulatory step in both fatty acid oxidation and complex lipid synthesis pathways (35). ACSL1 is the most abundant acyl-CoA synthetase expressed in macrophages, and previous studies support the concept that acylation of FFA by ACSL1 is a driver of inflammation in multiple metabolic disorders (36). In the context of diabetes, a disease also characterized by dyslipidemia and mitochondrial dysfunction, myeloid-specific deletion of *Acsl1* has been shown to inhibit the inflammatory activation of macrophages and prevent diabetes-accelerated atherosclerosis in mice (37). The concurrent increases in *Acsl1* expression and inflammatory cytokine production we observed in CKD macrophages suggest a similar relationship between the altered macrophage lipidome and inflammation in CKD. Interestingly, recent studies by Barnhart *et al.* (38), demonstrated that type I interferon–driven *Acsl1* induction in macrophages drives formation of saturated phosphatidic acid pools, protecting against lipotoxicity and reducing necrotic core formation in inflamed atherosclerotic lesions. These findings support the concept that in our CKD model, inflammation and elevated *Acsl1* may channel excess saturated FAs into membrane phospholipids rather than neutral lipid droplets, altering membrane lipid composition, fluidity, and signaling capacity. Such remodeling could promote atherogenesis by potentiating pro-inflammatory activation, impairing cholesterol efflux, or increasing foam-cell vulnerability. Consistently, we also observed suppressed de novo lipogenesis in CKD macrophages with elevated saturated FFA levels, supporting exogenous fatty acid uptake as the primary source of intracellular FFA. Further mechanistic studies exploring the causal relationships between ACSL1 upregulation, altered glycerophospholipid profiles, and inflammatory cytokine production would further clarify the pathways linking lipid metabolism to inflammation in CKD.

While our study offers valuable insights into macrophage lipid metabolism in CKD, several limitations should be acknowledged. Our analysis was conducted using a mouse model of CKD, and the extent to which these findings can be applied to human disease remains to be determined. Additionally, our study focused on elicited peritoneal macrophages, rather than specific subsets or atherosclerotic lesion foam cells. It is possible that foam cell lipid metabolism and phenotype from within atherosclerotic lesions may differ, but lipidomic analyses of atherosclerotic plaque foam cells have been limited to date due to the need for sufficient cell quantity and purity. A recent study by Seo *et al.* used 39 LDLr^−/−^ mice to isolate sufficient aortic macrophages for a single lipidomics sample with limited statistical power (39). Furthermore, macrophage lipid composition also varies by cellular activation state (23). While employing bone marrow-derived macrophages would enable the analysis of different macrophage phenotypes after stimulation in vitro, the prolonged differentiation period ex vivo is likely to diminish or negate the effect of CKD. Finally, while our study included a pathophysiological model of cardiovascular-kidney-metabolic syndrome, it was restricted to only one mouse strain and the lipid metabolic response to CKD could be different between strains.

In summary, our lipidomic analysis of macrophages from mice with CKD has revealed significant alterations in glycerophospholipid composition, associated with increased *Acsl1* expression, reduced fatty acid oxidation, and enhanced inflammatory cytokine production. These findings extend previous observations of dysregulated lipid metabolism in CKD from the circulation to immune cells and identify ACSL1 as a potential molecular link between altered lipid metabolism and inflammation in kidney disease. Further investigation of these pathways may yield novel therapeutic approaches for addressing the persistent inflammation that contributes to morbidity and mortality in CKD patients.

## Data Availability

This study is available at the NIH Common Fund's National Metabolomics Data Repository (NMDR) website, the Metabolomics Workbench, https://www.metabolomicsworkbench.org where it has been assigned Study ID ST004519. The data can be accessed directly via its Project : http://dx.doi.org/10.21228/M8J276.

## Supplemental Data

This article contains supplemental data.

## Conflict of Interest

The authors declare that they have no conflicts of interest with the contents of this article.
