# Supplementary material for: Chronic kidney disease induces distinct alterations of macrophage lipid metabolism in a mouse model of atherosclerosis
Source: J Lipid Res. 2026 Jan 2;67(2):100975. doi: 10.1016/j.jlr.2026.100975 (PMC12860704; doi:10.1016/j.jlr.2026.100975)

Supplemental Materials

Table of Contents

[Figure S1: Flow Cytometry Analysis of Peritoneal Cell Isolates 2](#_Toc215565687)

[Figure S2: macrophage free fatty acids (FFA) from control and CKD mice 3](#_Toc215565688)

[Figure S3: macrophage triacyglycerols (TAG) from control and CKD mice 4](#_Toc215565689)

[Figure S4: macrophage diacylglycerols (DAG) from control and CKD mice 5](#_Toc215565690)

[Figure S5: macrophage monoacylglycerols (MAG) from control and CKD mice 6](#_Toc215565691)

[Figure S6: macrophage cholesterol esters (CE) from control and CKD mice 7](#_Toc215565692)

[Figure S7: macrophage phosphatidylcholines (PC) from control and CKD mice 8](#_Toc215565693)

[Figure S8: macrophage lysophosphatidylcholines (LPC) from control and CKD micE 9](#_Toc215565694)

[Figure S9: macrophage phosphatidylethanolamines (PE) from control and CKD mice 10](#_Toc215565695)

[Figure S10: macrophage lysophosphatidylethanolamines (LPE) from control and CKD mice 11](#_Toc215565696)

[Figure S11: macrophage PE-O from control (sham) and CKD mice 12](#_Toc215565697)

[Figure S12: macrophage PE-P from control (sham) and CKD mice 13](#_Toc215565698)

[Figure S13: macrophage phosphatidylglycerols (PG) from control and CKD mice 14](#_Toc215565699)

[Figure S14: macrophage phosphatidylinositols (PI) from control and CKD mice 15](#_Toc215565700)

[Figure S15: macrophage phosphatidylserines (PS) from control and CKD mice 16](#_Toc215565701)

[Figure S16: Macrophage lysophosphatidylserines (LPS) from control and CKD micE 17](#_Toc215565702)

[Figure S17: macrophage ceramides (CER) from control and CKD mice 18](#_Toc215565703)

[Figure S18: macrophage dihydroceramides (DCER) from control and CKD mice 19](#_Toc215565704)

[Figure S19: macrophage hexosylceramides (HCER) from control and CKD mice 20](#_Toc215565705)

[Figure S20: macrophage lactosylceramides (LCER) from control and CKD mice 21](#_Toc215565706)

[Figure S21: macrophage sphingomyelins (SM) from control and CKD mice 22](#_Toc215565707)

[Figure S22: Correlation of saturated FFA levels and glycerolipids by class in sham and CKD macrophages. 23](#_Toc215565708)

[Figure S23: Unsaturated FFA lipid correlation networks in CKD and sham PMΦ 24](#_Toc215565709)

[Figure S24: Predicted upregulation of multiple regulators of lipid metabolism in CKD peritoneal macrophages. 25](#_Toc215565710)

[Table S1: List of quantified lipids 26](#_Toc215565711)

[Table S2: Differentially Altered Lipids in CKD Macrophages and Plasma 47](#_Toc215565712)

# **Figure S1:** Flow Cytometry Analysis of Peritoneal Cell Isolates

**
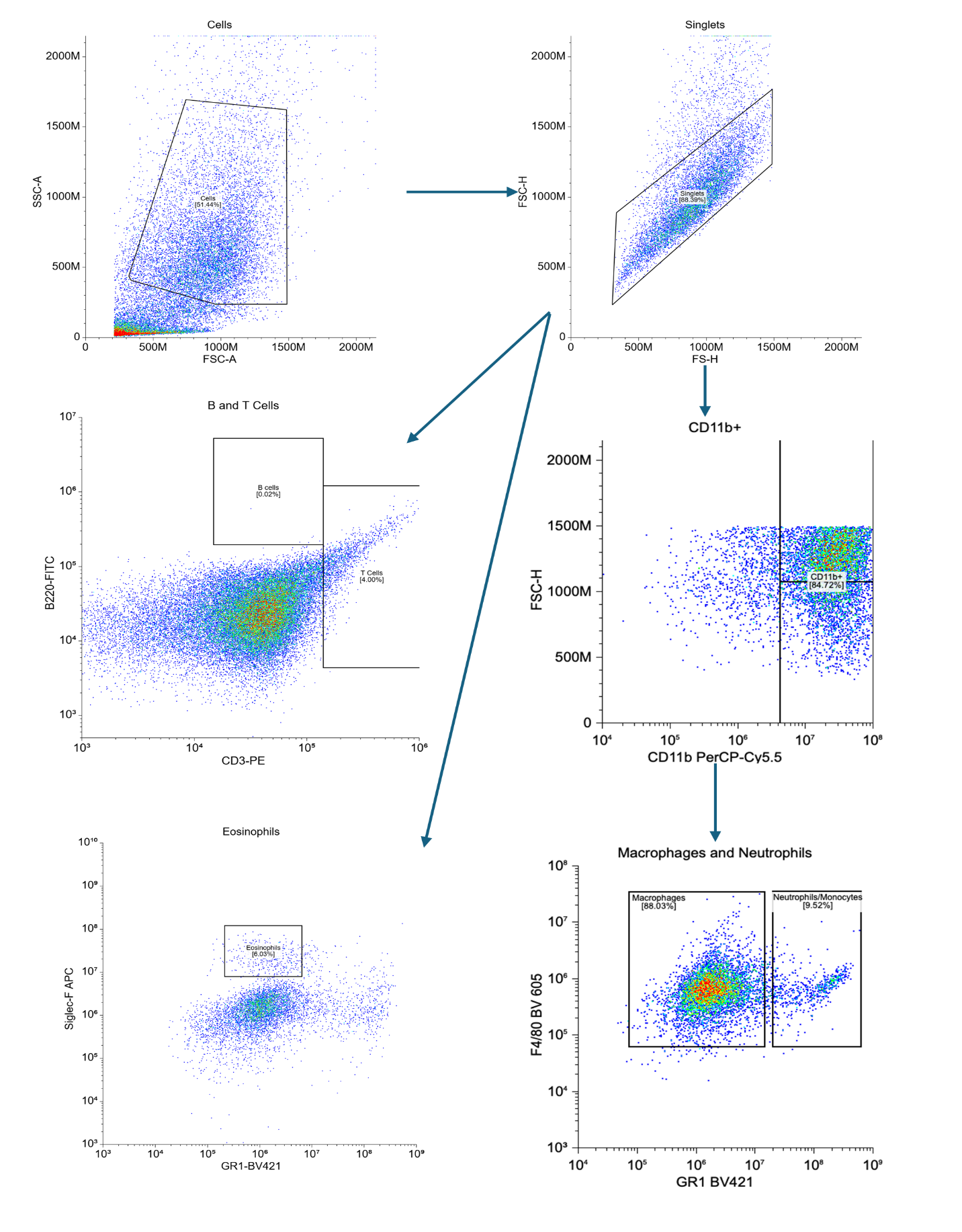
**

# **Figure S2:** Standardized mean levels of peritoneal macrophage free fatty acids (FFA) from control (sham) and CKD mice after 16 weeks of high fat diet


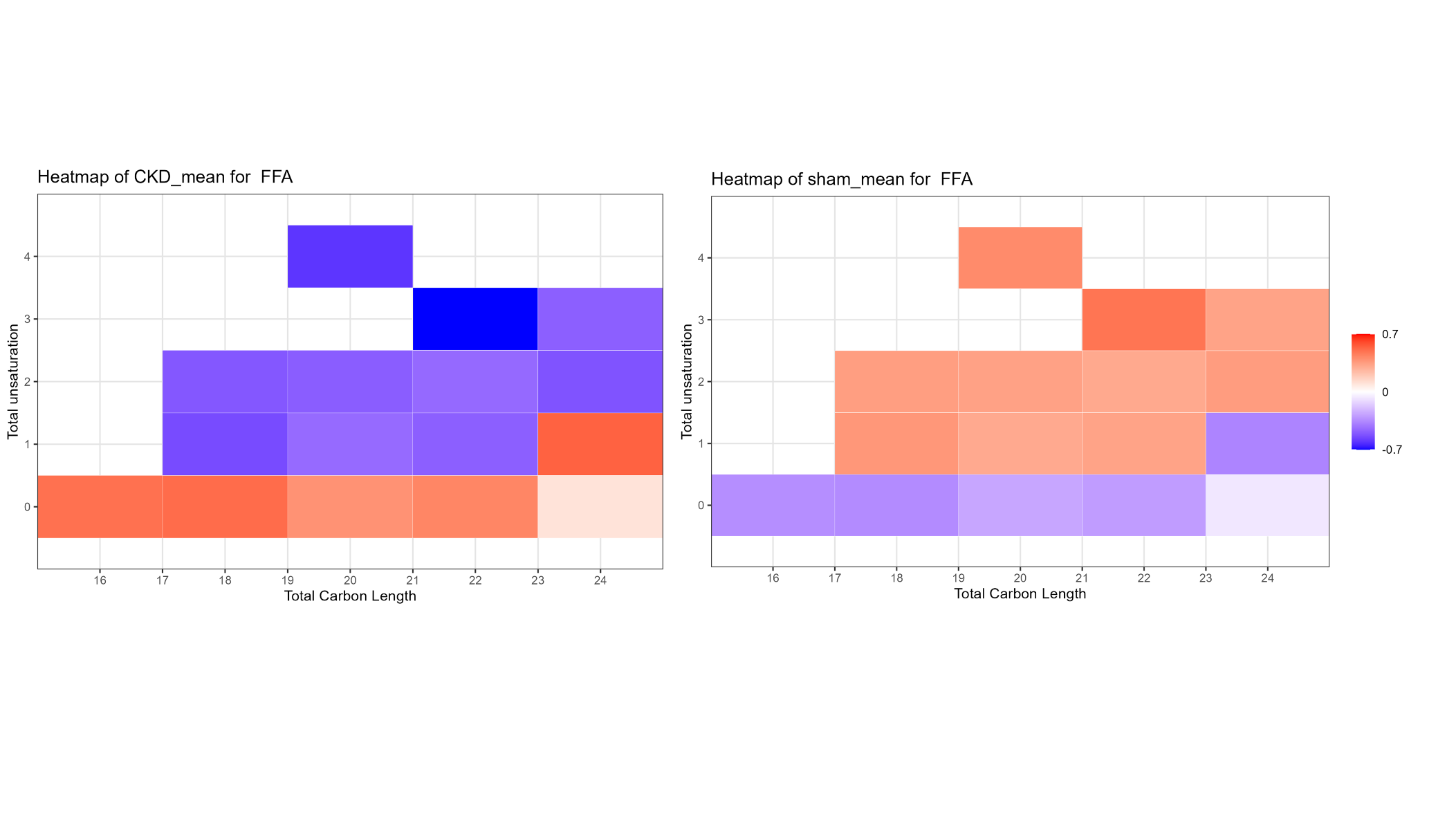

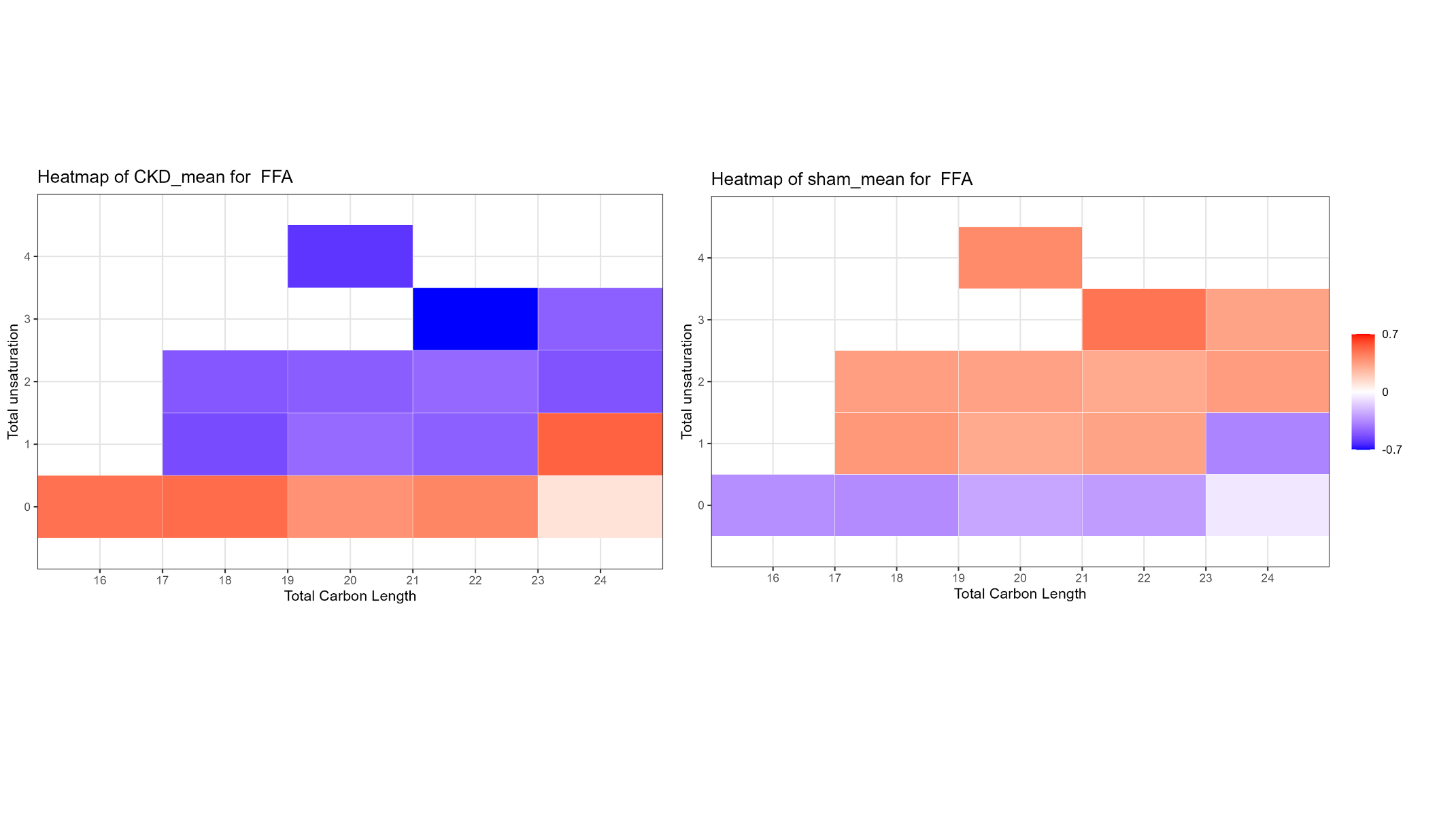


# **Figure S3:** Standardized mean levels of peritoneal macrophage triacyglycerols (TAG) from control (sham) and CKD mice after 16 weeks of high fat diet


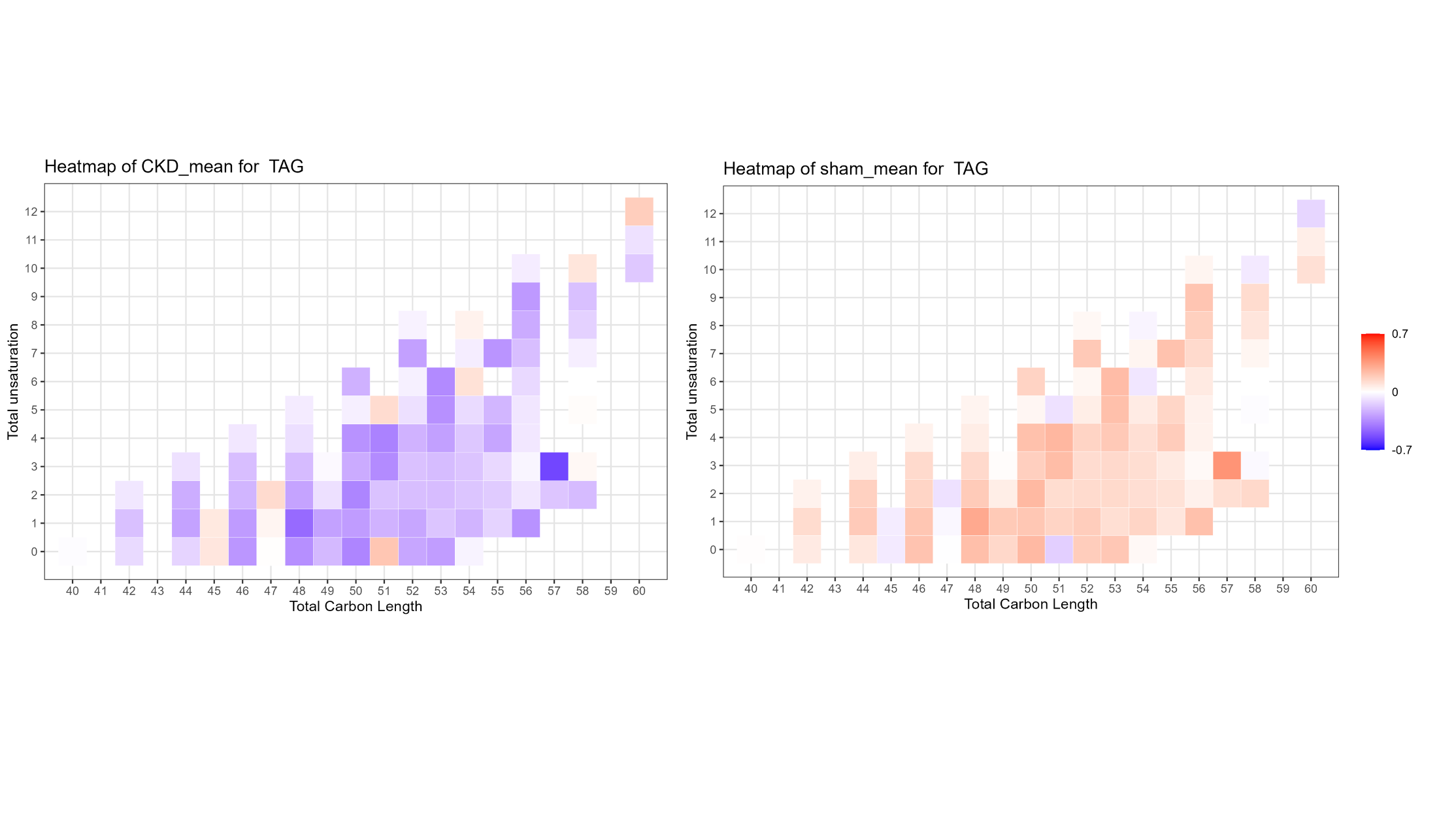


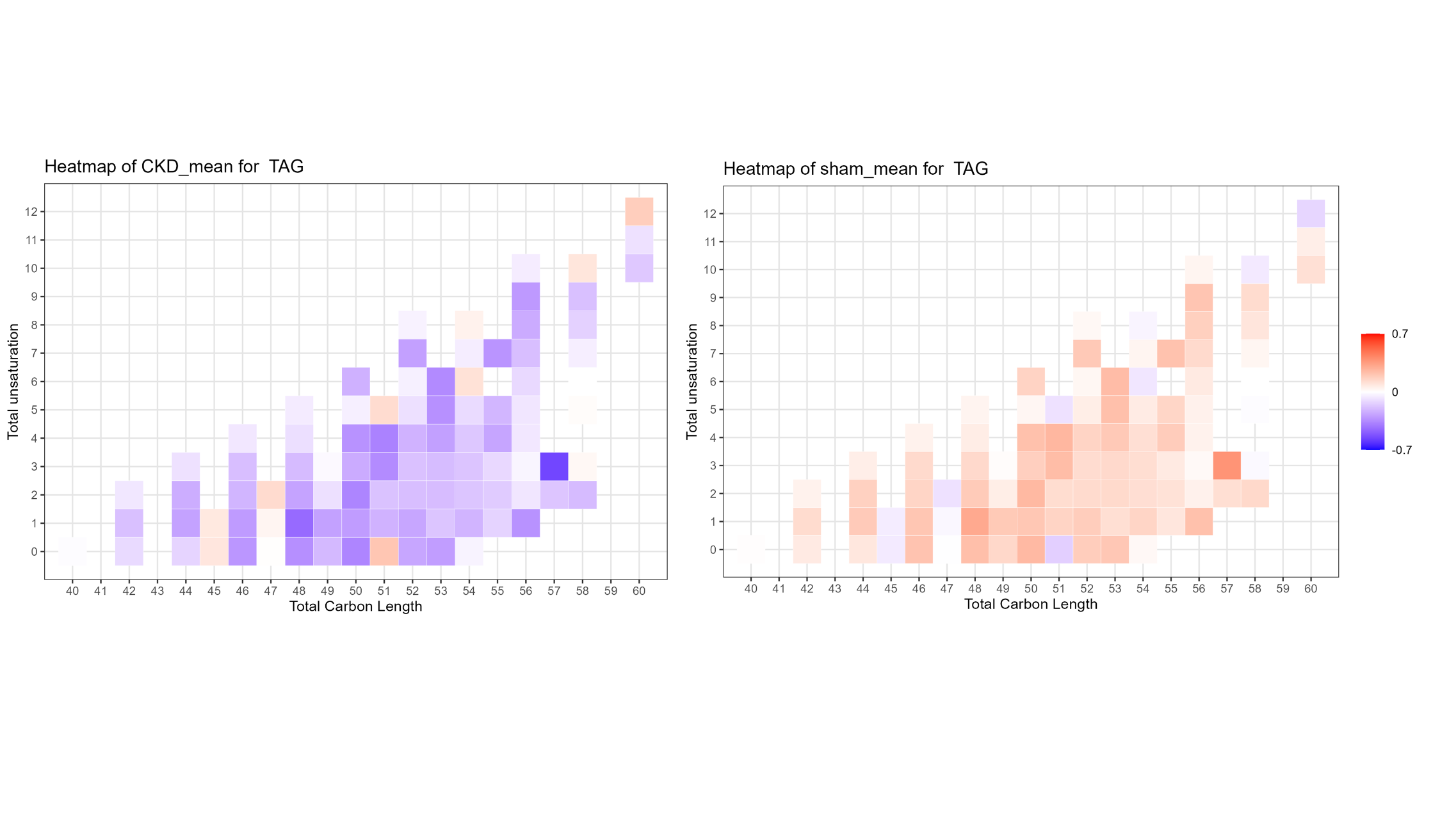


# **Figure S4:** Standardized mean levels of peritoneal macrophage diacylglycerols (DAG) from control (sham) and CKD mice after 16 weeks of high fat diet


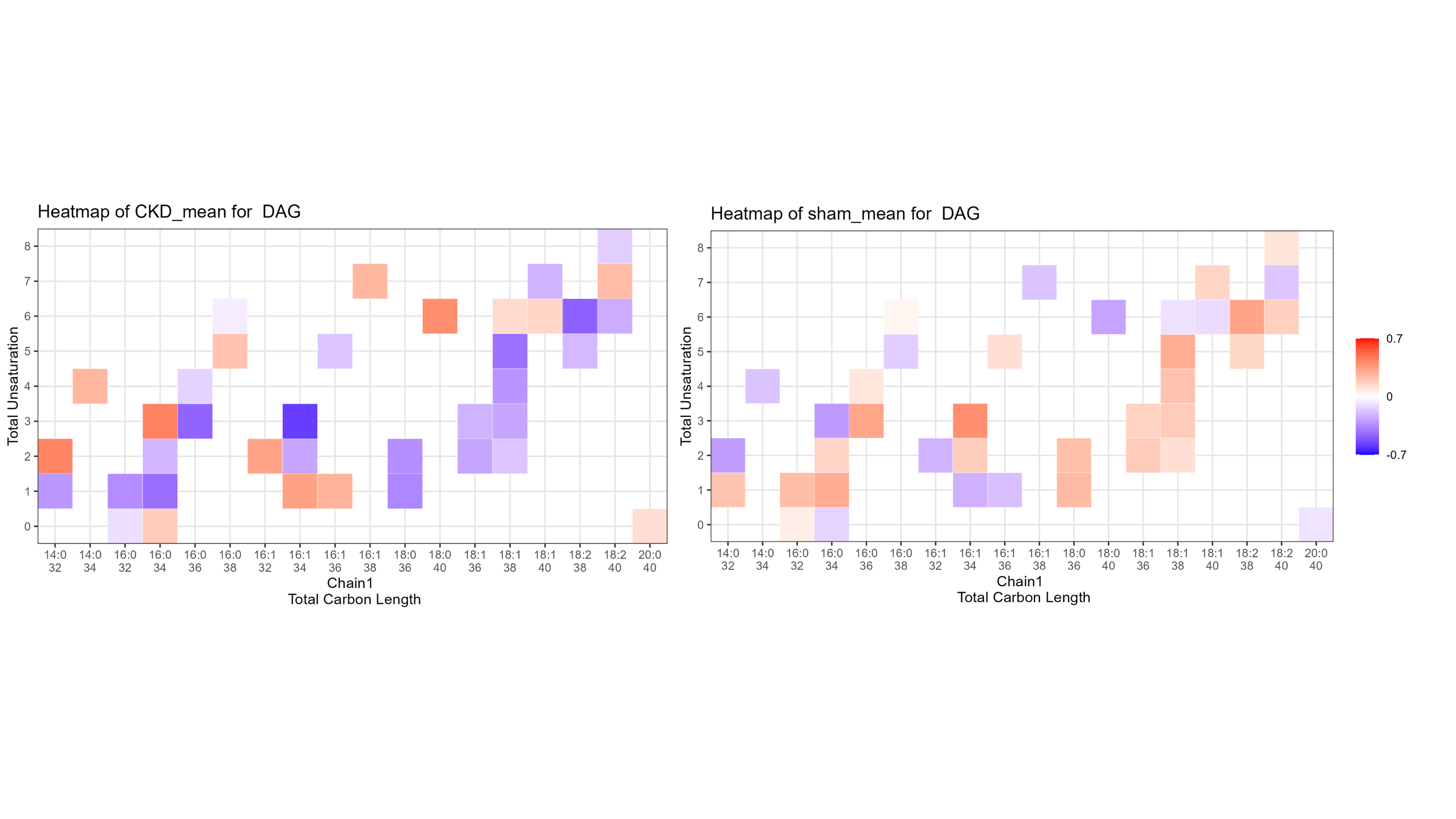


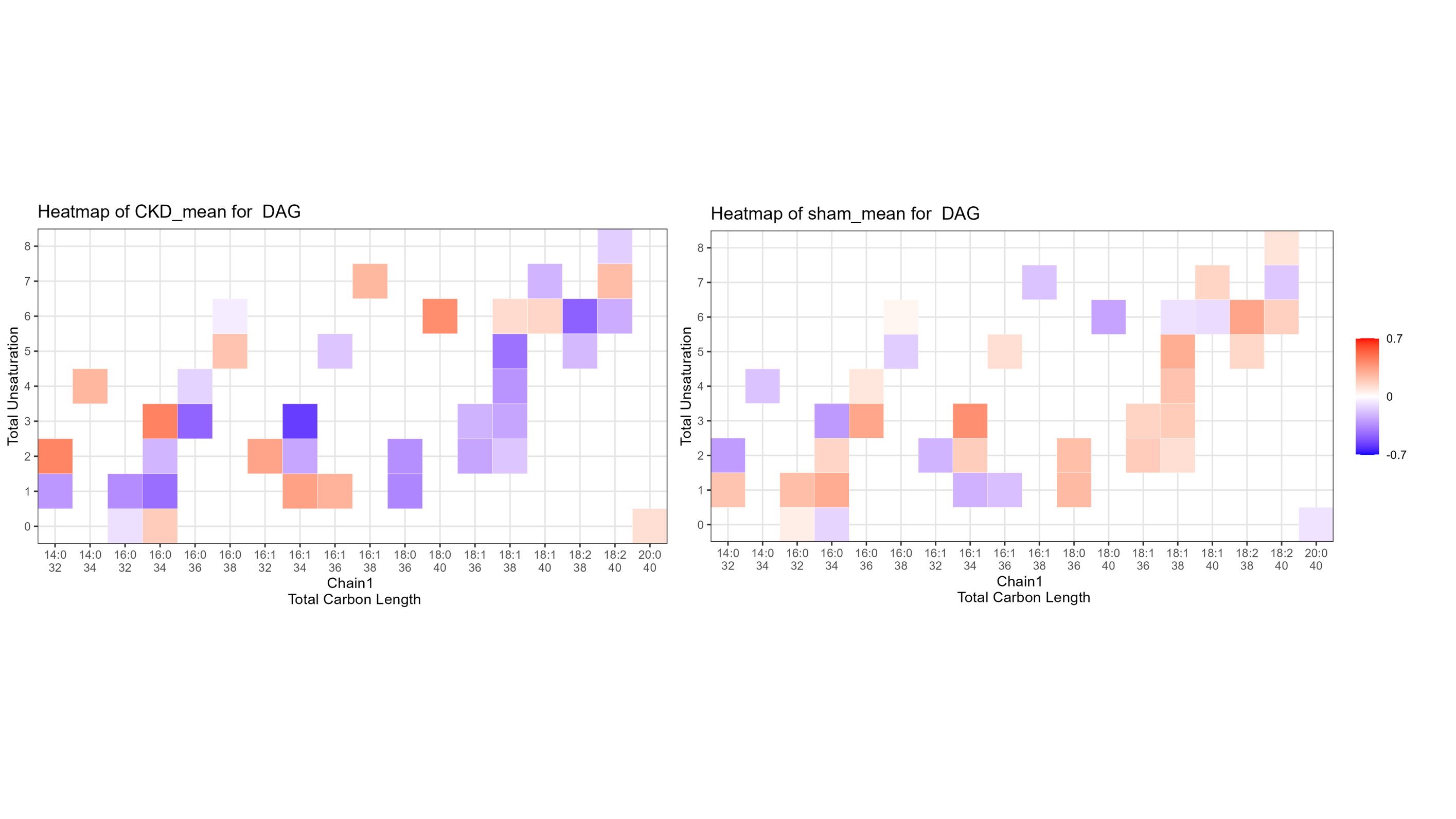


# Figure S5: Standardized mean levels of peritoneal macrophage monoacylglycerols (MAG) from control (sham) and CKD mice after 16 weeks of high fat diet


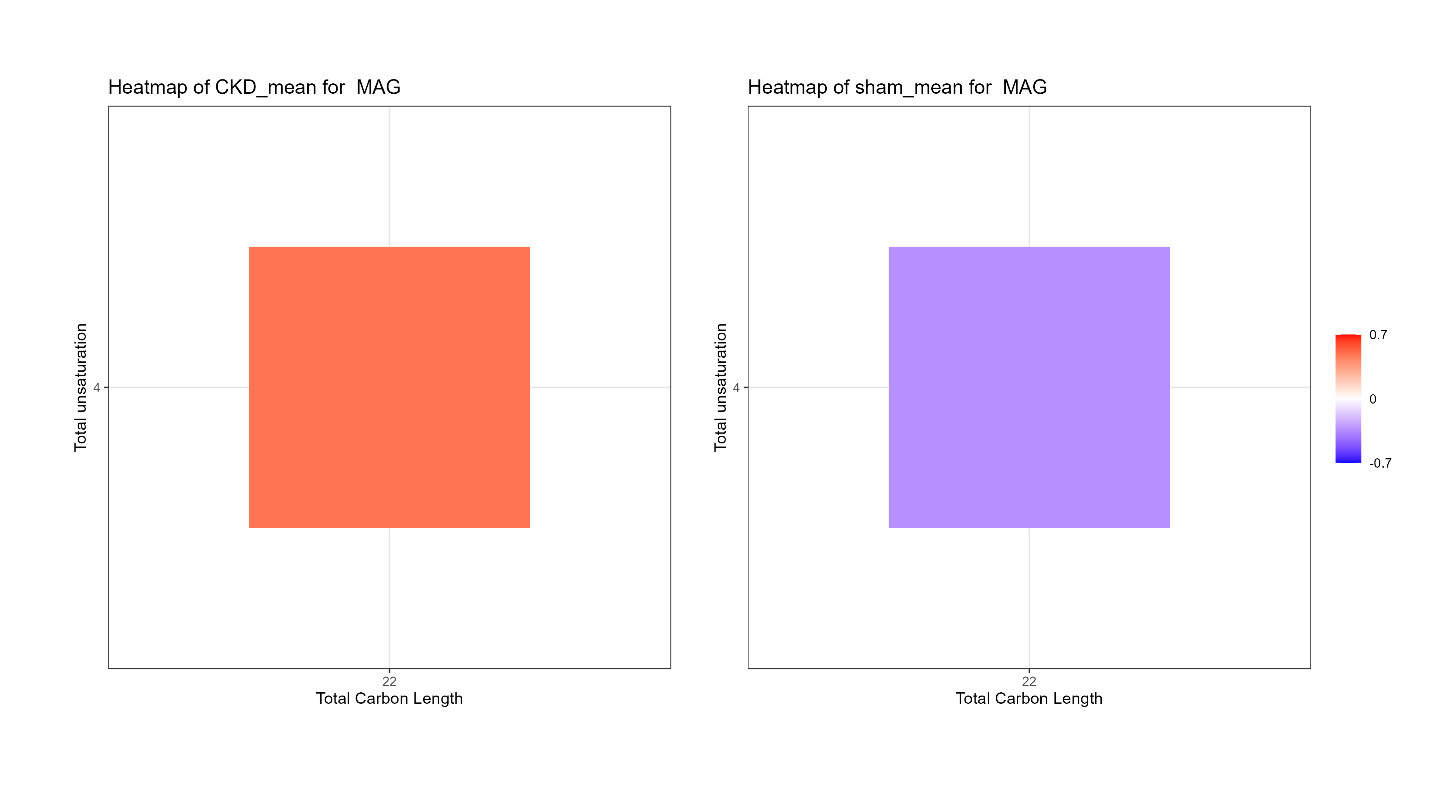


# **Figure S6:** Standardized mean levels of peritoneal macrophage cholesterol esters (CE) from control (sham) and CKD mice after 16 weeks of high fat diet

**
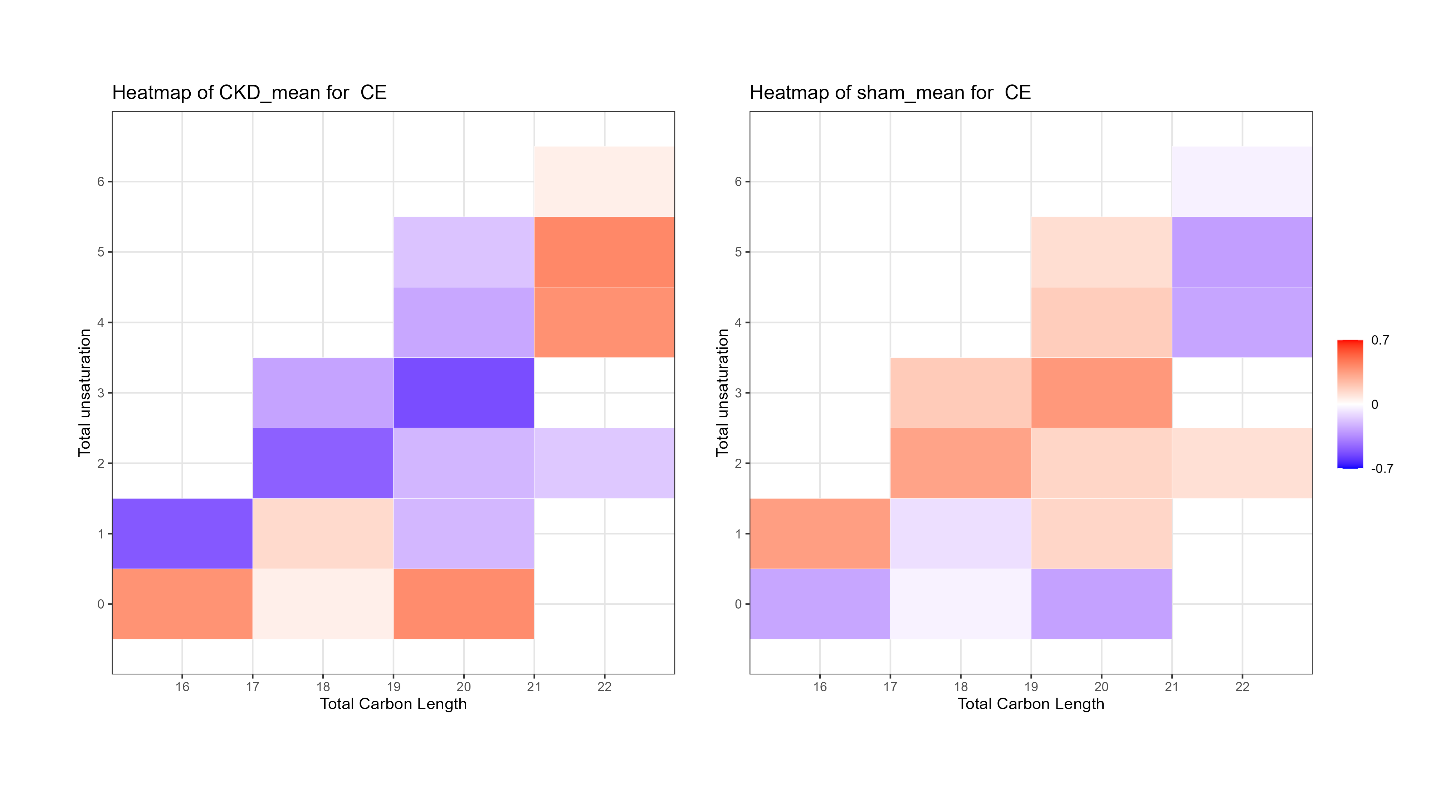
**

# **Figure S7:** Standardized mean levels of peritoneal macrophage phosphatidylcholines (PC) from control (sham) and CKD mice after 16 weeks of high fat diet

**
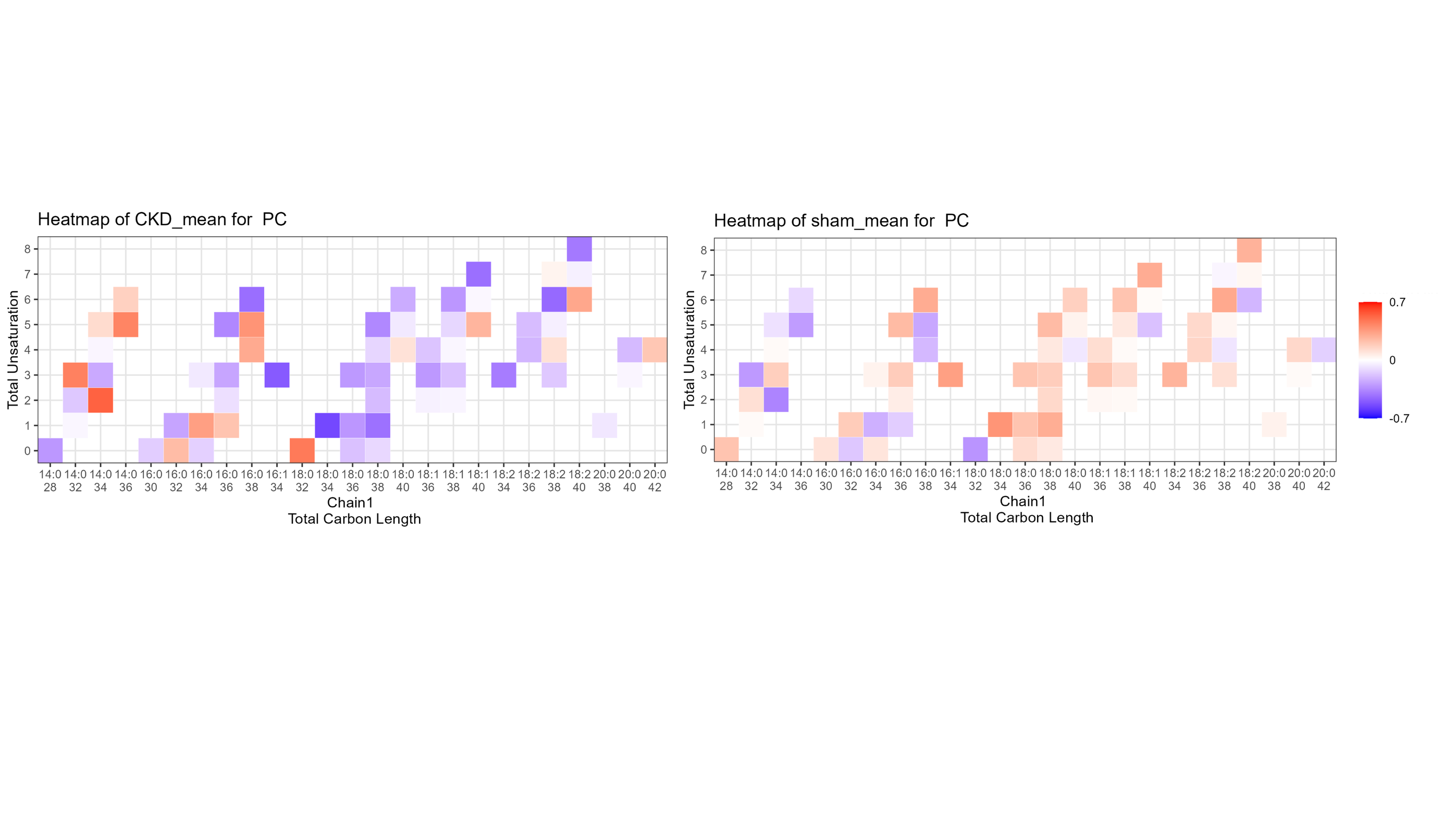
**

**
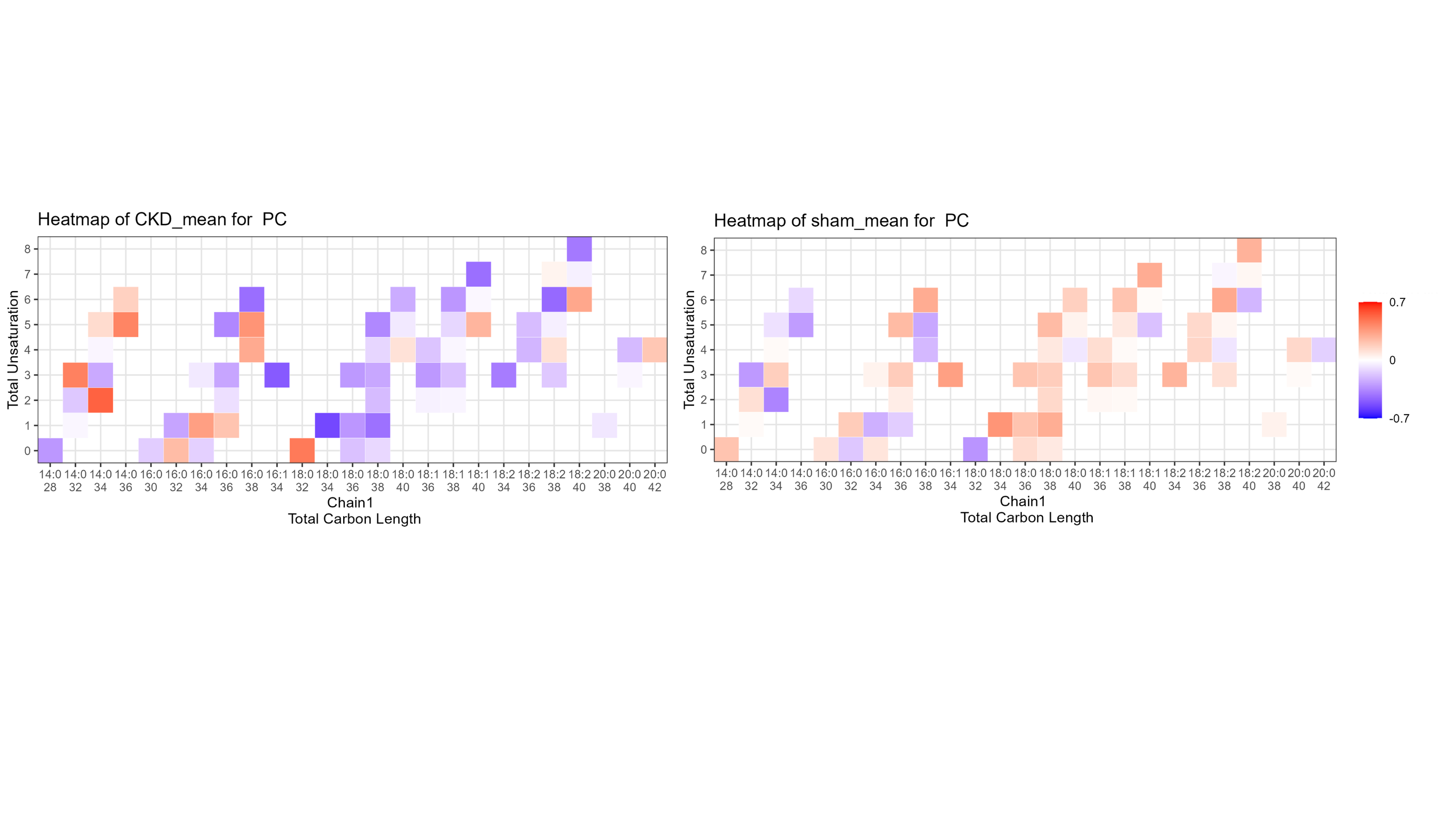
**

# **Figure S8:** Standardized mean levels of peritoneal macrophage lysophosphatidylcholines (LPC) from control (sham) and CKD mice after 16 weeks of high fat diet

**
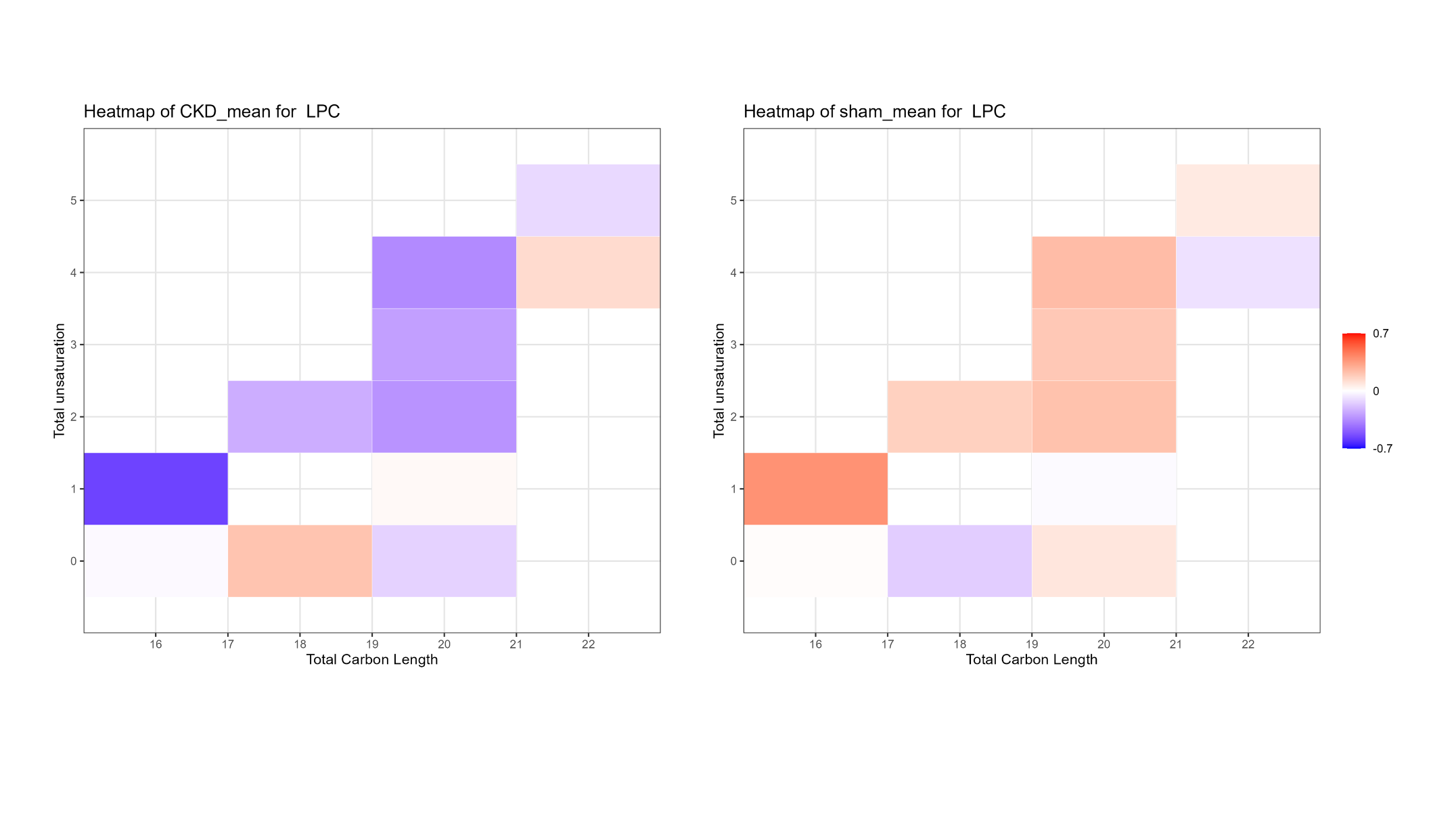
**

**
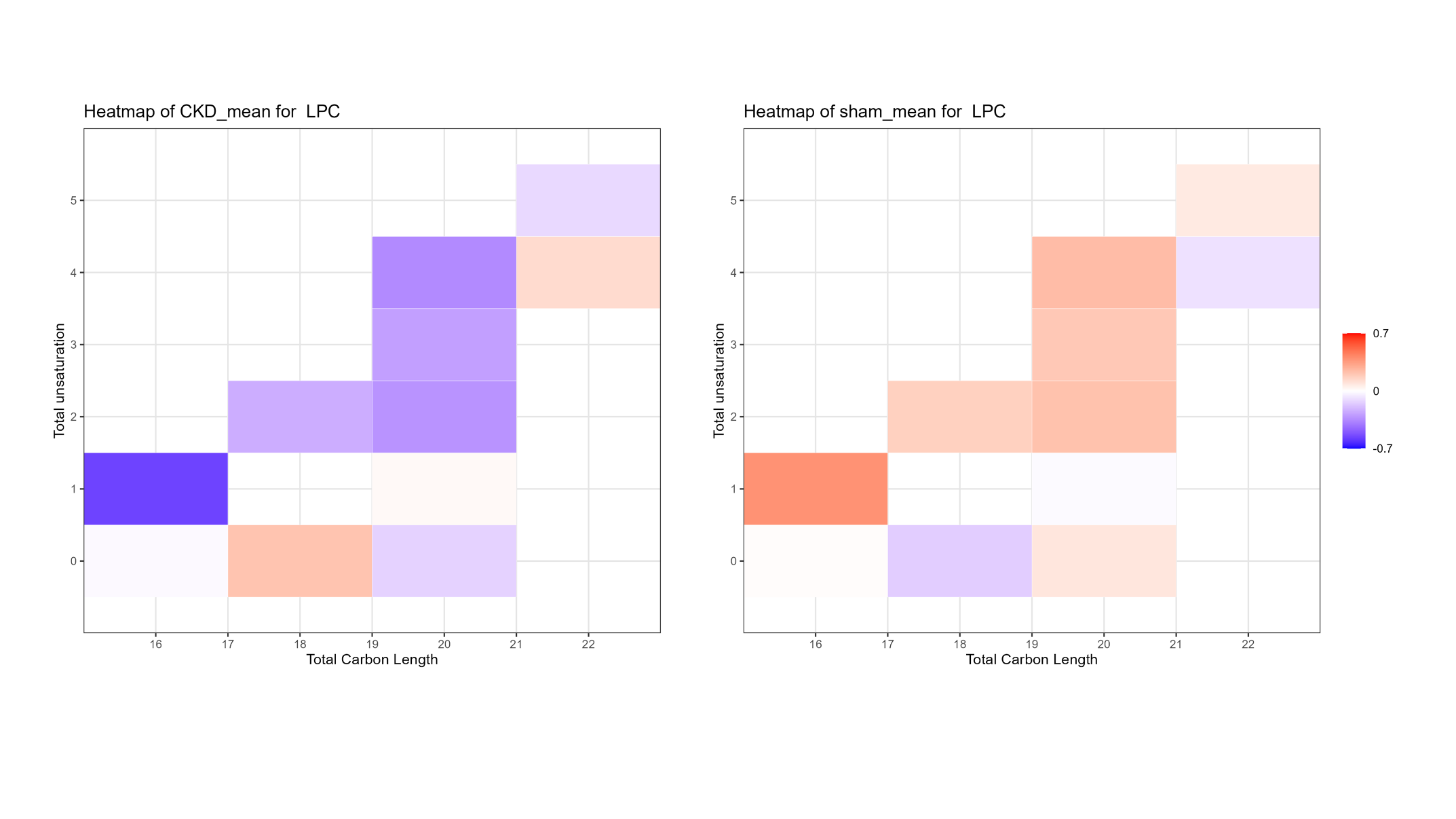
**

# **Figure S9:** Standardized mean levels of peritoneal macrophage phosphatidylethanolamines (PE) from control (sham) and CKD mice after 16 weeks of high fat diet

**
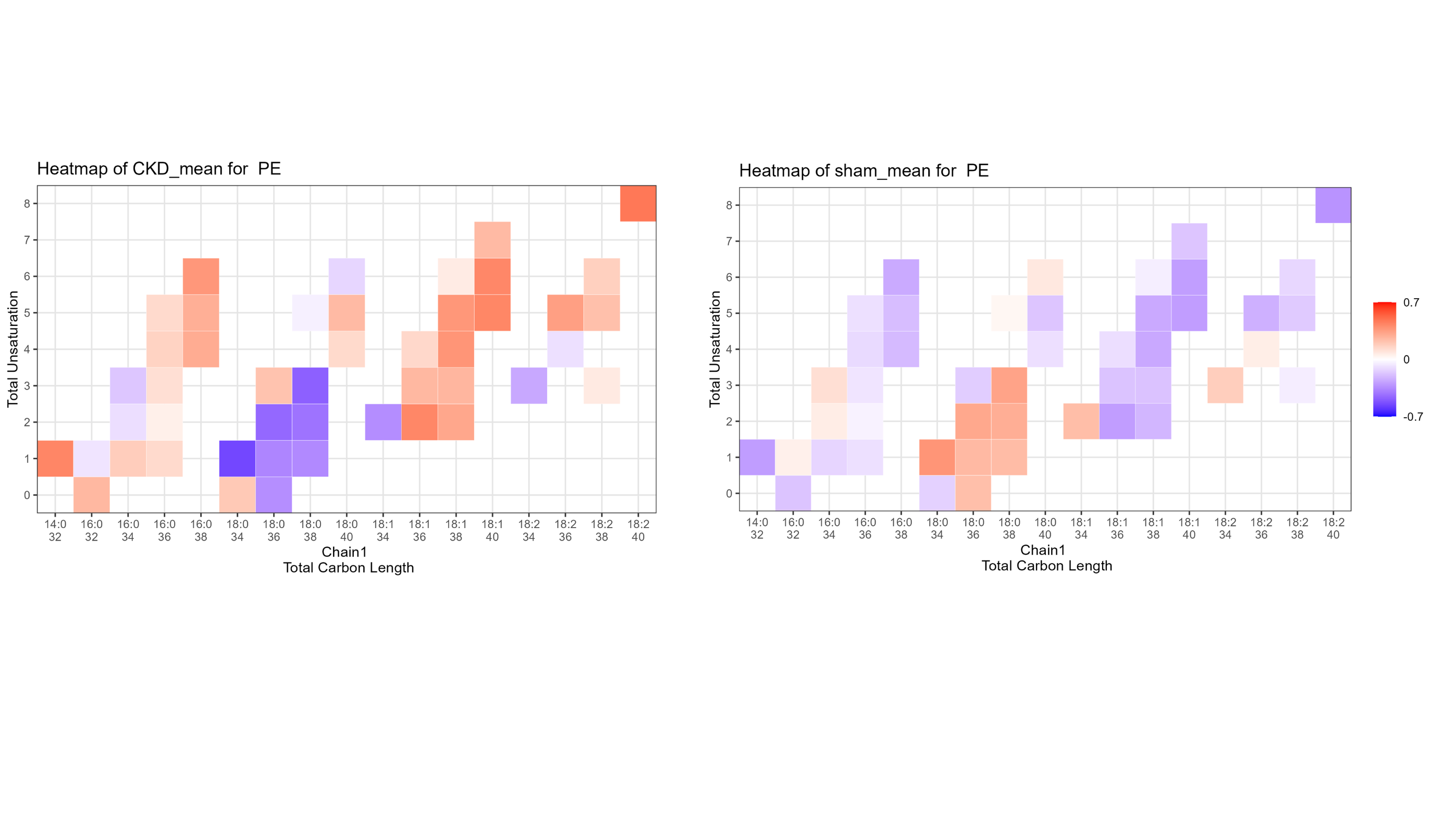
**

**
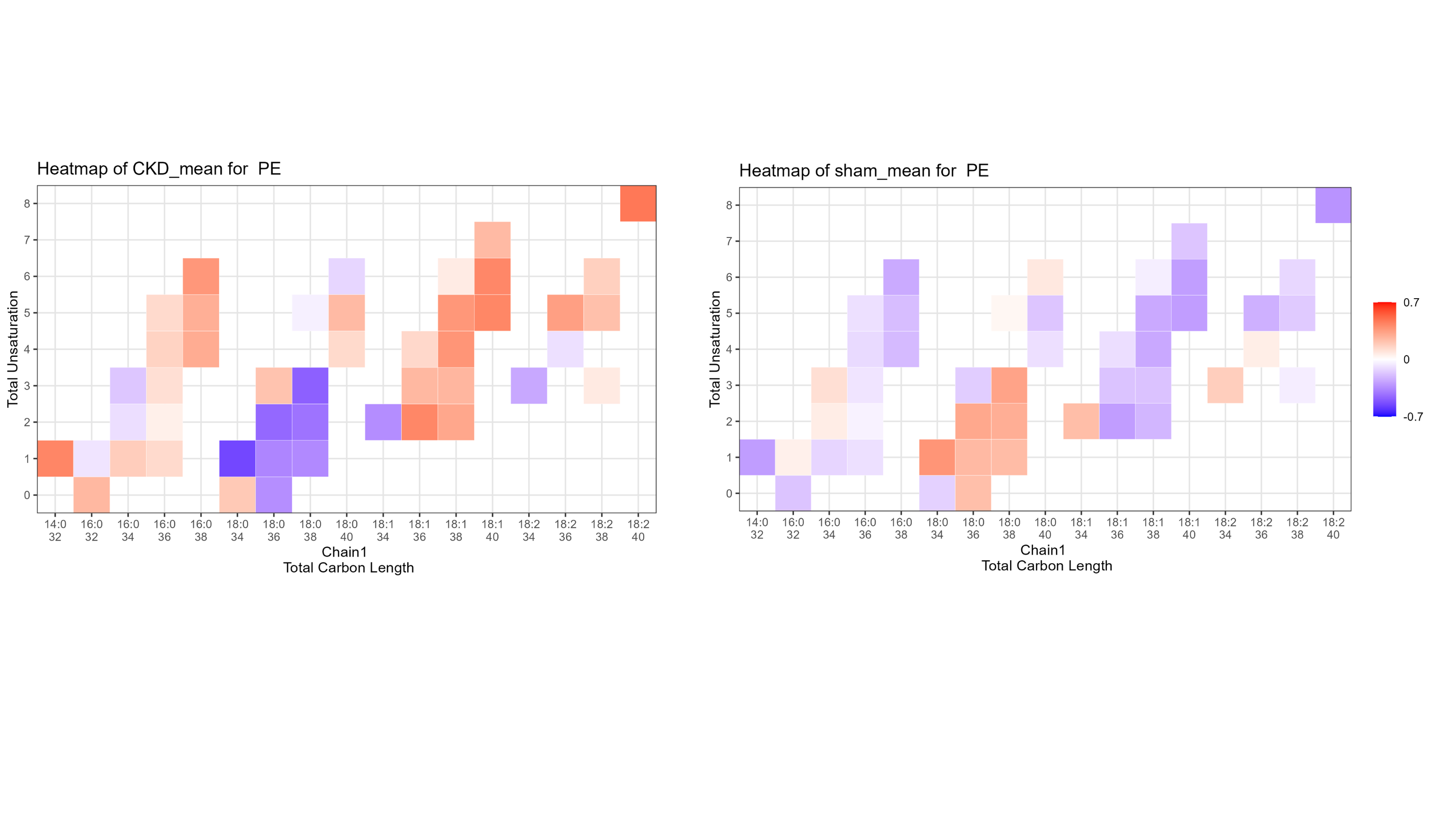
**

# Figure S10: Standardized mean levels of peritoneal macrophage lysophosphatidylethanolamines (LPE) from control (sham) and CKD mice after 16 weeks of high fat diet

**
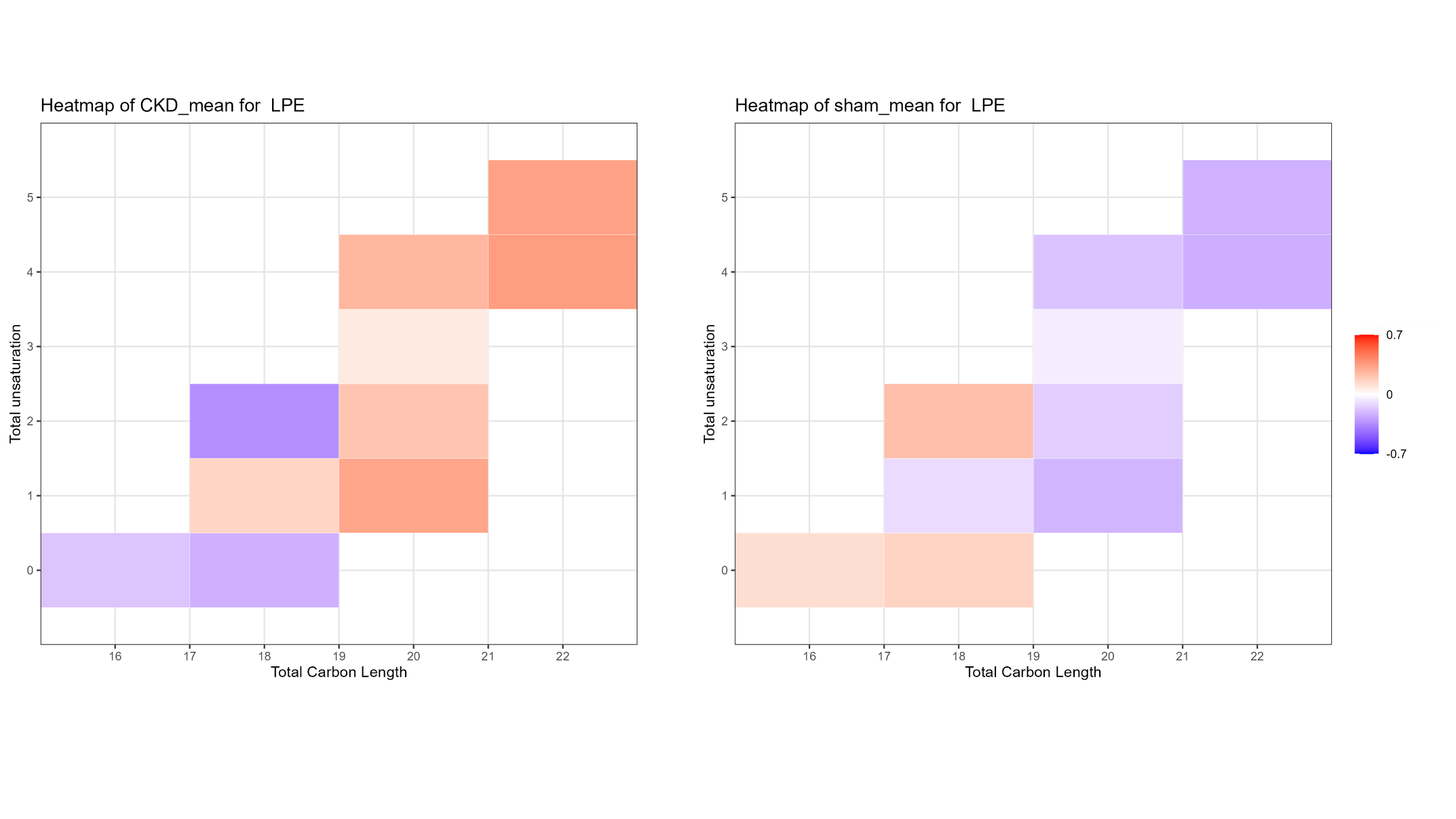
**

**
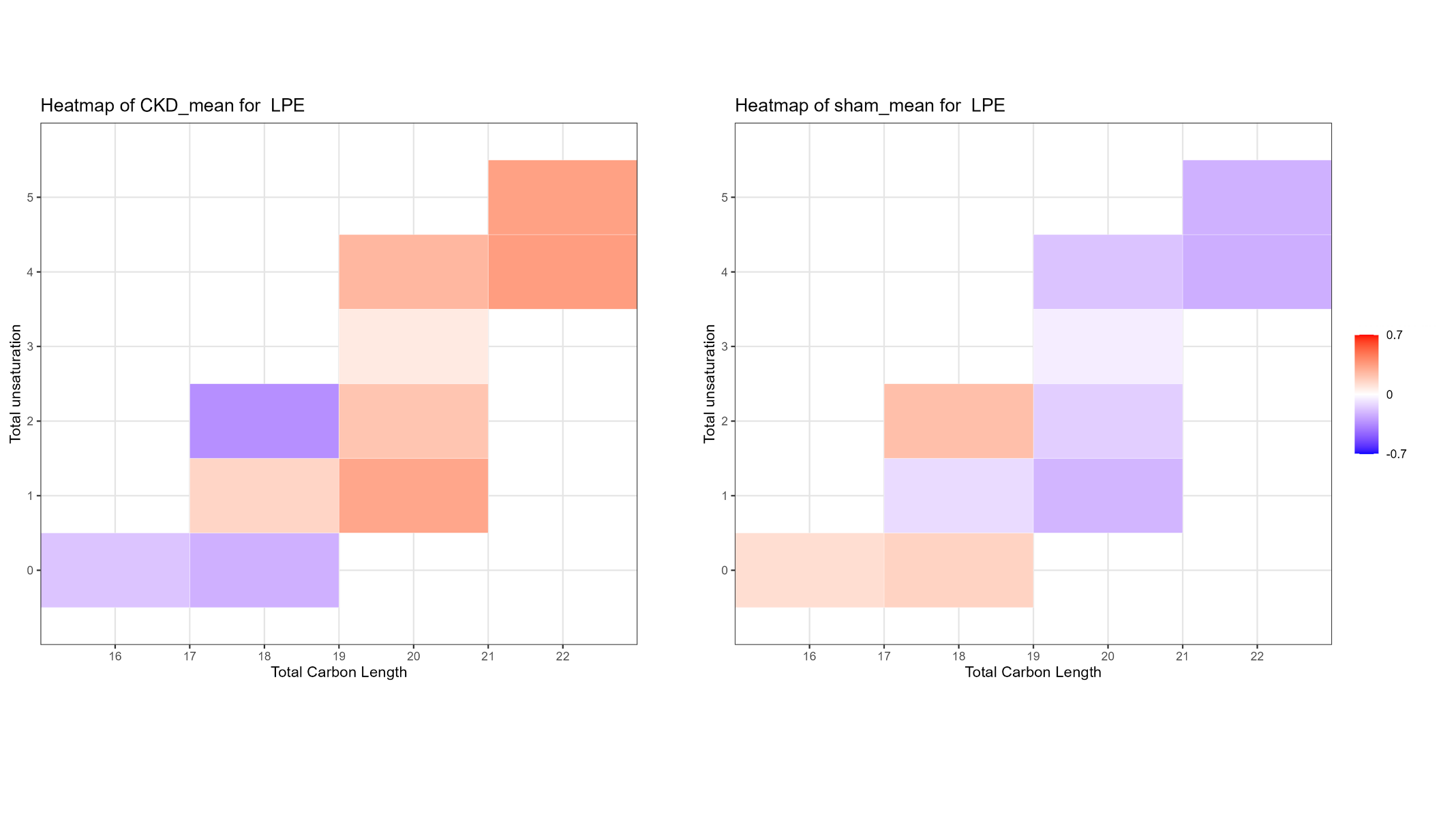
**

# **Figure S11:** Standardized mean levels of peritoneal macrophage PE-O from control (sham) and CKD mice after 16 weeks of high fat diet

**
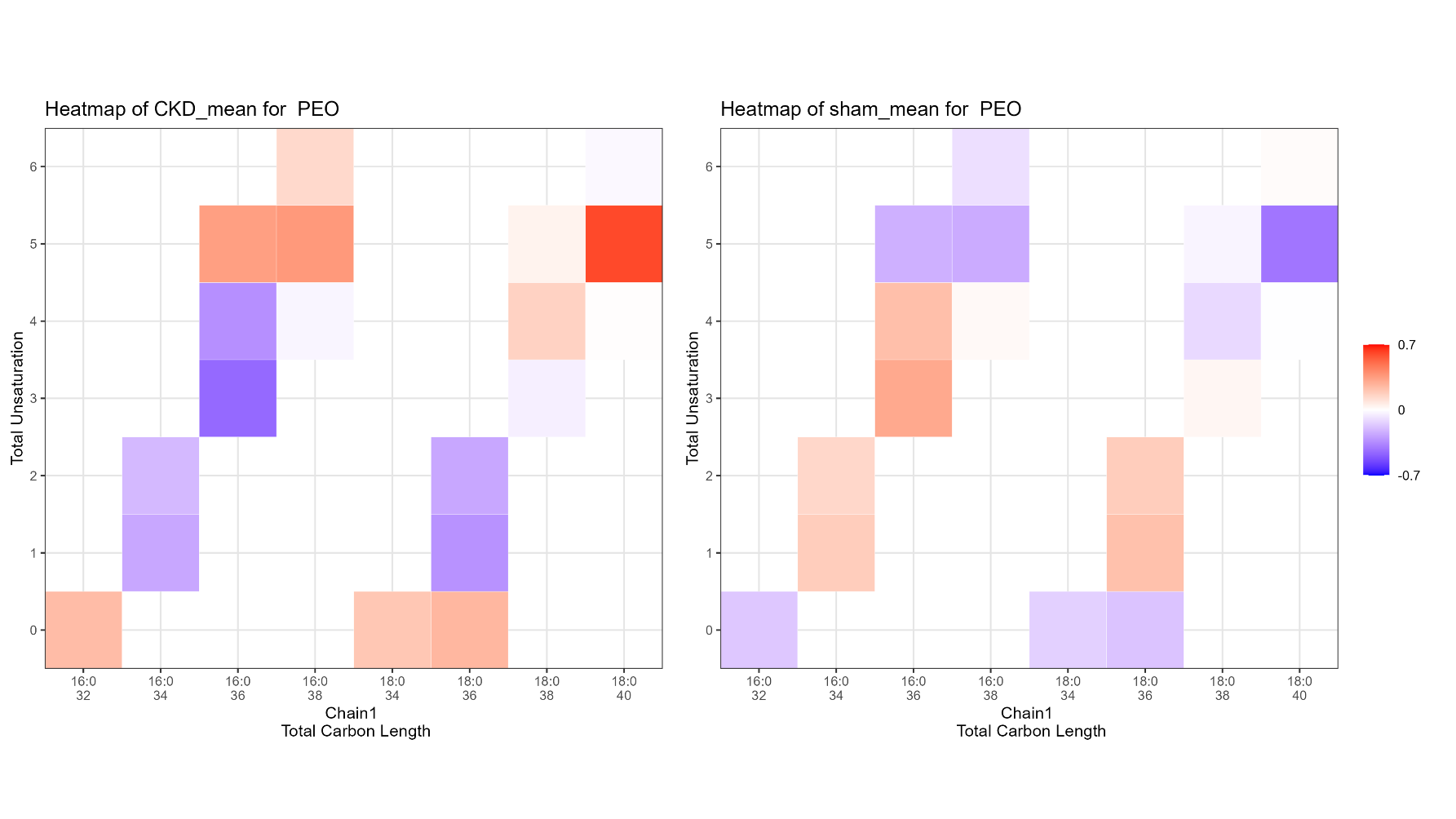
**

**
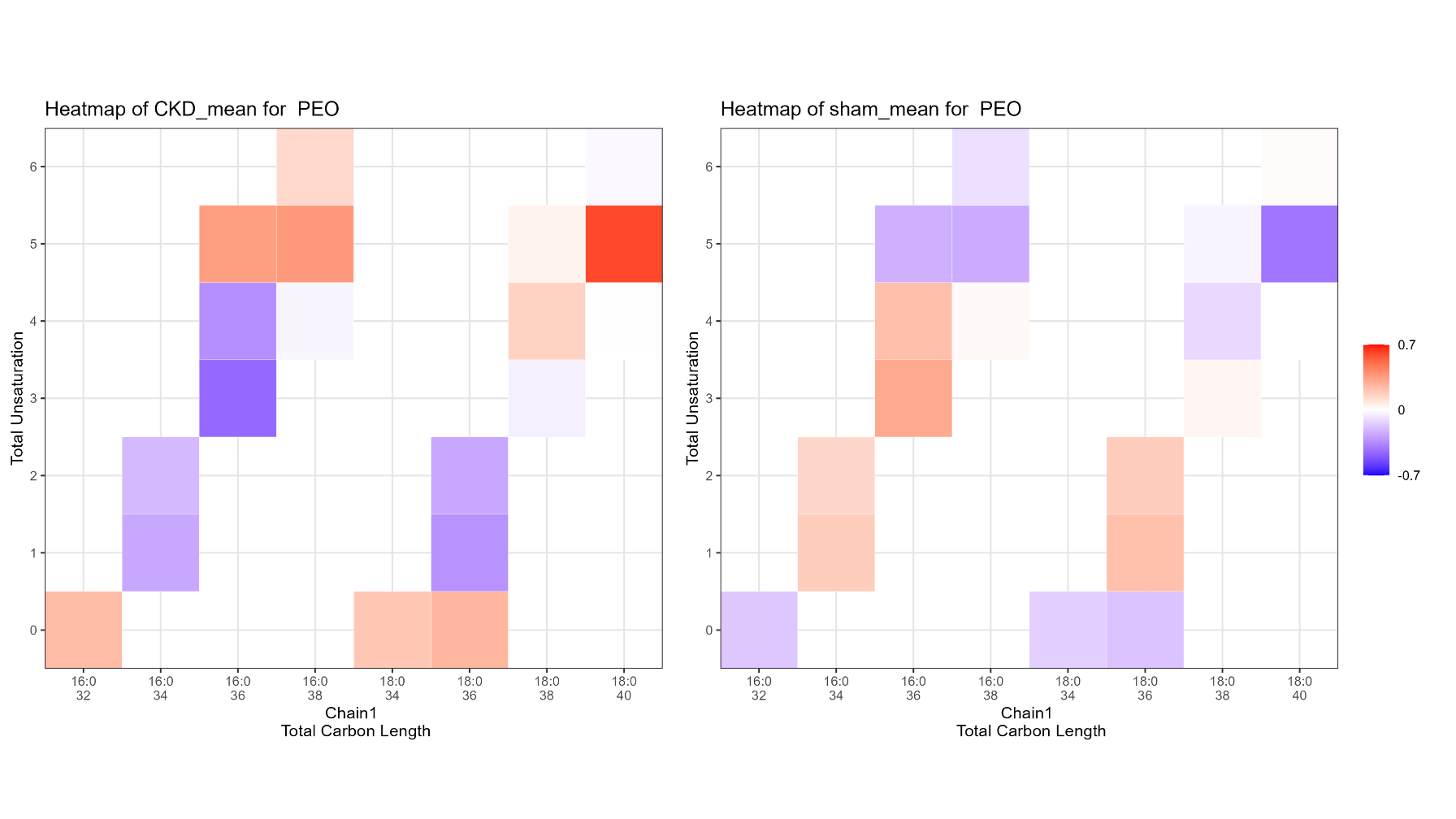
**

# **Figure S12:** Standardized mean levels of peritoneal macrophage PE-P from control (sham) and CKD mice after 16 weeks of high fat diet

**
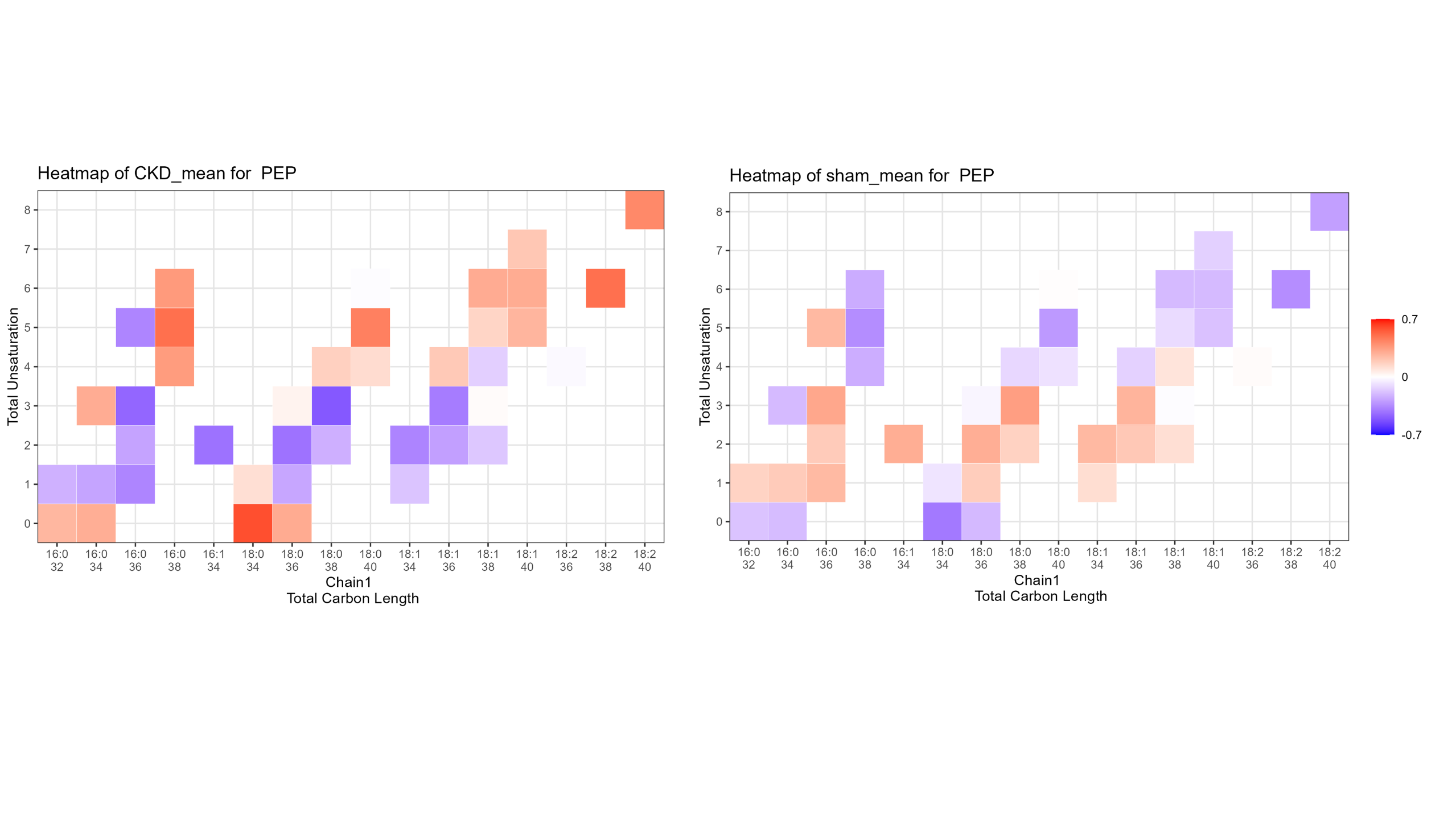
**

**
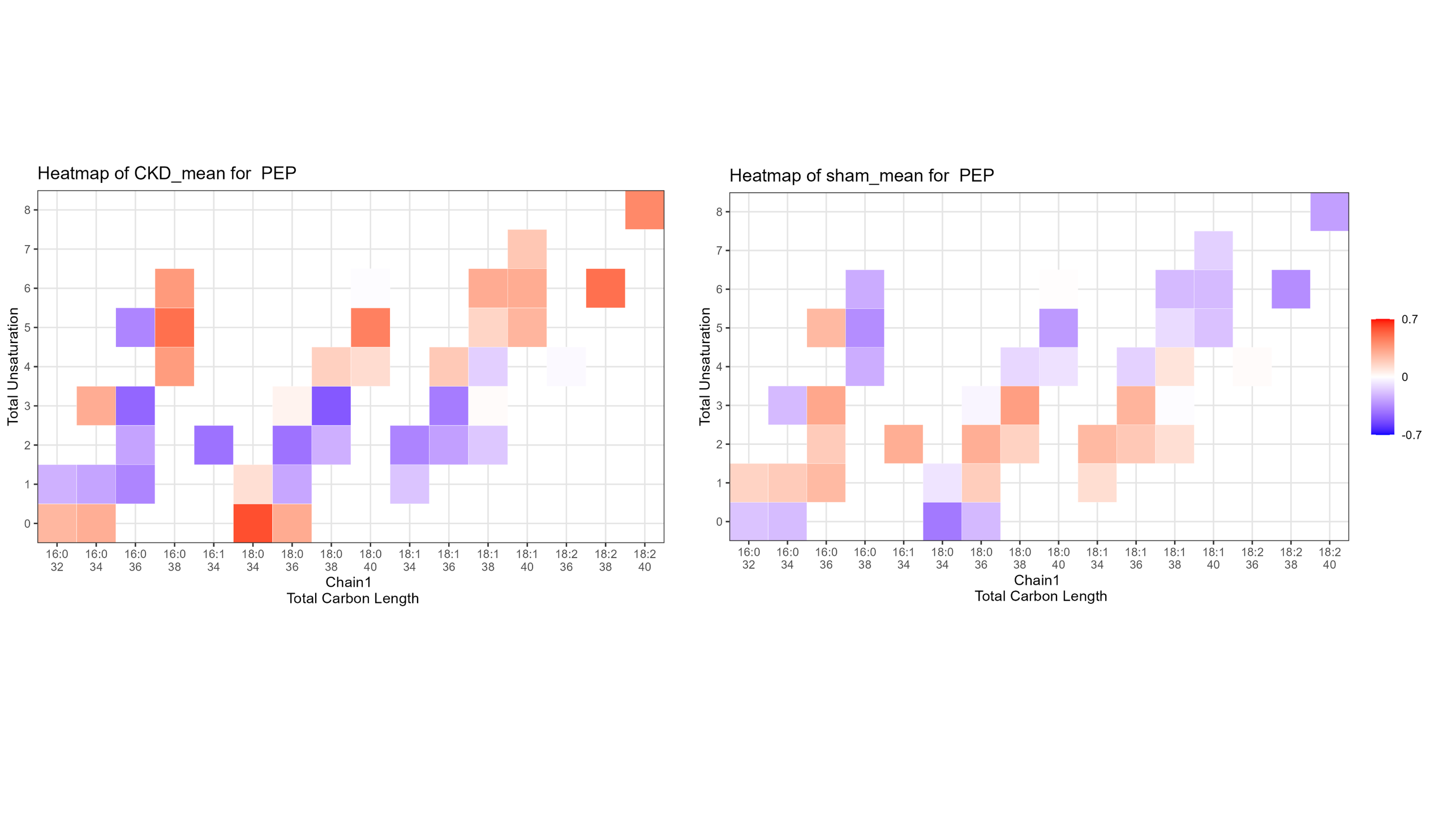
**

# **Figure S13:** Standardized mean levels of peritoneal macrophage phosphatidylglycerols (PG) from control (sham) and CKD mice after 16 weeks of high fat diet

**
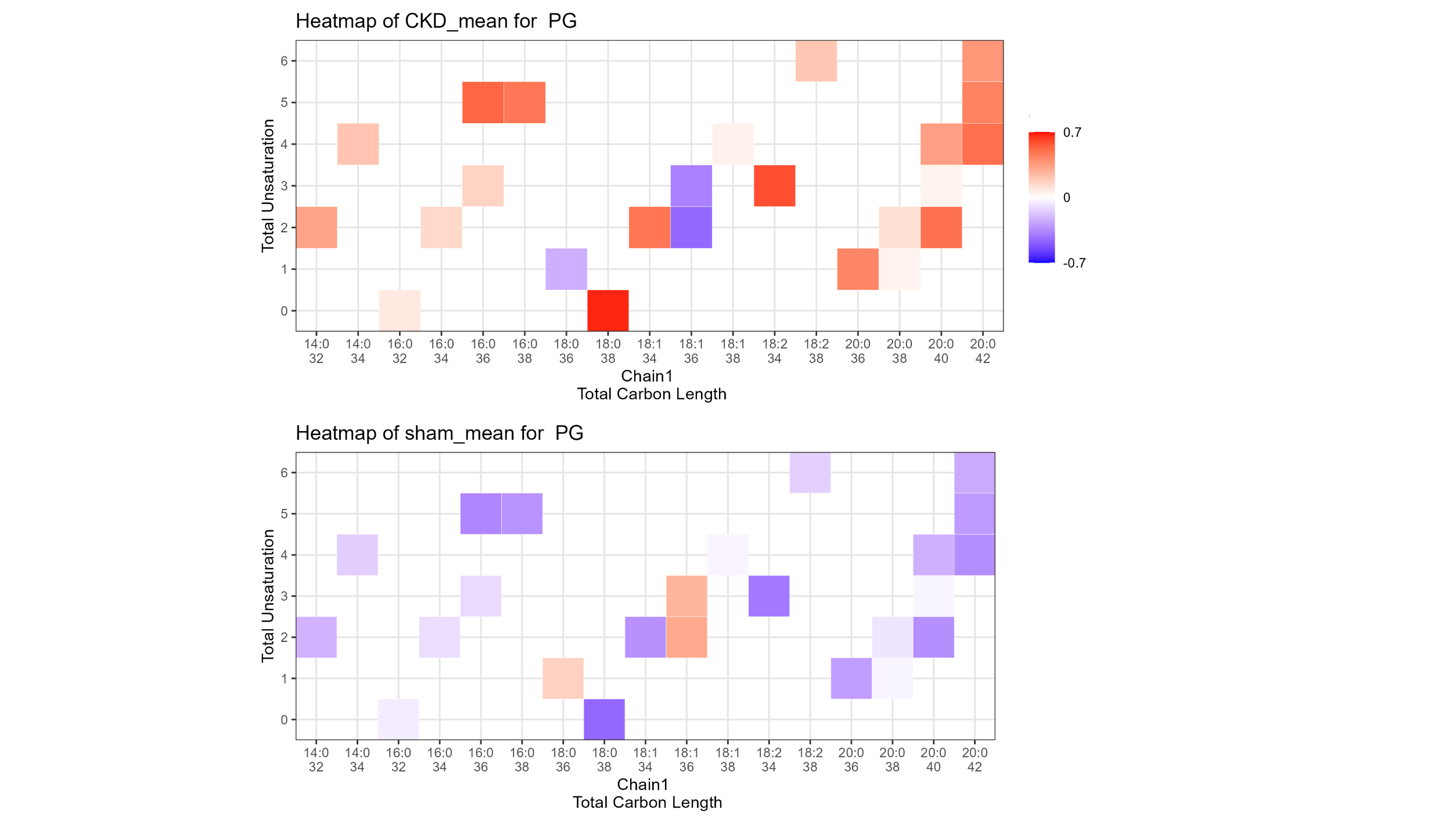
**

# **Figure S14**: Standardized mean levels of peritoneal macrophage phosphatidylinositols (PI) from control (sham) and CKD mice after 16 weeks of high fat diet

**
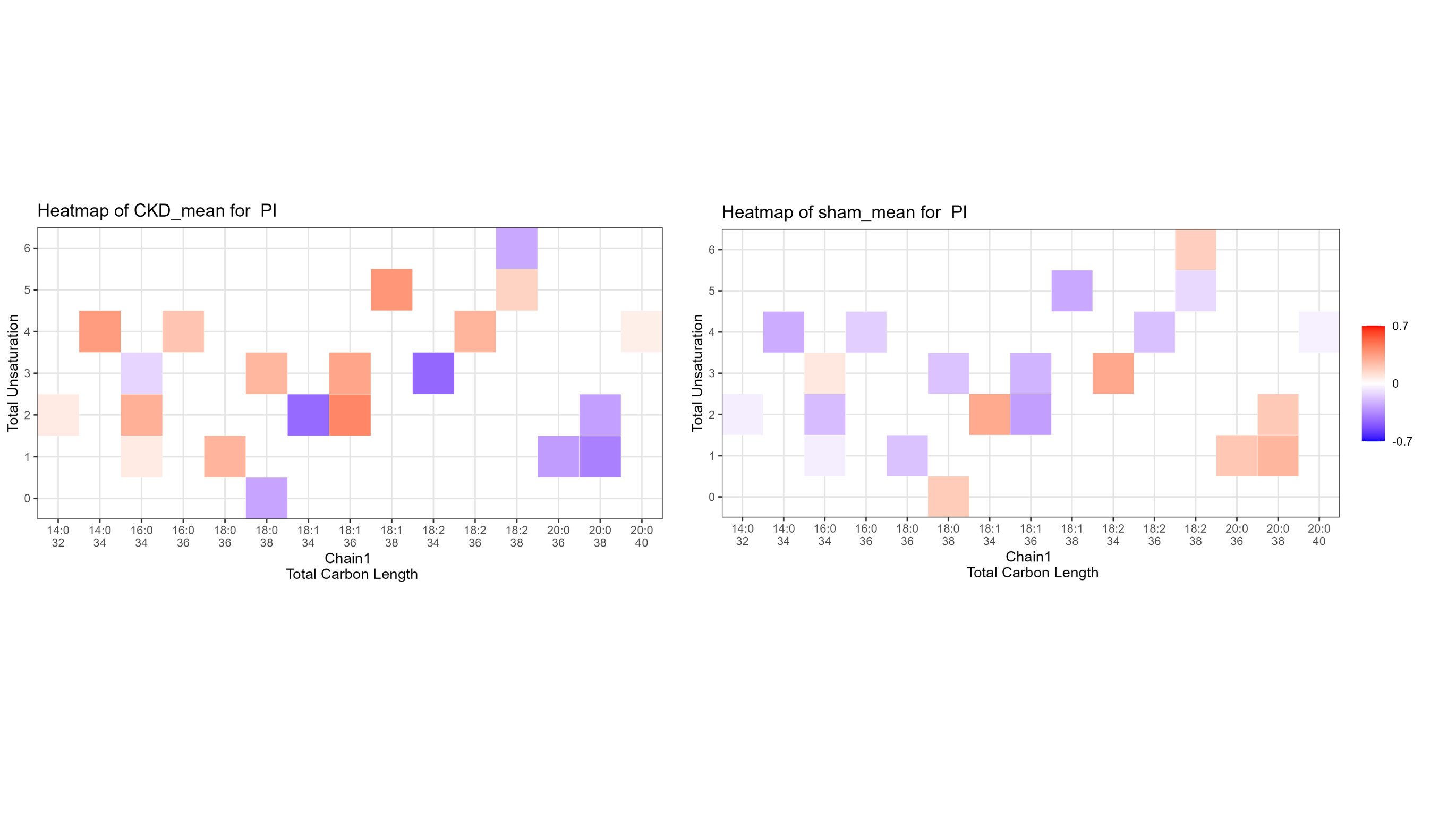
**

**
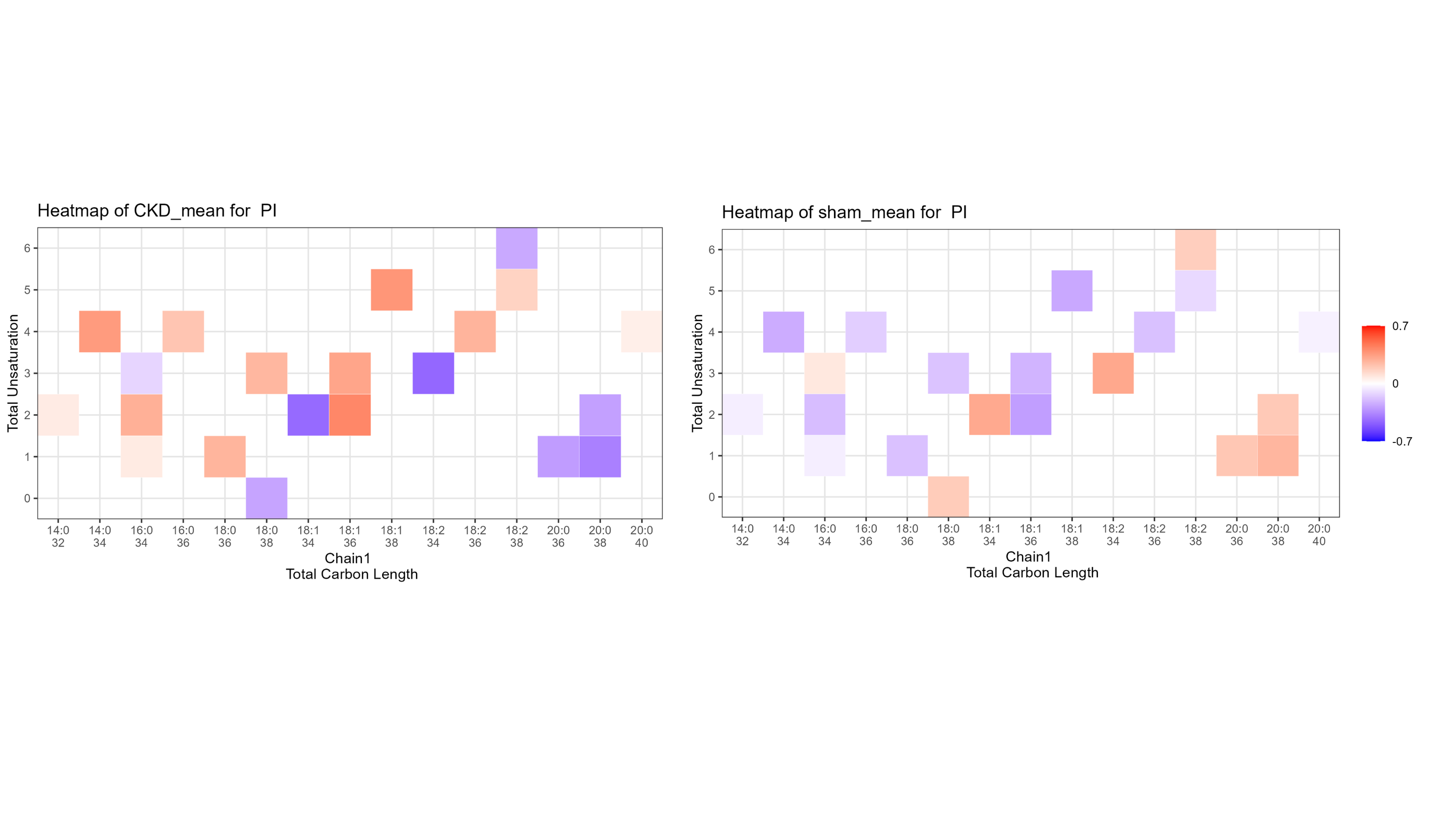
**

# **Figure S15:** Standardized mean levels of peritoneal macrophage phosphatidylserines (PS) from control (sham) and CKD mice after 16 weeks of high fat diet

**
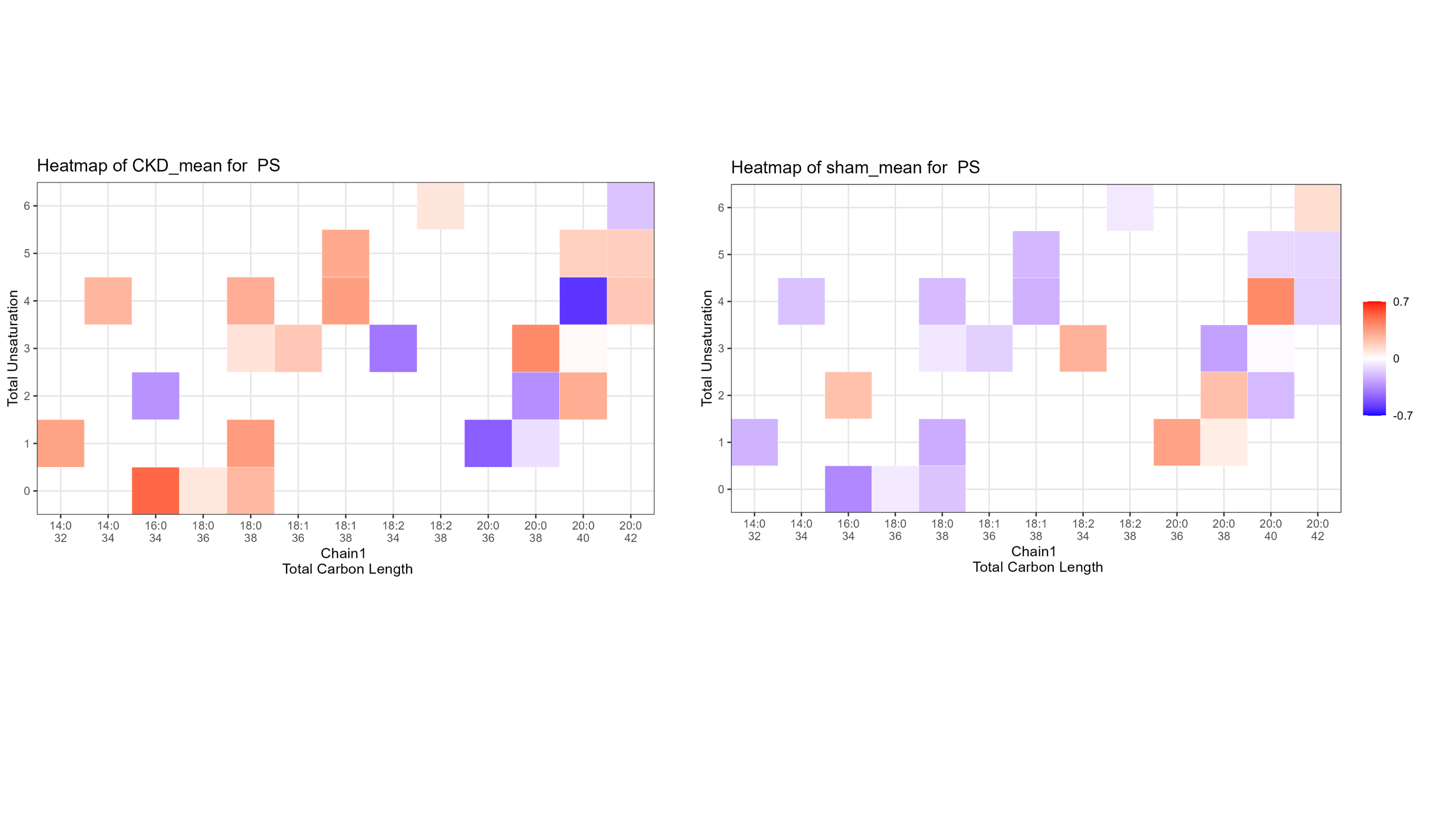
**

**
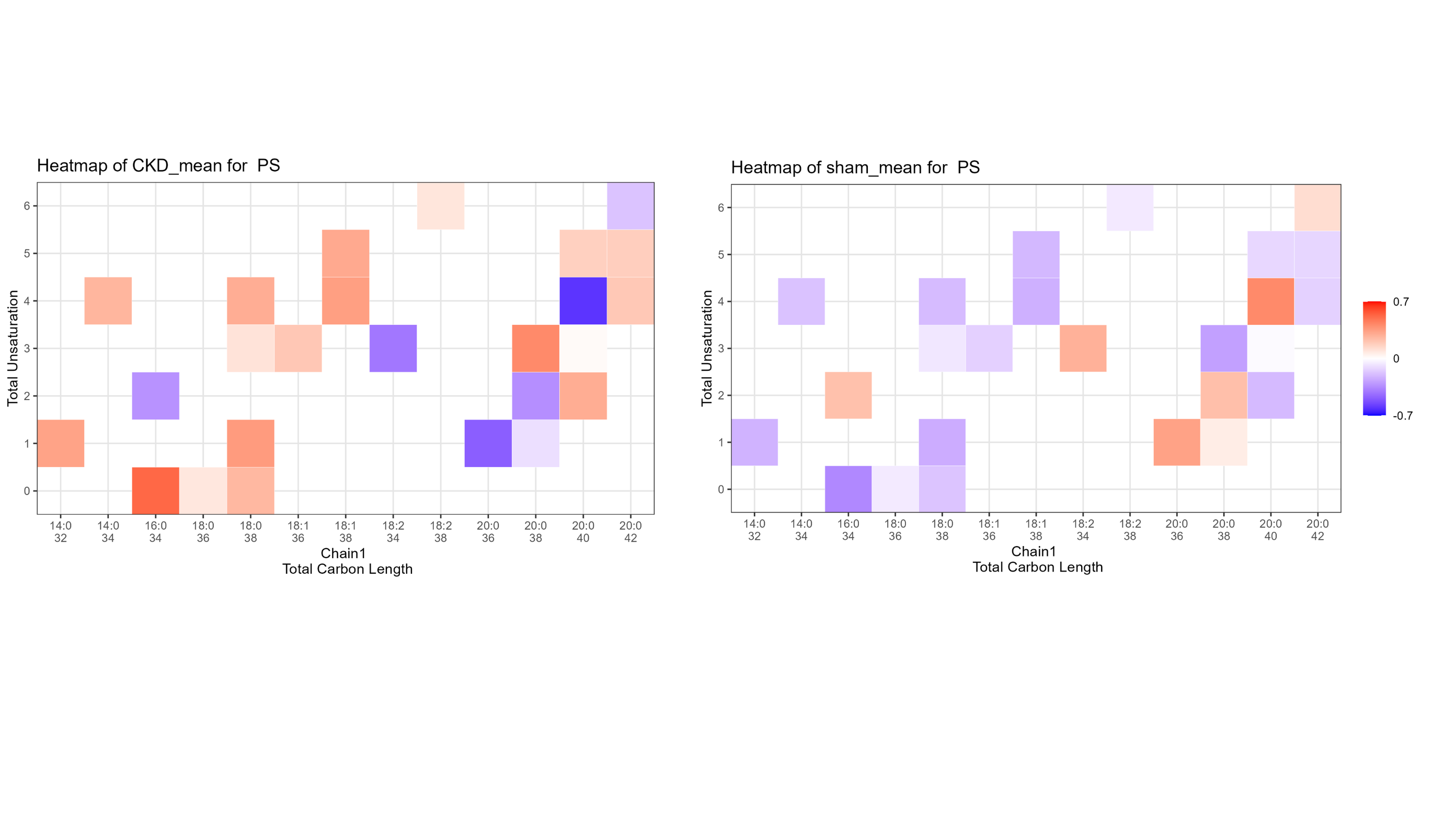
**

# **Figure S16:** Standardized mean levels of peritoneal macrophage lysophosphatidylserines (LPS) from control (sham) and CKD mice after 16 weeks of high fat diet

**
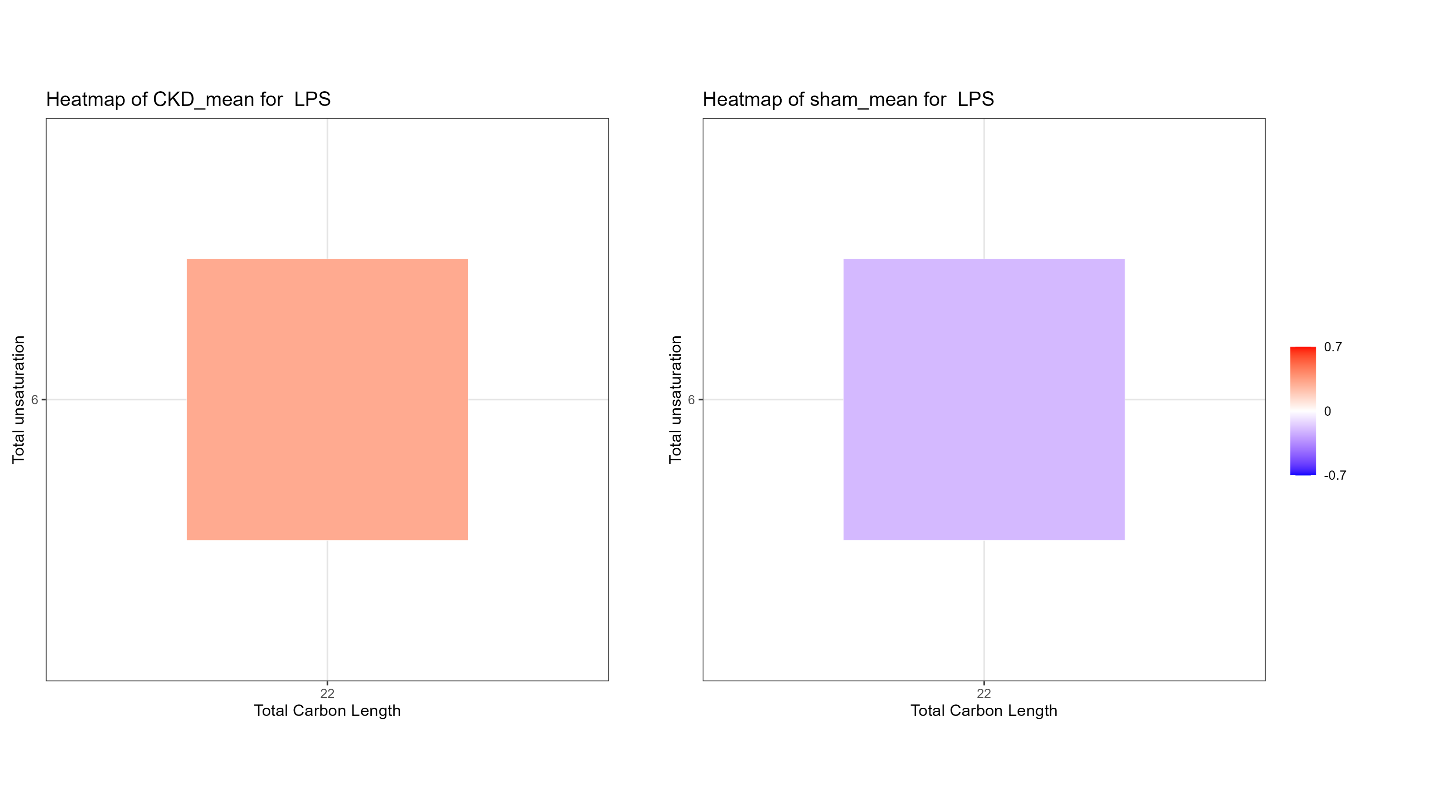
**

# **Figure S17:** Standardized mean levels of peritoneal macrophage ceramides (CER) from control (sham) and CKD mice after 16 weeks of high fat diet

**
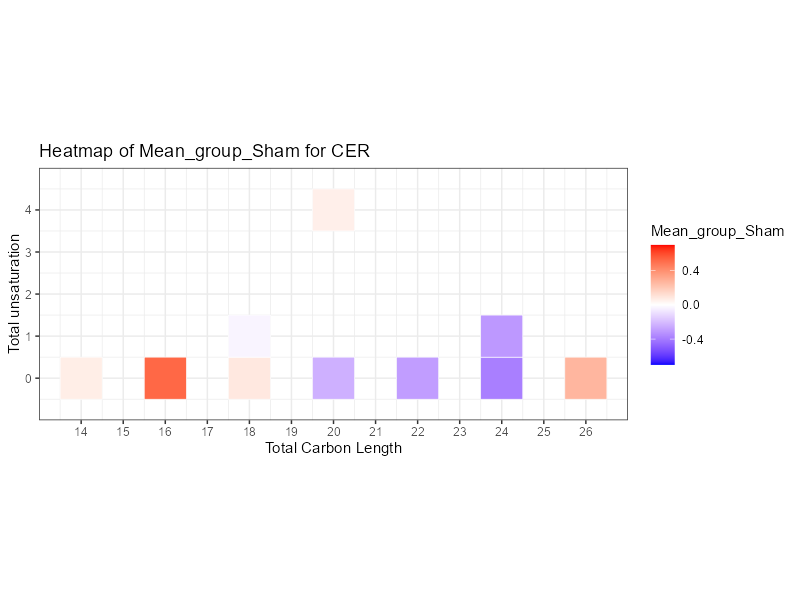

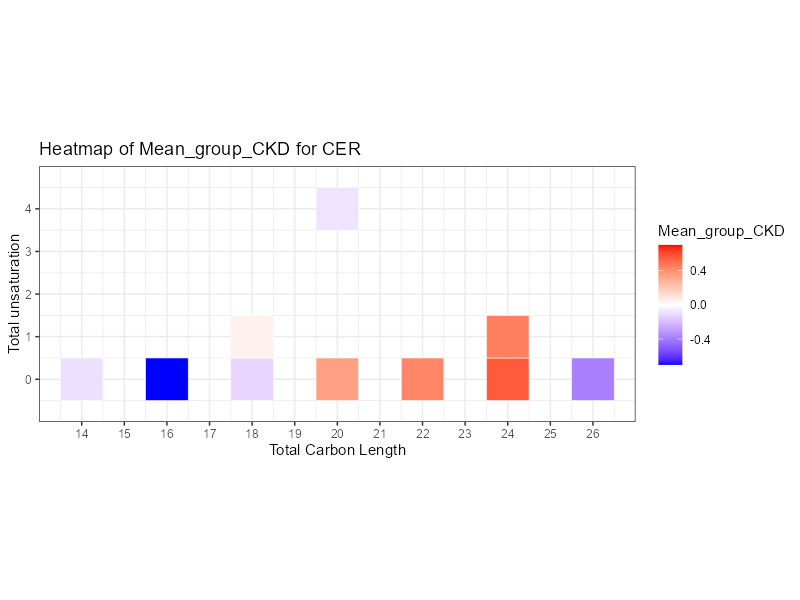
**

# **Figure S18:** Standardized mean levels of peritoneal macrophage dihydroceramides (DCER) from control (sham) and CKD mice after 16 weeks of high fat diet

**
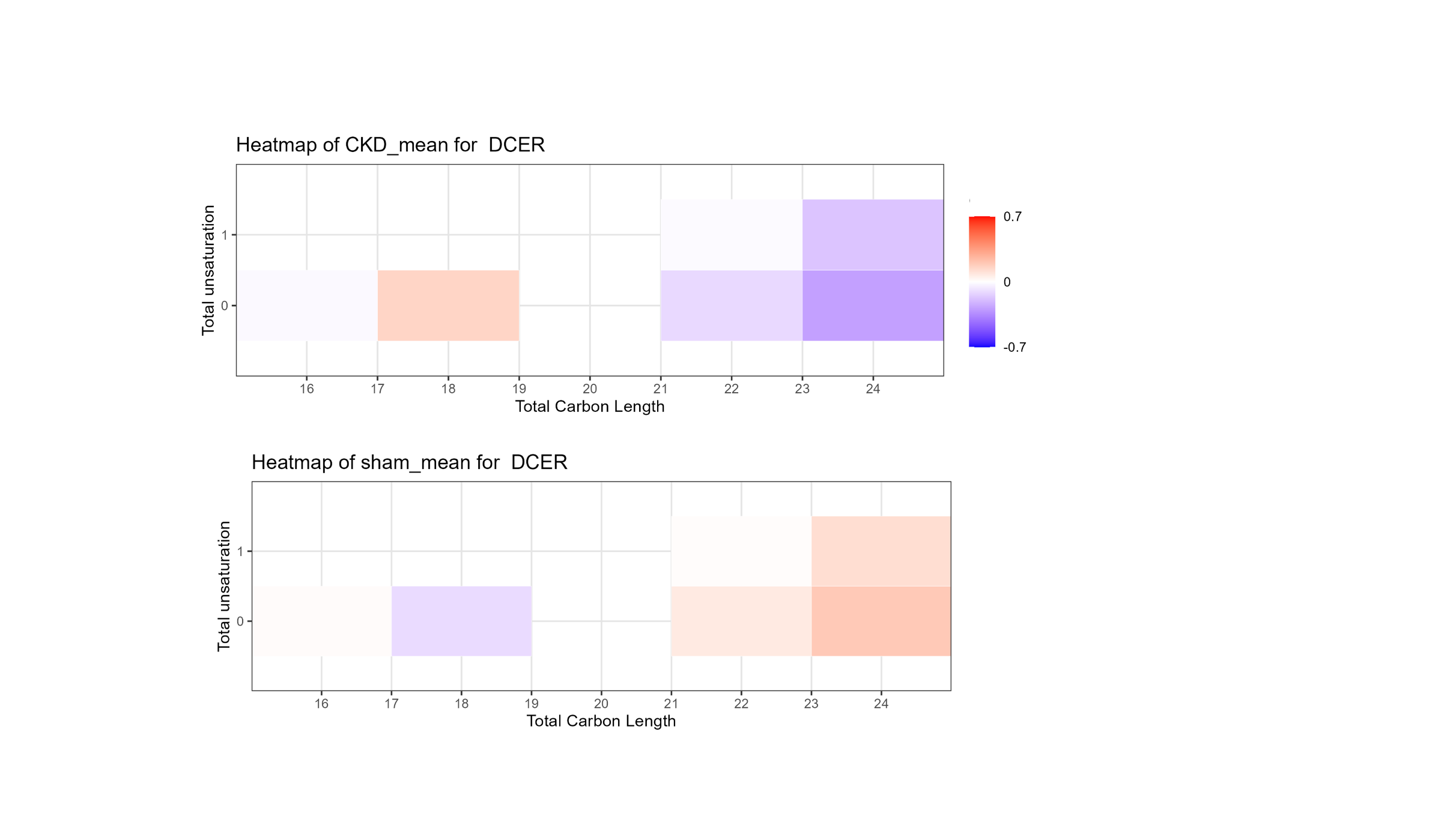
**

# **Figure S19:** Standardized mean levels of peritoneal macrophage hexosylceramides (HCER) from control (sham) and CKD mice after 16 weeks of high fat diet

**
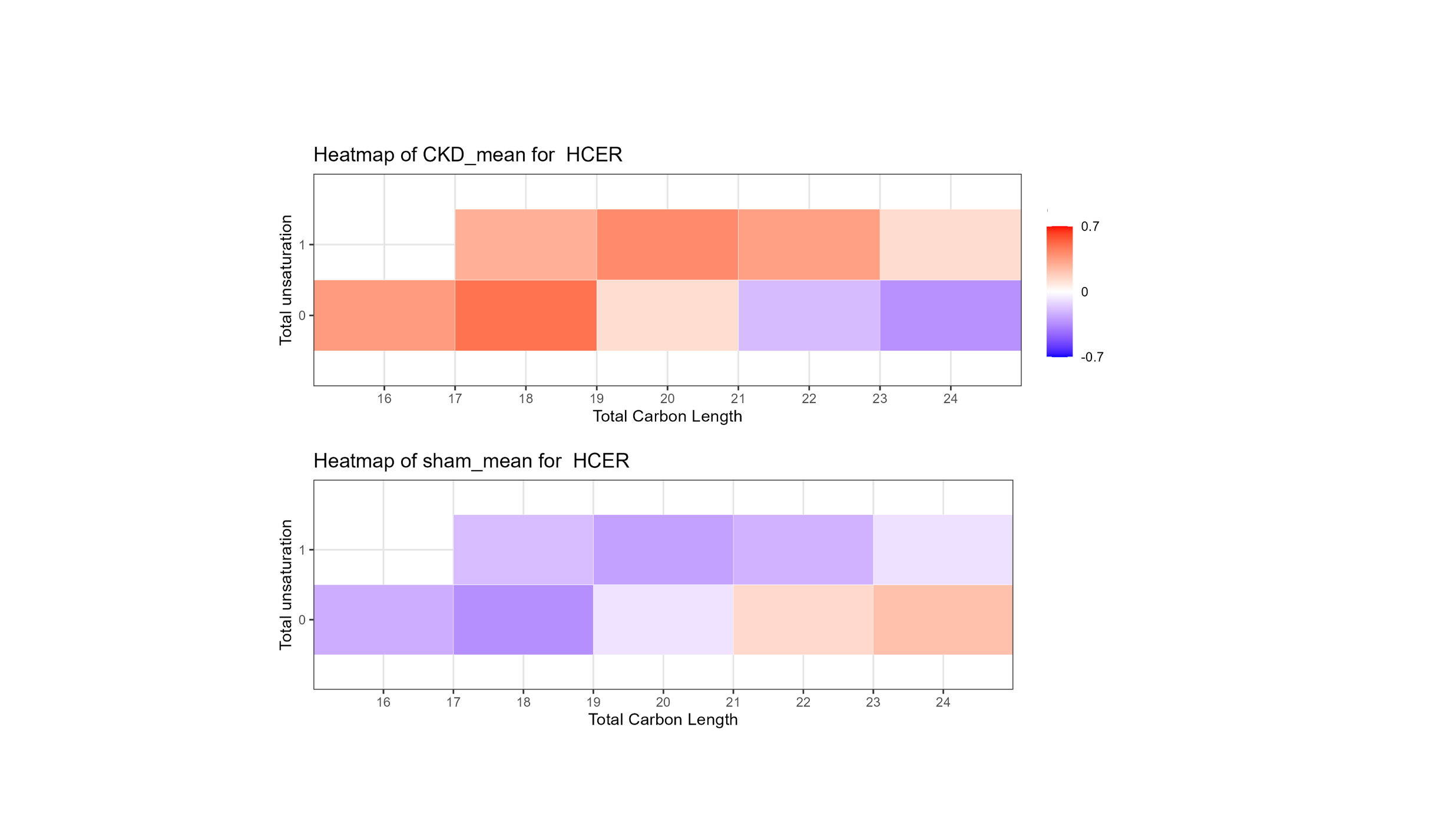
**

# **Figure S20:** Standardized mean levels of peritoneal macrophage lactosylceramides (LCER) from control (sham) and CKD mice after 16 weeks of high fat diet

**
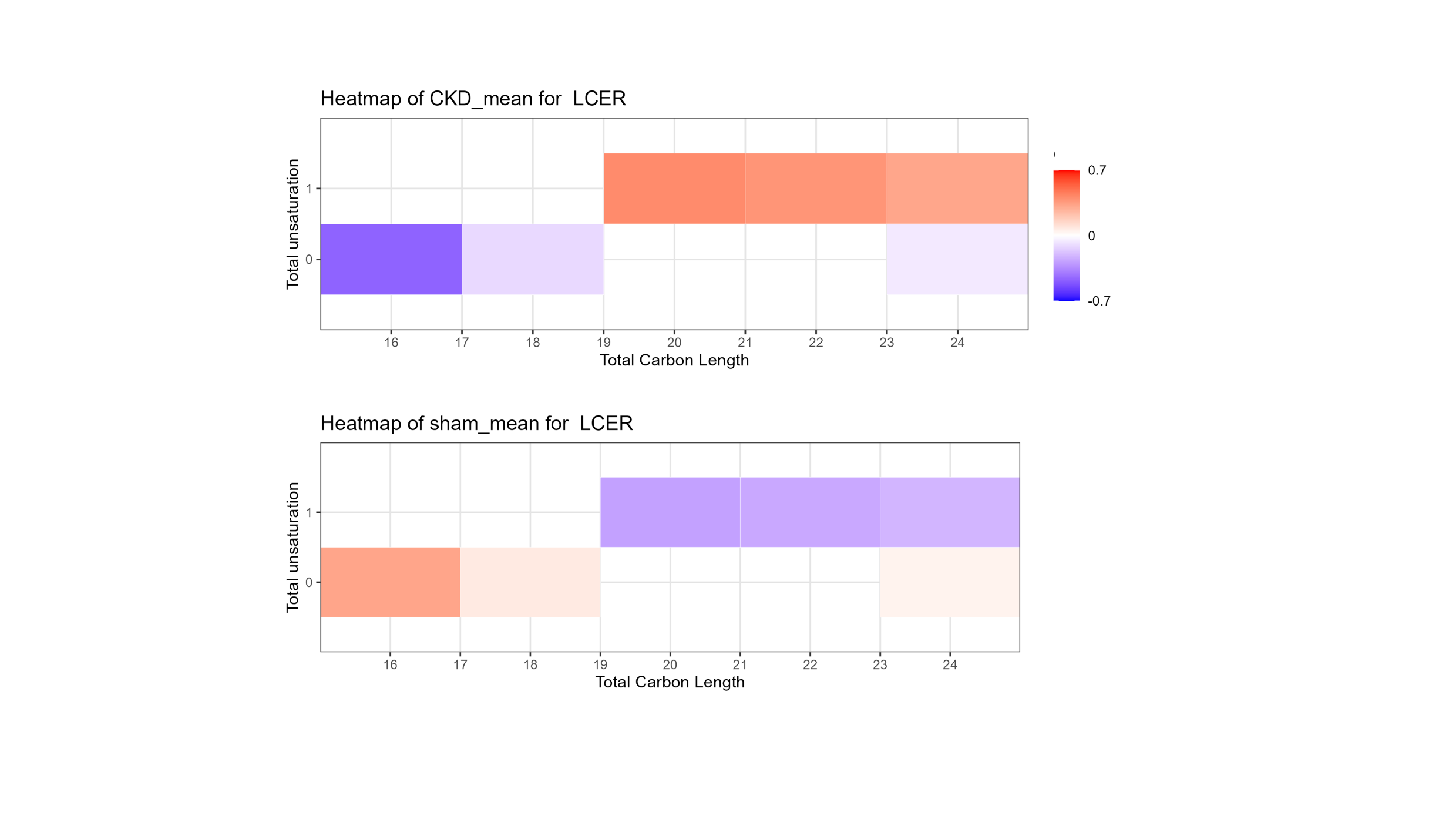
**

# **Figure S21:** Standardized mean levels of peritoneal macrophage sphingomyelins (SM) from control (sham) and CKD mice after 16 weeks of high fat diet

**
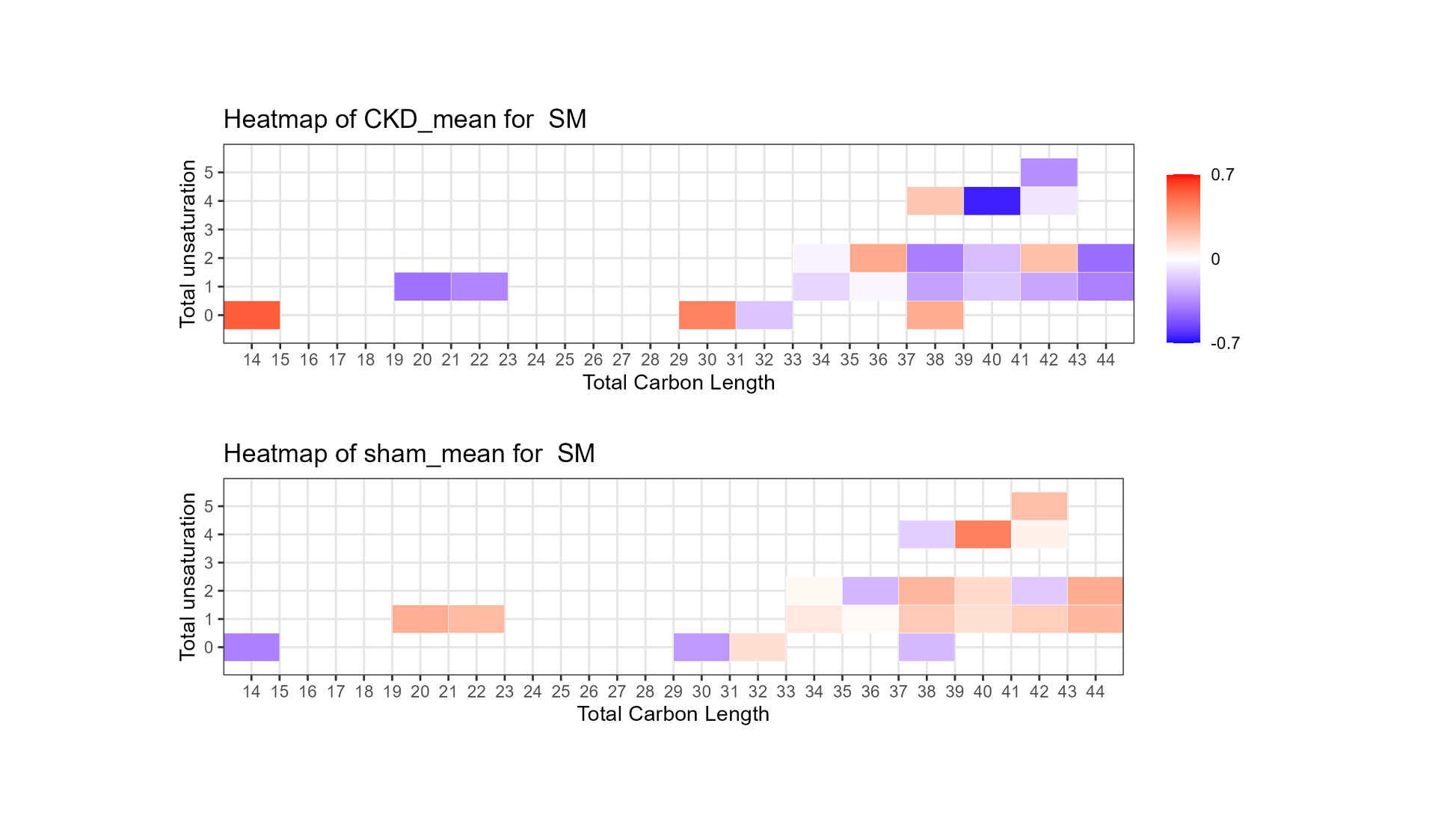
**

**Figure S22: Correlation of saturated FFA levels and glycerolipids by class in sham and CKD peritoneal macrophages.** Pearson correlation coefficients for unsaturated FFA and each lipid by class are shown for CKD (A,C) and sham (B,D) peritoneal macrophages. Significant correlations are shown as positive (red) or negative (blue). P<0.05 using Pearson’s correlation with Fisher’s transformation and FDR correction**
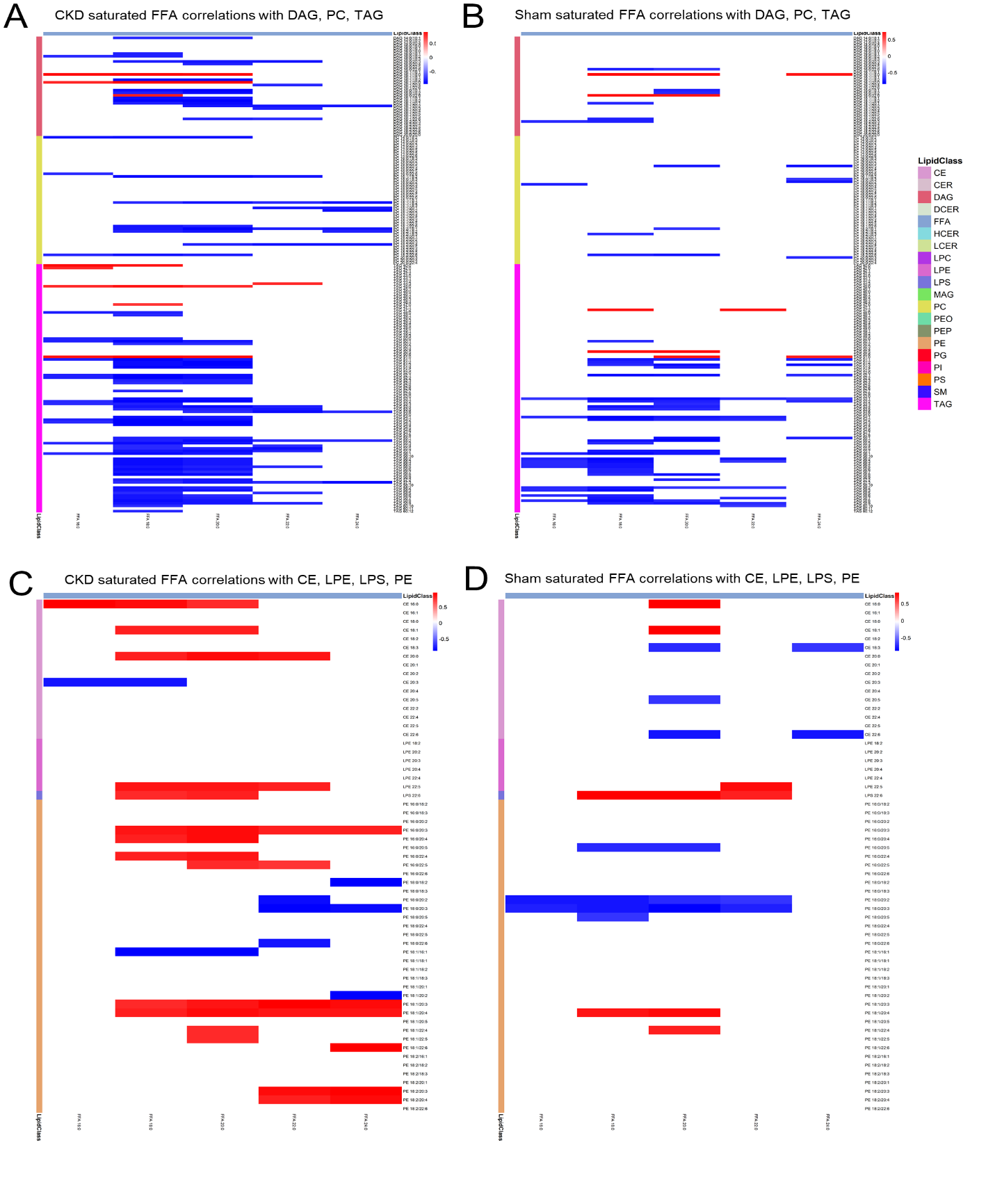
**

**Figure S23: Unsaturated FFA lipid correlation networks in CKD and sham PMΦ** Partial correlation networks for unsaturated In Sham (A) and CKD (B) peritoneal macrophages. Pink edges indicate positive correlations, and blue edges represents negative correlations in each network. The edge thickness reflects the strength of correlation (|0.7|-|1.0|). (C,D). Contigency tables for significant and non-significant partial correlations between groups for saturated (C) and unsaturated (D) FFA correlations. P-value by Fisher Exact test between Sham and CKD groups with respective odds ratio is shown below each table.

**
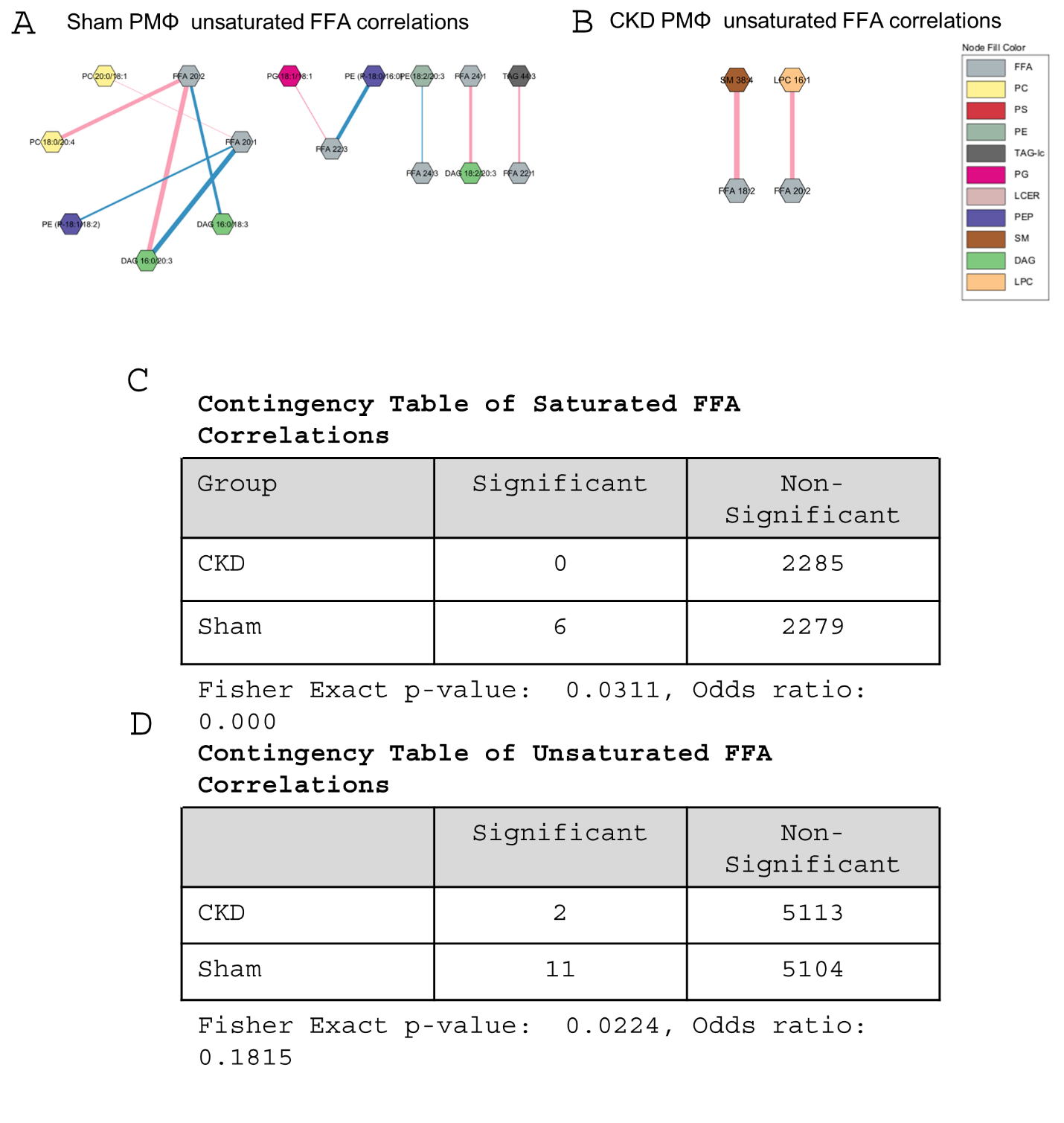
**

**Figure S24: Predicted upregulation of multiple regulators of lipid metabolism in CKD peritoneal macrophages.** Regulatory network by Ingenuity Pathway Analysis (IPA) from CKD peritoneal macrophages predicted upregulation of multiple upstream regulators of lipid metabolism including PPARG, SREB, ACSL1, and HMG CoA synthase.

**
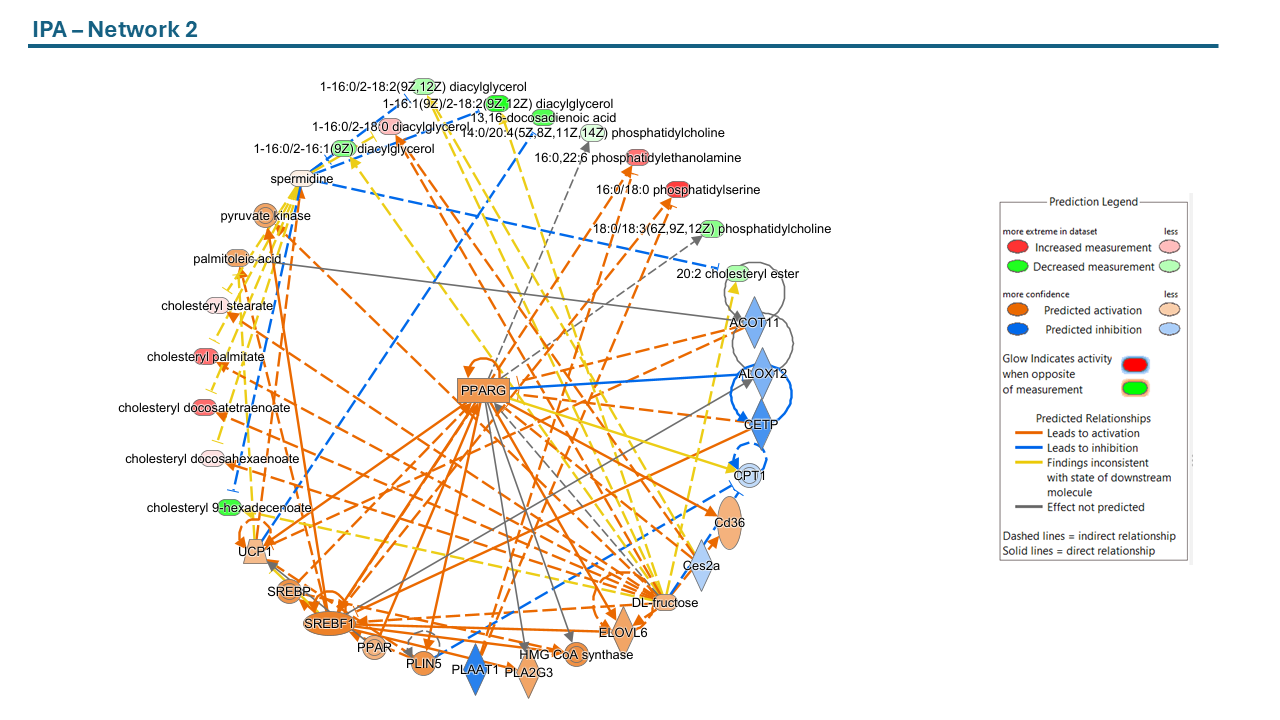

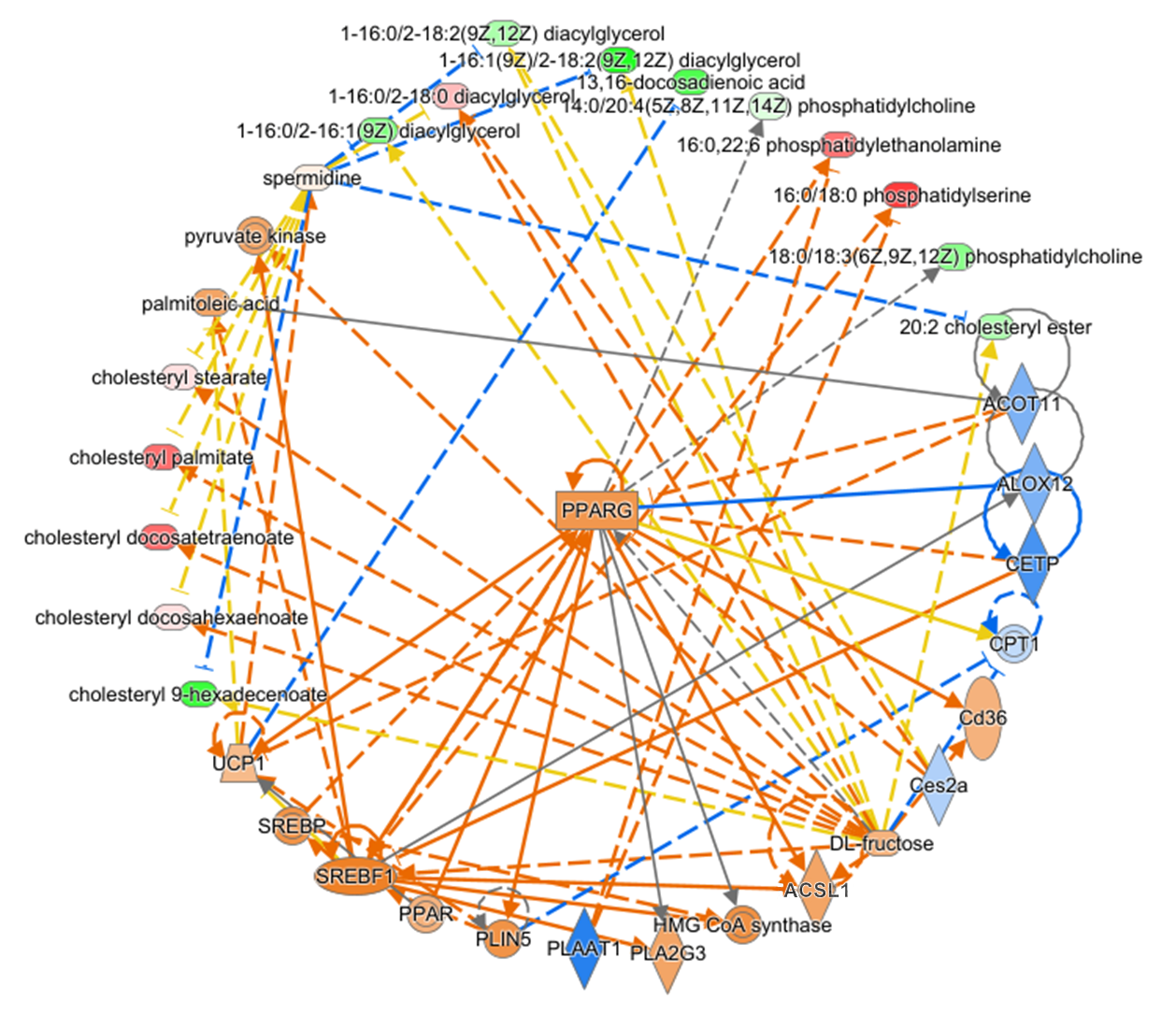
**

# **Table S1:** List of quantified lipids

| **Group** | **Class** | **Name** |
| --- | --- | --- |
| Neutral Lipids | Free fatty acids (FFA) | FFA(16:0) |
|  |  | FFA(18:0) |
|  |  | FFA(18:1) |
|  |  | FFA(18:2) |
|  |  | FFA(20:0) |
|  |  | FFA(20:1) |
|  |  | FFA(20:2) |
|  |  | FFA(20:4) |
|  |  | FFA(22:0) |
|  |  | FFA(22:1) |
|  |  | FFA(22:2) |
|  |  | FFA(22:3) |
|  |  | FFA(24:0) |
|  |  | FFA(24:1) |
|  |  | FFA(24:2) |
|  |  | FFA(24:3) |
|  | Cholesterol Esters (CE) | CE(16:0) |
|  |  | CE(16:1) |
|  |  | CE(18:0) |
|  |  | CE(18:1) |
|  |  | CE(18:2) |
|  |  | CE(18:3) |
|  |  | CE(20:0) |
|  |  | CE(20:1) |
|  |  | CE(20:2) |
|  |  | CE(20:3) |
|  |  | CE(20:4) |
|  |  | CE(20:5) |
|  |  | CE(22:2) |
|  |  | CE(22:4) |
|  |  | CE(22:5) |
|  |  | CE(22:6) |
|  | Monoacylglycerols (MAG) | MAG (22:4) |
|  | Diacylglycerols (DAG) | DAG(14:0/18:1) |
|  |  | DAG(14:0/18:2) |
|  |  | DAG(14:0/20:4) |
|  |  | DAG(16:0/16:0) |
|  |  | DAG(16:0/16:1) |
|  |  | DAG(16:0/18:0) |
|  |  | DAG(16:0/18:1) |
|  |  | DAG(16:0/18:2) |
|  |  | DAG(16:0/18:3) |
|  |  | DAG(16:0/20:3) |
|  |  | DAG(16:0/20:4) |
|  |  | DAG(16:0/22:5) |
|  |  | DAG(16:0/22:6) |
|  |  | DAG(16:1/16:1) |
|  |  | DAG(16:1/18:0) |
|  |  | DAG(16:1/18:1) |
|  |  | DAG(16:1/18:2) |
|  |  | DAG(16:1/20:0) |
|  |  | DAG(16:1/20:4) |
|  |  | DAG(16:1/22:6) |
|  |  | DAG(18:0/18:1) |
|  |  | DAG(18:0/18:2) |
|  |  | DAG(18:0/22:6) |
|  |  | DAG(18:1/18:1) |
|  |  | DAG(18:1/18:2) |
|  |  | DAG(18:1/20:1) |
|  |  | DAG(18:1/20:2) |
|  |  | DAG(18:1/20:3) |
|  |  | DAG(18:1/20:4) |
|  |  | DAG(18:1/20:5) |
|  |  | DAG(18:1/22:5) |
|  |  | DAG(18:1/22:6) |
|  |  | DAG(18:2/20:3) |
|  |  | DAG(18:2/20:4) |
|  |  | DAG(18:2/22:4) |
|  |  | DAG(18:2/22:5) |
|  |  | DAG(18:2/22:6) |
|  |  | DAG(20:0/20:0) |
|  | Triacylglycerols (TAG) | TAG40:0-FA14:0 |
|  |  | TAG40:0-FA16:0 |
|  |  | TAG42:0-FA14:0 |
|  |  | TAG42:0-FA16:0 |
|  |  | TAG42:1-FA14:0 |
|  |  | TAG42:1-FA16:0 |
|  |  | TAG42:1-FA16:1 |
|  |  | TAG42:1-FA18:1 |
|  |  | TAG42:2-FA18:2 |
|  |  | TAG44:0-FA14:0 |
|  |  | TAG44:0-FA16:0 |
|  |  | TAG44:0-FA18:0 |
|  |  | TAG44:1-FA14:0 |
|  |  | TAG44:1-FA16:0 |
|  |  | TAG44:1-FA16:1 |
|  |  | TAG44:1-FA18:1 |
|  |  | TAG44:2-FA14:0 |
|  |  | TAG44:2-FA16:0 |
|  |  | TAG44:2-FA16:1 |
|  |  | TAG44:2-FA18:1 |
|  |  | TAG44:2-FA18:2 |
|  |  | TAG44:3-FA18:2 |
|  |  | TAG45:0-FA14:0 |
|  |  | TAG45:0-FA16:0 |
|  |  | TAG45:1-FA16:0 |
|  |  | TAG45:1-FA18:1 |
|  |  | TAG46:0-FA14:0 |
|  |  | TAG46:0-FA16:0 |
|  |  | TAG46:0-FA18:0 |
|  |  | TAG46:1-FA14:0 |
|  |  | TAG46:1-FA16:0 |
|  |  | TAG46:1-FA16:1 |
|  |  | TAG46:1-FA18:0 |
|  |  | TAG46:1-FA18:1 |
|  |  | TAG46:2-FA14:0 |
|  |  | TAG46:2-FA16:0 |
|  |  | TAG46:2-FA16:1 |
|  |  | TAG46:2-FA18:1 |
|  |  | TAG46:2-FA18:2 |
|  |  | TAG46:3-FA14:0 |
|  |  | TAG46:3-FA16:0 |
|  |  | TAG46:3-FA16:1 |
|  |  | TAG46:3-FA18:1 |
|  |  | TAG46:3-FA18:2 |
|  |  | TAG46:3-FA18:3 |
|  |  | TAG46:4-FA18:2 |
|  |  | TAG47:0-FA14:0 |
|  |  | TAG47:0-FA16:0 |
|  |  | TAG47:0-FA17:0 |
|  |  | TAG47:1-FA14:0 |
|  |  | TAG47:1-FA16:0 |
|  |  | TAG47:1-FA16:1 |
|  |  | TAG47:1-FA17:0 |
|  |  | TAG47:1-FA18:1 |
|  |  | TAG47:2-FA14:0 |
|  |  | TAG47:2-FA16:1 |
|  |  | TAG47:2-FA18:1 |
|  |  | TAG47:2-FA18:2 |
|  |  | TAG48:0-FA14:0 |
|  |  | TAG48:0-FA16:0 |
|  |  | TAG48:0-FA18:0 |
|  |  | TAG48:1-FA14:0 |
|  |  | TAG48:1-FA16:0 |
|  |  | TAG48:1-FA16:1 |
|  |  | TAG48:1-FA18:0 |
|  |  | TAG48:1-FA18:1 |
|  |  | TAG48:2-FA14:0 |
|  |  | TAG48:2-FA16:0 |
|  |  | TAG48:2-FA16:1 |
|  |  | TAG48:2-FA18:0 |
|  |  | TAG48:2-FA18:1 |
|  |  | TAG48:2-FA18:2 |
|  |  | TAG48:3-FA14:0 |
|  |  | TAG48:3-FA16:0 |
|  |  | TAG48:3-FA16:1 |
|  |  | TAG48:3-FA18:1 |
|  |  | TAG48:3-FA18:2 |
|  |  | TAG48:3-FA18:3 |
|  |  | TAG48:4-FA14:0 |
|  |  | TAG48:4-FA16:0 |
|  |  | TAG48:4-FA16:1 |
|  |  | TAG48:4-FA18:1 |
|  |  | TAG48:4-FA18:2 |
|  |  | TAG48:4-FA18:3 |
|  |  | TAG48:4-FA20:4 |
|  |  | TAG48:5-FA18:2 |
|  |  | TAG48:5-FA18:3 |
|  |  | TAG49:0-FA16:0 |
|  |  | TAG49:0-FA17:0 |
|  |  | TAG49:0-FA18:0 |
|  |  | TAG49:1-FA14:0 |
|  |  | TAG49:1-FA16:0 |
|  |  | TAG49:1-FA16:1 |
|  |  | TAG49:1-FA17:0 |
|  |  | TAG49:1-FA18:1 |
|  |  | TAG49:2-FA14:0 |
|  |  | TAG49:2-FA16:0 |
|  |  | TAG49:2-FA16:1 |
|  |  | TAG49:2-FA17:0 |
|  |  | TAG49:2-FA18:1 |
|  |  | TAG49:2-FA18:2 |
|  |  | TAG49:3-FA16:0 |
|  |  | TAG49:3-FA16:1 |
|  |  | TAG49:3-FA18:2 |
|  |  | TAG49:3-FA18:3 |
|  |  | TAG50:0-FA14:0 |
|  |  | TAG50:0-FA16:0 |
|  |  | TAG50:0-FA18:0 |
|  |  | TAG50:1-FA14:0 |
|  |  | TAG50:1-FA16:0 |
|  |  | TAG50:1-FA16:1 |
|  |  | TAG50:1-FA18:0 |
|  |  | TAG50:1-FA18:1 |
|  |  | TAG50:1-FA20:1 |
|  |  | TAG50:2-FA14:0 |
|  |  | TAG50:2-FA16:0 |
|  |  | TAG50:2-FA16:1 |
|  |  | TAG50:2-FA18:0 |
|  |  | TAG50:2-FA18:1 |
|  |  | TAG50:2-FA18:2 |
|  |  | TAG50:2-FA20:2 |
|  |  | TAG50:3-FA14:0 |
|  |  | TAG50:3-FA16:0 |
|  |  | TAG50:3-FA16:1 |
|  |  | TAG50:3-FA18:0 |
|  |  | TAG50:3-FA18:1 |
|  |  | TAG50:3-FA18:2 |
|  |  | TAG50:3-FA18:3 |
|  |  | TAG50:3-FA20:3 |
|  |  | TAG50:4-FA14:0 |
|  |  | TAG50:4-FA16:0 |
|  |  | TAG50:4-FA16:1 |
|  |  | TAG50:4-FA18:1 |
|  |  | TAG50:4-FA18:2 |
|  |  | TAG50:4-FA18:3 |
|  |  | TAG50:4-FA20:3 |
|  |  | TAG50:4-FA20:4 |
|  |  | TAG50:5-FA14:0 |
|  |  | TAG50:5-FA16:0 |
|  |  | TAG50:5-FA16:1 |
|  |  | TAG50:5-FA18:1 |
|  |  | TAG50:5-FA18:2 |
|  |  | TAG50:5-FA18:3 |
|  |  | TAG50:5-FA20:4 |
|  |  | TAG50:5-FA20:5 |
|  |  | TAG50:6-FA20:4 |
|  |  | TAG51:0-FA16:0 |
|  |  | TAG51:0-FA17:0 |
|  |  | TAG51:0-FA18:0 |
|  |  | TAG51:1-FA16:0 |
|  |  | TAG51:1-FA17:0 |
|  |  | TAG51:1-FA18:0 |
|  |  | TAG51:1-FA18:1 |
|  |  | TAG51:2-FA16:0 |
|  |  | TAG51:2-FA16:1 |
|  |  | TAG51:2-FA17:0 |
|  |  | TAG51:2-FA18:1 |
|  |  | TAG51:2-FA18:2 |
|  |  | TAG51:3-FA16:1 |
|  |  | TAG51:3-FA17:0 |
|  |  | TAG51:3-FA18:2 |
|  |  | TAG51:3-FA18:3 |
|  |  | TAG51:4-FA16:1 |
|  |  | TAG51:4-FA18:2 |
|  |  | TAG51:4-FA18:3 |
|  |  | TAG51:4-FA20:4 |
|  |  | TAG51:5-FA18:2 |
|  |  | TAG51:5-FA18:3 |
|  |  | TAG52:0-FA16:0 |
|  |  | TAG52:0-FA18:0 |
|  |  | TAG52:0-FA20:0 |
|  |  | TAG52:1-FA16:0 |
|  |  | TAG52:1-FA16:1 |
|  |  | TAG52:1-FA18:0 |
|  |  | TAG52:1-FA18:1 |
|  |  | TAG52:1-FA20:0 |
|  |  | TAG52:1-FA20:1 |
|  |  | TAG52:2-FA14:0 |
|  |  | TAG52:2-FA16:0 |
|  |  | TAG52:2-FA16:1 |
|  |  | TAG52:2-FA18:0 |
|  |  | TAG52:2-FA18:1 |
|  |  | TAG52:2-FA18:2 |
|  |  | TAG52:2-FA20:0 |
|  |  | TAG52:2-FA20:1 |
|  |  | TAG52:2-FA20:2 |
|  |  | TAG52:3-FA14:0 |
|  |  | TAG52:3-FA16:0 |
|  |  | TAG52:3-FA16:1 |
|  |  | TAG52:3-FA18:0 |
|  |  | TAG52:3-FA18:1 |
|  |  | TAG52:3-FA18:2 |
|  |  | TAG52:3-FA18:3 |
|  |  | TAG52:3-FA20:0 |
|  |  | TAG52:3-FA20:1 |
|  |  | TAG52:3-FA20:2 |
|  |  | TAG52:3-FA20:3 |
|  |  | TAG52:4-FA14:0 |
|  |  | TAG52:4-FA16:0 |
|  |  | TAG52:4-FA16:1 |
|  |  | TAG52:4-FA18:0 |
|  |  | TAG52:4-FA18:1 |
|  |  | TAG52:4-FA18:2 |
|  |  | TAG52:4-FA18:3 |
|  |  | TAG52:4-FA20:0 |
|  |  | TAG52:4-FA20:2 |
|  |  | TAG52:4-FA20:3 |
|  |  | TAG52:4-FA20:4 |
|  |  | TAG52:4-FA22:4 |
|  |  | TAG52:5-FA14:0 |
|  |  | TAG52:5-FA16:0 |
|  |  | TAG52:5-FA16:1 |
|  |  | TAG52:5-FA18:1 |
|  |  | TAG52:5-FA18:2 |
|  |  | TAG52:5-FA18:3 |
|  |  | TAG52:5-FA20:3 |
|  |  | TAG52:5-FA20:4 |
|  |  | TAG52:5-FA20:5 |
|  |  | TAG52:5-FA22:5 |
|  |  | TAG52:6-FA14:0 |
|  |  | TAG52:6-FA16:0 |
|  |  | TAG52:6-FA16:1 |
|  |  | TAG52:6-FA18:2 |
|  |  | TAG52:6-FA18:3 |
|  |  | TAG52:6-FA20:4 |
|  |  | TAG52:6-FA20:5 |
|  |  | TAG52:6-FA22:6 |
|  |  | TAG52:7-FA16:0 |
|  |  | TAG52:7-FA18:1 |
|  |  | TAG52:7-FA20:5 |
|  |  | TAG52:7-FA22:6 |
|  |  | TAG52:8-FA16:1 |
|  |  | TAG52:8-FA18:2 |
|  |  | TAG53:0-FA16:0 |
|  |  | TAG53:1-FA16:0 |
|  |  | TAG53:1-FA17:0 |
|  |  | TAG53:1-FA18:0 |
|  |  | TAG53:1-FA18:1 |
|  |  | TAG53:2-FA16:0 |
|  |  | TAG53:2-FA17:0 |
|  |  | TAG53:2-FA18:1 |
|  |  | TAG53:2-FA18:2 |
|  |  | TAG53:3-FA16:0 |
|  |  | TAG53:3-FA17:0 |
|  |  | TAG53:3-FA18:2 |
|  |  | TAG53:4-FA16:0 |
|  |  | TAG53:4-FA17:0 |
|  |  | TAG53:4-FA18:2 |
|  |  | TAG53:4-FA18:3 |
|  |  | TAG53:4-FA20:4 |
|  |  | TAG53:5-FA20:4 |
|  |  | TAG53:6-FA20:4 |
|  |  | TAG54:0-FA16:0 |
|  |  | TAG54:0-FA18:0 |
|  |  | TAG54:1-FA16:0 |
|  |  | TAG54:1-FA18:0 |
|  |  | TAG54:1-FA18:1 |
|  |  | TAG54:1-FA20:0 |
|  |  | TAG54:1-FA20:1 |
|  |  | TAG54:2-FA16:0 |
|  |  | TAG54:2-FA18:0 |
|  |  | TAG54:2-FA18:1 |
|  |  | TAG54:2-FA18:2 |
|  |  | TAG54:2-FA20:0 |
|  |  | TAG54:2-FA20:1 |
|  |  | TAG54:2-FA20:2 |
|  |  | TAG54:3-FA16:0 |
|  |  | TAG54:3-FA16:1 |
|  |  | TAG54:3-FA18:0 |
|  |  | TAG54:3-FA18:1 |
|  |  | TAG54:3-FA18:2 |
|  |  | TAG54:3-FA18:3 |
|  |  | TAG54:3-FA20:1 |
|  |  | TAG54:3-FA20:2 |
|  |  | TAG54:3-FA20:3 |
|  |  | TAG54:4-FA16:0 |
|  |  | TAG54:4-FA16:1 |
|  |  | TAG54:4-FA18:0 |
|  |  | TAG54:4-FA18:1 |
|  |  | TAG54:4-FA18:2 |
|  |  | TAG54:4-FA18:3 |
|  |  | TAG54:4-FA20:1 |
|  |  | TAG54:4-FA20:2 |
|  |  | TAG54:4-FA20:3 |
|  |  | TAG54:4-FA20:4 |
|  |  | TAG54:4-FA22:4 |
|  |  | TAG54:5-FA16:0 |
|  |  | TAG54:5-FA16:1 |
|  |  | TAG54:5-FA18:0 |
|  |  | TAG54:5-FA18:1 |
|  |  | TAG54:5-FA18:2 |
|  |  | TAG54:5-FA18:3 |
|  |  | TAG54:5-FA20:2 |
|  |  | TAG54:5-FA20:3 |
|  |  | TAG54:5-FA20:4 |
|  |  | TAG54:5-FA20:5 |
|  |  | TAG54:5-FA22:4 |
|  |  | TAG54:5-FA22:5 |
|  |  | TAG54:6-FA16:0 |
|  |  | TAG54:6-FA16:1 |
|  |  | TAG54:6-FA18:1 |
|  |  | TAG54:6-FA18:2 |
|  |  | TAG54:6-FA18:3 |
|  |  | TAG54:6-FA20:3 |
|  |  | TAG54:6-FA20:4 |
|  |  | TAG54:6-FA20:5 |
|  |  | TAG54:6-FA22:5 |
|  |  | TAG54:6-FA22:6 |
|  |  | TAG54:7-FA16:1 |
|  |  | TAG54:7-FA18:1 |
|  |  | TAG54:7-FA18:2 |
|  |  | TAG54:7-FA18:3 |
|  |  | TAG54:7-FA20:4 |
|  |  | TAG54:7-FA20:5 |
|  |  | TAG54:7-FA22:5 |
|  |  | TAG54:7-FA22:6 |
|  |  | TAG54:8-FA18:2 |
|  |  | TAG54:8-FA18:3 |
|  |  | TAG54:8-FA20:4 |
|  |  | TAG54:8-FA20:5 |
|  |  | TAG54:8-FA22:6 |
|  |  | TAG55:1-FA16:0 |
|  |  | TAG55:1-FA18:1 |
|  |  | TAG55:2-FA18:1 |
|  |  | TAG55:2-FA18:2 |
|  |  | TAG55:3-FA18:1 |
|  |  | TAG55:3-FA18:2 |
|  |  | TAG55:4-FA18:1 |
|  |  | TAG55:4-FA18:2 |
|  |  | TAG55:5-FA18:1 |
|  |  | TAG55:5-FA18:2 |
|  |  | TAG55:5-FA20:4 |
|  |  | TAG55:7-FA22:6 |
|  |  | TAG56:10-FA18:2 |
|  |  | TAG56:1-FA16:0 |
|  |  | TAG56:1-FA18:1 |
|  |  | TAG56:2-FA16:0 |
|  |  | TAG56:2-FA18:0 |
|  |  | TAG56:2-FA20:0 |
|  |  | TAG56:2-FA20:1 |
|  |  | TAG56:3-FA16:0 |
|  |  | TAG56:3-FA18:0 |
|  |  | TAG56:3-FA18:1 |
|  |  | TAG56:3-FA18:2 |
|  |  | TAG56:3-FA20:0 |
|  |  | TAG56:3-FA20:1 |
|  |  | TAG56:3-FA20:2 |
|  |  | TAG56:4-FA16:0 |
|  |  | TAG56:4-FA18:0 |
|  |  | TAG56:4-FA18:1 |
|  |  | TAG56:4-FA18:2 |
|  |  | TAG56:4-FA20:1 |
|  |  | TAG56:4-FA20:2 |
|  |  | TAG56:4-FA20:3 |
|  |  | TAG56:4-FA20:4 |
|  |  | TAG56:4-FA22:4 |
|  |  | TAG56:5-FA16:0 |
|  |  | TAG56:5-FA18:0 |
|  |  | TAG56:5-FA18:1 |
|  |  | TAG56:5-FA18:2 |
|  |  | TAG56:5-FA20:1 |
|  |  | TAG56:5-FA20:2 |
|  |  | TAG56:5-FA20:3 |
|  |  | TAG56:5-FA20:4 |
|  |  | TAG56:5-FA22:4 |
|  |  | TAG56:5-FA22:5 |
|  |  | TAG56:6-FA16:0 |
|  |  | TAG56:6-FA18:0 |
|  |  | TAG56:6-FA18:1 |
|  |  | TAG56:6-FA18:2 |
|  |  | TAG56:6-FA18:3 |
|  |  | TAG56:6-FA20:2 |
|  |  | TAG56:6-FA20:3 |
|  |  | TAG56:6-FA20:4 |
|  |  | TAG56:6-FA20:5 |
|  |  | TAG56:6-FA22:4 |
|  |  | TAG56:6-FA22:5 |
|  |  | TAG56:6-FA22:6 |
|  |  | TAG56:7-FA16:0 |
|  |  | TAG56:7-FA16:1 |
|  |  | TAG56:7-FA18:0 |
|  |  | TAG56:7-FA18:1 |
|  |  | TAG56:7-FA18:2 |
|  |  | TAG56:7-FA18:3 |
|  |  | TAG56:7-FA20:3 |
|  |  | TAG56:7-FA20:4 |
|  |  | TAG56:7-FA20:5 |
|  |  | TAG56:7-FA22:4 |
|  |  | TAG56:7-FA22:5 |
|  |  | TAG56:7-FA22:6 |
|  |  | TAG56:8-FA16:0 |
|  |  | TAG56:8-FA16:1 |
|  |  | TAG56:8-FA18:1 |
|  |  | TAG56:8-FA18:2 |
|  |  | TAG56:8-FA18:3 |
|  |  | TAG56:8-FA20:4 |
|  |  | TAG56:8-FA20:5 |
|  |  | TAG56:8-FA22:5 |
|  |  | TAG56:8-FA22:6 |
|  |  | TAG56:9-FA18:3 |
|  |  | TAG56:9-FA20:4 |
|  |  | TAG56:9-FA20:5 |
|  |  | TAG56:9-FA22:6 |
|  |  | TAG57:2-FA18:1 |
|  |  | TAG57:3-FA18:2 |
|  |  | TAG58:10-FA18:2 |
|  |  | TAG58:10-FA20:4 |
|  |  | TAG58:10-FA20:5 |
|  |  | TAG58:10-FA22:5 |
|  |  | TAG58:10-FA22:6 |
|  |  | TAG58:2-FA18:1 |
|  |  | TAG58:3-FA18:1 |
|  |  | TAG58:5-FA18:1 |
|  |  | TAG58:6-FA16:0 |
|  |  | TAG58:6-FA18:0 |
|  |  | TAG58:6-FA18:1 |
|  |  | TAG58:6-FA20:4 |
|  |  | TAG58:6-FA22:4 |
|  |  | TAG58:6-FA22:5 |
|  |  | TAG58:7-FA16:0 |
|  |  | TAG58:7-FA18:0 |
|  |  | TAG58:7-FA18:1 |
|  |  | TAG58:7-FA18:2 |
|  |  | TAG58:7-FA20:4 |
|  |  | TAG58:7-FA22:4 |
|  |  | TAG58:7-FA22:5 |
|  |  | TAG58:7-FA22:6 |
|  |  | TAG58:8-FA18:1 |
|  |  | TAG58:8-FA18:2 |
|  |  | TAG58:8-FA20:3 |
|  |  | TAG58:8-FA20:4 |
|  |  | TAG58:8-FA22:5 |
|  |  | TAG58:8-FA22:6 |
|  |  | TAG58:9-FA18:1 |
|  |  | TAG58:9-FA18:2 |
|  |  | TAG58:9-FA20:4 |
|  |  | TAG58:9-FA22:5 |
|  |  | TAG58:9-FA22:6 |
|  |  | TAG60:10-FA22:5 |
|  |  | TAG60:10-FA22:6 |
|  |  | TAG60:11-FA22:5 |
|  |  | TAG60:11-FA22:6 |
|  |  | TAG60:12-FA22:6 |
| Phospholipids | Phosphatidylcholine (PC) | PC(14:0/14:0) |
|  |  | PC(14:0/18:1) |
|  |  | PC(14:0/18:2) |
|  |  | PC(14:0/18:3) |
|  |  | PC(14:0/20:2) |
|  |  | PC(14:0/20:3) |
|  |  | PC(14:0/20:4) |
|  |  | PC(14:0/20:5) |
|  |  | PC(14:0/22:5) |
|  |  | PC(14:0/22:6) |
|  |  | PC(16:0/14:0) |
|  |  | PC(16:0/16:0) |
|  |  | PC(16:0/16:1) |
|  |  | PC(16:0/18:0) |
|  |  | PC(16:0/18:1) |
|  |  | PC(16:0/18:3) |
|  |  | PC(16:0/20:1) |
|  |  | PC(16:0/20:2) |
|  |  | PC(16:0/20:3) |
|  |  | PC(16:0/20:5) |
|  |  | PC(16:0/22:4) |
|  |  | PC(16:0/22:5) |
|  |  | PC(16:0/22:6) |
|  |  | PC(16:1/18:2) |
|  |  | PC(18:0/14:0) |
|  |  | PC(18:0/16:1) |
|  |  | PC(18:0/18:0) |
|  |  | PC(18:0/18:1) |
|  |  | PC(18:0/18:3) |
|  |  | PC(18:0/20:0) |
|  |  | PC(18:0/20:1) |
|  |  | PC(18:0/20:2) |
|  |  | PC(18:0/20:3) |
|  |  | PC(18:0/20:4) |
|  |  | PC(18:0/20:5) |
|  |  | PC(18:0/22:4) |
|  |  | PC(18:0/22:5) |
|  |  | PC(18:0/22:6) |
|  |  | PC(18:1/18:1) |
|  |  | PC(18:1/18:2) |
|  |  | PC(18:1/18:3) |
|  |  | PC(18:1/20:1) |
|  |  | PC(18:1/20:2) |
|  |  | PC(18:1/20:3) |
|  |  | PC(18:1/20:4) |
|  |  | PC(18:1/20:5) |
|  |  | PC(18:1/22:4) |
|  |  | PC(18:1/22:5) |
|  |  | PC(18:1/22:6) |
|  |  | PC(18:2/16:1) |
|  |  | PC(18:2/18:2) |
|  |  | PC(18:2/18:3) |
|  |  | PC(18:2/20:1) |
|  |  | PC(18:2/20:2) |
|  |  | PC(18:2/20:3) |
|  |  | PC(18:2/20:4) |
|  |  | PC(18:2/20:5) |
|  |  | PC(18:2/22:4) |
|  |  | PC(18:2/22:5) |
|  |  | PC(18:2/22:6) |
|  |  | PC(20:0/18:1) |
|  |  | PC(20:0/20:3) |
|  |  | PC(20:0/20:4) |
|  |  | PC(20:0/22:4) |
|  | Phosphatidylethanolamine (PE) | PE(14:0/18:1) |
|  |  | PE(16:0/16:0) |
|  |  | PE(16:0/16:1) |
|  |  | PE(16:0/18:1) |
|  |  | PE(16:0/18:2) |
|  |  | PE(16:0/18:3) |
|  |  | PE(16:0/20:1) |
|  |  | PE(16:0/20:2) |
|  |  | PE(16:0/20:3) |
|  |  | PE(16:0/20:4) |
|  |  | PE(16:0/20:5) |
|  |  | PE(16:0/22:4) |
|  |  | PE(16:0/22:5) |
|  |  | PE(16:0/22:6) |
|  |  | PE(18:0/16:0) |
|  |  | PE(18:0/16:1) |
|  |  | PE(18:0/18:0) |
|  |  | PE(18:0/18:1) |
|  |  | PE(18:0/18:2) |
|  |  | PE(18:0/18:3) |
|  |  | PE(18:0/20:1) |
|  |  | PE(18:0/20:2) |
|  |  | PE(18:0/20:3) |
|  |  | PE(18:0/20:5) |
|  |  | PE(18:0/22:4) |
|  |  | PE(18:0/22:5) |
|  |  | PE(18:0/22:6) |
|  |  | PE(18:1/16:1) |
|  |  | PE(18:1/18:1) |
|  |  | PE(18:1/18:2) |
|  |  | PE(18:1/18:3) |
|  |  | PE(18:1/20:1) |
|  |  | PE(18:1/20:2) |
|  |  | PE(18:1/20:3) |
|  |  | PE(18:1/20:4) |
|  |  | PE(18:1/20:5) |
|  |  | PE(18:1/22:4) |
|  |  | PE(18:1/22:5) |
|  |  | PE(18:1/22:6) |
|  |  | PE(18:2/16:1) |
|  |  | PE(18:2/18:2) |
|  |  | PE(18:2/18:3) |
|  |  | PE(18:2/20:1) |
|  |  | PE(18:2/20:3) |
|  |  | PE(18:2/20:4) |
|  |  | PE(18:2/22:6) |
|  | Phosphatidylethanolamine-O (PE-O) | PE(O-16:0/16:0) |
|  |  | PE(O-16:0/18:1) |
|  |  | PE(O-16:0/18:2) |
|  |  | PE(O-16:0/20:3) |
|  |  | PE(O-16:0/20:4) |
|  |  | PE(O-16:0/20:5) |
|  |  | PE(O-16:0/22:4) |
|  |  | PE(O-16:0/22:5) |
|  |  | PE(O-16:0/22:6) |
|  |  | PE(O-18:0/16:0) |
|  |  | PE(O-18:0/18:0) |
|  |  | PE(O-18:0/18:1) |
|  |  | PE(O-18:0/18:2) |
|  |  | PE(O-18:0/20:3) |
|  |  | PE(O-18:0/20:4) |
|  |  | PE(O-18:0/20:5) |
|  |  | PE(O-18:0/22:4) |
|  |  | PE(O-18:0/22:5) |
|  |  | PE(O-18:0/22:6) |
|  | Phosphatidylethanolamine-P (PE-P) | PE(P-16:0/16:0) |
|  |  | PE(P-16:0/16:1) |
|  |  | PE(P-16:0/18:0) |
|  |  | PE(P-16:0/18:1) |
|  |  | PE(P-16:0/18:3) |
|  |  | PE(P-16:0/20:1) |
|  |  | PE(P-16:0/20:2) |
|  |  | PE(P-16:0/20:3) |
|  |  | PE(P-16:0/20:5) |
|  |  | PE(P-16:0/22:4) |
|  |  | PE(P-16:0/22:5) |
|  |  | PE(P-16:0/22:6) |
|  |  | PE(P-16:1/18:1) |
|  |  | PE(P-18:0/16:0) |
|  |  | PE(P-18:0/16:1) |
|  |  | PE(P-18:0/18:0) |
|  |  | PE(P-18:0/18:1) |
|  |  | PE(P-18:0/18:2) |
|  |  | PE(P-18:0/18:3) |
|  |  | PE(P-18:0/20:2) |
|  |  | PE(P-18:0/20:3) |
|  |  | PE(P-18:0/20:4) |
|  |  | PE(P-18:0/22:4) |
|  |  | PE(P-18:0/22:5) |
|  |  | PE(P-18:0/22:6) |
|  |  | PE(P-18:1/16:0) |
|  |  | PE(P-18:1/16:1) |
|  |  | PE(P-18:1/18:1) |
|  |  | PE(P-18:1/18:2) |
|  |  | PE(P-18:1/18:3) |
|  |  | PE(P-18:1/20:1) |
|  |  | PE(P-18:1/20:2) |
|  |  | PE(P-18:1/20:3) |
|  |  | PE(P-18:1/20:4) |
|  |  | PE(P-18:1/20:5) |
|  |  | PE(P-18:1/22:4) |
|  |  | PE(P-18:1/22:5) |
|  |  | PE(P-18:1/22:6) |
|  |  | PE(P-18:2/18:2) |
|  |  | PE(P-18:2/20:4) |
|  |  | PE(P-18:2/22:6) |
|  | Lysophosphatidylcholine (LPC) | LPC(16:0) |
|  |  | LPC(16:1) |
|  |  | LPC(18:0) |
|  |  | LPC(18:2) |
|  |  | LPC(20:0) |
|  |  | LPC(20:1) |
|  |  | LPC(20:2) |
|  |  | LPC(20:3) |
|  |  | LPC(20:4) |
|  |  | LPC(22:4) |
|  |  | LPC(22:5) |
|  | Lysophosphatidylethanolamine (LPE) | LPE(16:0) |
|  |  | LPE(18:0) |
|  |  | LPE(18:1) |
|  |  | LPE(18:2) |
|  |  | LPE(20:1) |
|  |  | LPE(20:2) |
|  |  | LPE(20:3) |
|  |  | LPE(20:4) |
|  |  | LPE(22:4) |
|  |  | LPE(22:5) |
|  | Phosphatidylinositol (PI) | PI(14:0/18:2) |
|  |  | PI(14:0/20:4) |
|  |  | PI(16:0/18:1) |
|  |  | PI(16:0/18:2) |
|  |  | PI(16:0/18:3) |
|  |  | PI(16:0/20:4) |
|  |  | PI(18:0/18:1) |
|  |  | PI(18:0/20:0) |
|  |  | PI(18:0/20:3) |
|  |  | PI(18:1/16:1) |
|  |  | PI(18:1/18:1) |
|  |  | PI(18:1/18:2) |
|  |  | PI(18:1/20:4) |
|  |  | PI(18:2/16:1) |
|  |  | PI(18:2/18:2) |
|  |  | PI(18:2/20:3) |
|  |  | PI(18:2/20:4) |
|  |  | PI(20:0/16:1) |
|  |  | PI(20:0/18:1) |
|  |  | PI(20:0/18:2) |
|  |  | PI(20:0/20:4) |
|  | Phosphatidylglycerol (PG) | PG(14:0/18:2) |
|  |  | PG(14:0/20:4) |
|  |  | PG(16:0/16:0) |
|  |  | PG(16:0/18:2) |
|  |  | PG(16:0/20:3) |
|  |  | PG(16:0/20:5) |
|  |  | PG(16:0/22:5) |
|  |  | PG(18:0/18:1) |
|  |  | PG(18:0/20:0) |
|  |  | PG(18:1/16:1) |
|  |  | PG(18:1/18:1) |
|  |  | PG(18:1/18:2) |
|  |  | PG(18:1/20:3) |
|  |  | PG(18:2/16:1) |
|  |  | PG(18:2/20:4) |
|  |  | PG(20:0/16:1) |
|  |  | PG(20:0/18:1) |
|  |  | PG(20:0/18:2) |
|  |  | PG(20:0/20:2) |
|  |  | PG(20:0/20:3) |
|  |  | PG(20:0/20:4) |
|  |  | PG(20:0/22:4) |
|  |  | PG(20:0/22:5) |
|  |  | PG(20:0/22:6) |
|  | Phosphatidylserine (PS) | PS(14:0/18:1) |
|  |  | PS(14:0/20:4) |
|  |  | PS(16:0/18:0) |
|  |  | PS(16:0/18:2) |
|  |  | PS(18:0/18:0) |
|  |  | PS(18:0/20:0) |
|  |  | PS(18:0/20:1) |
|  |  | PS(18:0/20:3) |
|  |  | PS(18:0/20:4) |
|  |  | PS(18:1/18:2) |
|  |  | PS(18:1/20:3) |
|  |  | PS(18:1/20:4) |
|  |  | PS(18:2/16:1) |
|  |  | PS(18:2/20:4) |
|  |  | PS(20:0/16:1) |
|  |  | PS(20:0/18:1) |
|  |  | PS(20:0/18:2) |
|  |  | PS(20:0/18:3) |
|  |  | PS(20:0/20:2) |
|  |  | PS(20:0/20:3) |
|  |  | PS(20:0/20:4) |
|  |  | PS(20:0/20:5) |
|  |  | PS(20:0/22:4) |
|  |  | PS(20:0/22:5) |
|  |  | PS(20:0/22:6) |
|  | Lysophosphatidylserine (LPS) | LPS(22:6) |
| Sphingolipids | Sphingomyelin (SM) | SM(14:0) |
|  |  | SM(20:1) |
|  |  | SM(22:1) |
|  |  | SM 30:0 |
|  |  | SM 32:0 |
|  |  | SM 34:1 |
|  |  | SM 34:2 |
|  |  | SM 36:1 |
|  |  | SM 36:2 |
|  |  | SM 38:0 |
|  |  | SM 38:1 |
|  |  | SM 38:2 |
|  |  | SM 38:4 |
|  |  | SM 40:1 |
|  |  | SM 40:2 |
|  |  | SM 40:4 |
|  |  | SM 42:1 |
|  |  | SM 42:2 |
|  |  | SM 42:4 |
|  |  | SM 42:5 |
|  |  | SM 44:1 |
|  |  | SM 44:2 |
|  | Ceramide (CER) | CER(14:0) |
|  |  | CER(16:0) |
|  |  | CER(18:0) |
|  |  | CER(20:0) |
|  |  | CER(22:0) |
|  |  | CER(24:0) |
|  |  | CER(26:0) |
|  |  | CER(18:1) |
|  |  | CER(24:1) |
|  |  | CER(20:4) |
|  | Dihydroceramide (DCER) | DCER(16:0) |
|  |  | DCER(18:0) |
|  |  | DCER(22:0) |
|  |  | DCER(22:1) |
|  |  | DCER(24:0) |
|  |  | DCER(24:1) |
|  | Hexosylceramide (HCER) | HCER(16:0) |
|  |  | HCER(18:0) |
|  |  | HCER(18:1) |
|  |  | HCER(20:0) |
|  |  | HCER(20:1) |
|  |  | HCER(22:0) |
|  |  | HCER(22:1) |
|  |  | HCER(24:0) |
|  |  | HCER(24:1) |
|  | Lactosylceramide (LCER) | LCER(16:0) |
|  |  | LCER(18:0) |
|  |  | LCER(20:1) |
|  |  | LCER(22:1) |
|  |  | LCER(24:0) |
|  |  | LCER(24:1) |

# **Table S2:** Differentially Altered Lipids in CKD Macrophages and Plasma


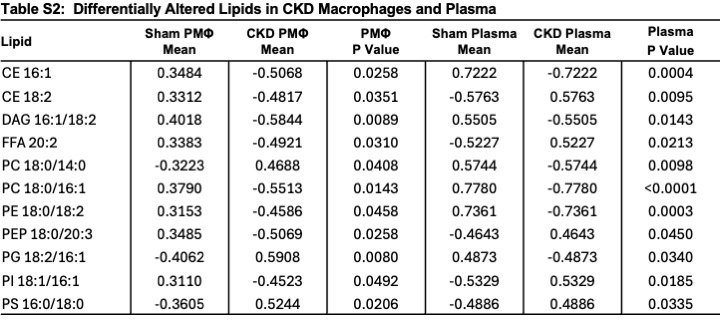

Supplement: Supplemental Material [file mmc1.docx]
